# Organ donation and transplantation in Canada: insights from the Canadian Organ Replacement Register

**DOI:** 10.1186/s40697-014-0031-8

**Published:** 2014-12-09

**Authors:** Sang Joseph Kim, Stanley SA Fenton, Joanne Kappel, Louise M Moist, Scott W Klarenbach, Susan M Samuel, Lianne G Singer, Daniel H Kim, Kimberly Young, Greg Webster, Juliana Wu, Frank Ivis, Eric de Sa, John S Gill

**Affiliations:** Department of Medicine, Division of Nephrology, University of Toronto, 585 University Avenue, 11-PMB-129, Toronto, ON M5G 2 N2 Canada; Multi-Organ Transplant Program, University Health Network, Toronto, ON Canada; Department of Medicine, Division of Nephrology, University of Saskatchewan, Saskatoon, SK Canada; Department of Medicine, Division of Nephrology, Western University, London, ON Canada; Department of Medicine, Division of Nephrology, University of Alberta, Edmonton, AB Canada; Department of Pediatrics, Division of Nephrology, University of Calgary, Calgary, AB Canada; Department of Medicine, Division of Respirology, University of Toronto, Toronto, ON Canada; Department of Medicine, Division of Cardiology, University of Alberta, Edmonton, AB Canada; Donation and Transplantation, Canadian Blood Services, Ottawa, ON Canada; Canadian Institute for Health Information, Toronto, ON Canada; Department of Medicine, Division of Nephrology, University of British Columbia, Vancouver, BC Canada

**Keywords:** Canada, Registry, Organ donation, Transplantation, Wait-listing

## Abstract

**Purpose of review:**

To provide an overview of the transplant component of the Canadian Organ Replacement Register (CORR).

**Findings:**

CORR is the national registry of organ failure in Canada. It has existed in some form since 1972 and currently houses data on patients with end-stage renal disease and solid organ transplants (kidney and/or non-kidney). The transplant component of CORR receives data on a voluntary basis from individual transplant centres and organ procurement organizations across the country. Coverage for transplant procedures is comprehensive and complete. Long-term outcomes are tracked based on follow-up reports from participating transplant centres. The longitudinal nature of CORR provides an opportunity to observe the trajectory of a patient’s journey with organ failure over their life span. Research studies conducted using CORR data inform both practitioners and health policy makers alike.

**Implications:**

The importance of registry data in monitoring and improving care for Canadian transplant candidates/recipients cannot be over-stated. This paper provides an overview of the transplant data in CORR including its history, data considerations, recent findings, new initiatives, and future directions.

**Electronic supplementary material:**

The online version of this article (doi:10.1186/s40697-014-0031-8) contains supplementary material, which is available to authorized users.

## ABRÉGÉ

**But de la revue:**

Offrir un aperçu du volet « transplantation d’organes » du Registre canadien des insuffisances et des transplantations d’organes (RCITO).

**Résultats:**

Le RCITO est le Registre canadien des insuffisances d’organes au Canada. Il a commencé à prendre forme en 1972, et contient à l’heure actuelle des données sur des patients atteints de néphropathie terminale et sur des transplantations (rénales ou non rénales) d’organes pleins. Le volet « transplantation d’organes » du RCITO collige des données qui ont été envoyées, sur une base volontaire, par des centres de transplantation et des services d’approvisionnement en organes à travers le pays. Le Registre offre une couverture exhaustive et complète des différentes interventions de transplantation. Les résultats à long terme sont retracés à partir de rapports de rendez-vous de suivi des centres de transplantation participants. L’ampleur longitudinale du RCITO offre la possibilité d’observer le parcours, tout au long de sa vie, du patient atteint d’une insuffisance organique. Les études produites à partir des données du RCITO éclairent à la fois les praticiens et les décideurs du domaine de la santé.

**Implications:**

On ne peut surestimer l’importance des données du Registre lorsqu’il s’agit d’effectuer le suivi des candidats canadiens potentiels à une transplantation, ou d’améliorer les soins qui leur sont offerts.

Cette revue offre un aperçu des données du RCITO qui se rapportent à la transplantation d’organes, dont : l’historique, les éléments à considérer sur les données, des résultats récents, de nouvelles initiatives et les orientations futures.

## What was known before

The Canadian Organ Replacement Register (CORR) is the national registry of organ failure and contains information on dialysis, donation, and organ transplantation in Canada. However, the details of CORR’s transplant data holdings and the future directions of the registry are not widely known.

## What this adds

This review provides a description of CORR, its transplant-related data holdings, the results of analyses using these data, and the future direction of the registry.

## Background

The Canadian Organ Replacement Register (CORR) is the national registry for organ failure in Canada. The scope of CORR includes all incident and prevalent end-stage kidney disease (ESKD) patients on dialysis, organ donors (deceased and living), and recipients of kidney and/or non-kidney organ transplants. CORR is a longitudinal database, i.e., all kidney patients are tracked from the time of dialysis initiation or transplantation to dialysis modality switches, organ failure, and/or death. Non-kidney solid organ transplant patients are tracked from the time of transplantation. The CORR Board of Directors (comprised of physicians, surgeons, allied health professionals, data scientists, patient advocates, and patients) is responsible for providing strategic advice to the CORR team at the Canadian Institute for Health Information (CIHI). CIHI oversees CORR operations including data collection, management, analytics, and reporting.

This report provides an overview of the transplantation component of CORR. A detailed review of the dialysis component of CORR has also been published [1].

### History

CORR originally started as a renal failure registry in 1972 under the leadership of Dr. Arthur Shimizu [2]. In 1973, the registry transferred to Statistics Canada, in collaboration with the Kidney Foundation of Canada. Its first report was published in 1974, and in subsequent years, more detailed reports of dialysis and kidney transplantation activity were produced. In 1980, CORR developed a new partnership with the Kidney Foundation of Canada, Health Canada, and Statistics Canada, with guidance from the Canadian Society of Nephrology. Information on extra-renal solid organ transplants became a part of CORR’s data holdings in 1987. This was initiated after the Advisory Committee on Institutional and Medical Services made a recommendation in 1985 that CORR become a fully functional organ failure registry. Data on extra-renal solid organ transplants performed prior to 1987 were also retroactively collected, although not systematically. Prospective data collection on all incident extra-renal solid organ transplants started on January 1, 1988. All kidney transplants in patients initiating renal replacement therapy in Canada from January 1, 1981 onward are captured in CORR.

## Methods

### Data considerations

Data for the transplant portion of CORR are voluntarily submitted to CIHI by all Canadian transplant centres, either directly or through their provincial and regional organ procurement organization (OPO). A total of 25 kidney, 9 liver, 11 heart, 6 lung, 8 pancreas, 3 intestinal/multi-visceral, and 2 islet transplant programs are represented in CORR. Data submission occurs twice a year. After the completion of data entry at CIHI, reporting centres are provided standardized audit reports for the purposes of data verification. At present, the CORR annual data file is available approximately 9 months after year-end and the CORR annual analytical report is published within 11 months following data year-end. Only transplants performed in Canada are reported by CORR. However, out-of-country transfers are also captured when reported by transplant facilities.

Newly transplanted patients are reported to CORR via organ-specific registration forms. This includes baseline demographic and clinical information as well as events in the early post-transplant period (e.g., delayed graft function in kidney transplant recipients). Forms detailing the characteristics of deceased and living donors are typically completed by the OPO and transplant centres, respectively. Follow-up events, including return to dialysis, re-transplantation, graft failure, or death, are reported to CORR using the follow-up or outcome forms for each organ transplant group. Coverage of transplant procedures in CORR has been evaluated against the CIHI Discharge Abstract Database and found to have 98.5% agreement despite voluntary reporting by transplant centres [2]. The completeness of follow-up data in CORR has not undergone a similar validation process. Moreover, certain data elements have historically displayed greater degrees of missingness (e.g., race/ethnicity, cause of death, cause of graft failure) than other data elements [2]. Further details on CORR data quality can be found in Appendix D of the 2014 CORR Annual Report (www.cihi.ca/corr). All of the CORR reporting forms for 2014 are provided in the Additional file 1. Each data element is explicitly defined in a CORR instruction manual provided by CIHI to all participating centres.

CORR does not currently receive patient-level data for those wait-listed for transplantation. Aggregate counts of patients waiting for solid organ transplants are provided on a semi-annual basis by the OPO responsible for maintaining these waiting lists. This allows for the tracking of total numbers of patients on the waiting list at the end of each calendar year as well as the number of deaths over the year. The OPO contributing wait-list counts include BC Transplant, Southern Alberta Organ and Tissue Donation Program (Calgary), HOPE Edmonton, the Saskatchewan Transplant Program (Saskatoon and Regina), Transplant Manitoba Gift of Life, the Trillium Gift of Life Network (Ontario), Transplant Québec and the Nova Scotia Multi-Organ Transplant Program (for the Atlantic region). A recent effort to collect patient-level data from the time of referral to transplantation has been undertaken in the ESKD population (see below).

## Results

### Organ donation in Canada

In CORR, deceased organ donors are defined as donors from whom at least one organ was recovered and transplanted. This definition is more conservative than that used by the Organ Procurement and Transplantation Network (U.S.) and Eurotransplant (Europe), which includes donors whose organs were recovered, but not transplanted. This is an important distinction to consider when making comparisons of deceased donor rates between countries. Moreover, CORR’s definition precludes the ability to quantify rates of organ discard from donors whose organs were recovered but not used.

**Figure 1:**
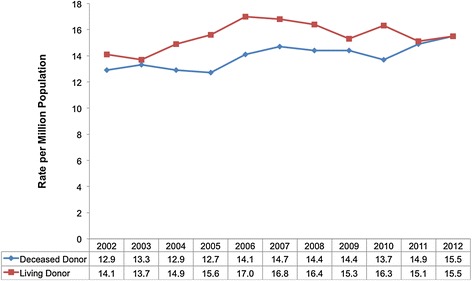
**Canadian donor rate per million population by donor type.**

The deceased donation rate has shown steady improvement in Canada, rising from 12.9 per million population (PMP) in 2002 to 15.5 PMP in 2012 (Figure 1) with gains in donors from neurological determination of death (NDD) and donation after cardio-circulatory death (DCD). DCD have doubled from 40 donors in 2008 to 82 donors in 2012 and currently makes up 14% of total deceased donations (target 25%). The living donor rate has been stable over the last 10 years; in 2012, the living and deceased donation rates were the same (i.e., 15.5 PMP).

**Figure 2:**
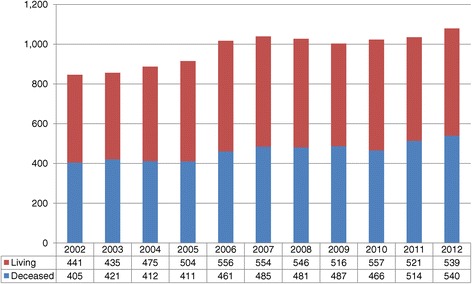
**Total number of Canadian donors by donor type.**

**Figure 3:**
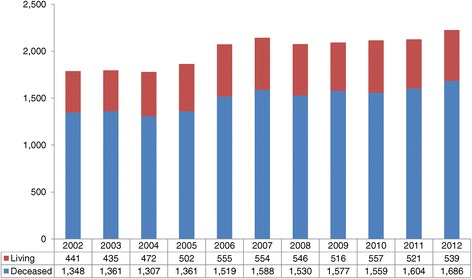
**Total number of Canadian solid organ transplant recipients by donor type.**

**Figure 4:**
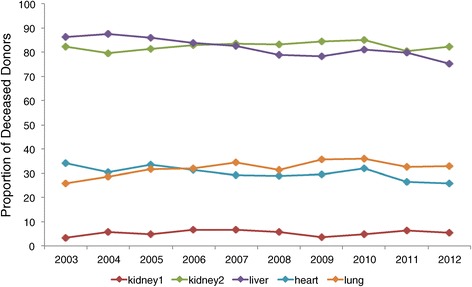
**Organ utilization for transplantation among deceased donors by year.**

A closer look at the general increase in Canadian deceased donation activity shows that certain regions such as Quebec, Ontario, B.C., and Atlantic Canada have made and sustained modest gains over the last 10 years while other provinces have remained stable in their activity or have shown some decline (Table 1). Specifically, Nova Scotia and Newfoundland have achieved over 22 donors PMP, which is the deceased donor performance target reported in the 2011 Call to Action report [3]. There were 540 deceased donors and 539 living donors in 2012; it is the first time that the former has exceeded the latter (Figure 2). This translated into 1,686 deceased and 539 living donor kidney and/or non-kidney transplants in 2012. This represents a 25% increase in deceased donor transplants and a 22% increase in living donor transplants when compared to 2002 (Figure 3). During this time, the rate of organ utilization per deceased donor has remained stable for kidneys, livers, hearts, and lungs (Figure 4).

**Table 1:** **Deceased donor rate (Per Million Population) by region**

		**2002**	**2003**	**2004**	**2005**	**2006**	**2007**	**2008**	**2009**	**2010**	**2011**	**2012**
Canada		12.9	13.3	12.9	12.7	14.1	14.7	14.4	14.4	13.6	14.9	15.5
Province	B.C.	7.3	9.4	7.4	5.9	8.4	8.8	12.3	7.2	10.8	12.2	15.1
	Alta.	17.3	11.4	16.2	14.1	14.8	13.7	11.4	10.6	8.9	10.3	10.1
	Sask.	12.0	17.1	9.0	15.1	10.1	14	12.8	12.6	12.5	12.3	5.6
	Man.	9.5	10.3	6.0	5.1	11.0	12.6	11.6	11.5	15.4	7.2	9.5
	Ont.	11.3	11.6	12.3	11.8	13.6	15.5	13.6	16.7	15.1	16.3	18.7
	Que.	16.8	19	18	17.8	18.2	18.2	19.5	17.6	15.1	17.2	14.9
	N.B.	14.7	16	13.3	21.3	13.3	17.4	10.7	14.7	10.6	9.3	15.9
	N.S./P.E.I.	11.8	11.8	9.6	13.9	20.3	15	17.1	16	20.1	26.4	15.5
	N.L.	27.0	19.3	9.7	13.6	23.5	9.9	15.8	13.8	9.8	19.6	23.4

### Solid organ transplantation in Canada

#### Kidney transplantation

There were 3,428 prevalent kidney transplant candidates on the waiting list by the end of 2012, a 13.7% increase from 2001. Despite this growth in the prevalent waiting list, the mortality rate of kidney transplant candidates has remained stable at 2 to 3% per year (Additional file 1: Figure S1). The record number of wait-listed candidates was mirrored by the number of kidney transplants performed in 2012. A total of 1,245 patients received a kidney transplant in 2012, with an approximately 2/3 to 1/3 distribution of deceased to living donors. This represents an increase of 24.6% in kidney transplant activity from 2003 to 2012 (Additional file 1: Table S1). A total of 72 kidneys were simultaneously transplanted with non-kidney organs (other than pancreas) from 2003 to 2012. There has been no clear trend in this activity over time, with 5 to 11 simultaneous transplants performed annually (Additional file 1: Table S1).

Kidney transplant outcomes are excellent, with 1- and 5-year total graft survival (where graft failure is defined as return to dialysis, pre-emptive kidney transplant, or death with graft function) of 93.4% and 81.8% in deceased donor kidney transplants and 97.1 and 90.0% in living donor kidney transplants, respectively (Additional file 1: Figure S2). Death-censored graft survival at 1- and 5-years for deceased donor kidney transplants are 95.6% and 88.9% while living donor kidney transplants are 97.7% and 93.4%, respectively (Additional file 1: Figure S3). From 2003 to 2012, outcomes have modestly improved for deceased donor kidney transplants and remained stable for living donor kidney transplants (Additional file 1: Figures S4 to S7).

#### Liver transplantation

At the end of 2012, 492 patients were awaiting a liver transplant. Over the last decade, the year with the largest waiting list was 2006 at 723 patients (Additional file 1: Figure S8). Mortality rates on the liver transplant waiting list are 15 to 20% per year and have remained relatively stable. A total of 494 patients received a liver transplant in 2012, a 22.0% increase since 2003 (Additional file 1: Table S2). Living donor liver transplants represent about 16% of the total activity. The cumulative survival of liver transplant recipients ranges from 81.8 to 87.0% in the first year after liver transplant (as a function of age group) to 74.0 to 82.0% by the end of the fifth year post-transplant (Additional file 1: Figure S9). There has been little difference in the outcomes of deceased and living donor liver transplantation, although the cumulative survival of living donor liver recipients is numerically superior to deceased donor liver recipients (Additional file 1: Figure S10). Short and long-term liver transplant outcomes have shown an improving trend from 2003 to 2012 (Additional file 1: Figure S11).

#### Heart transplantation

There has been a steady rise in the number of patients wait-listed for a heart transplant in Canada over the last several years. In 2012, a total of 183 patients were waiting for a heart transplant, whereas 125 patients were waiting at the end of 2001 (Additional file 1: Figure S12). This represents a 46% increase in wait-list activity. There were 161 heart transplants performed in 2012 with 11% of recipients in the pediatric category (i.e., age 0 to 17 years). The peak year for transplant activity over the last decade was 2006 with 178 heart transplants (Additional file 1: Table S3). Notably, a marked decline in wait-list mortality has been observed over the last several years, from 32% in 2001 to 9% in 2012. The cumulative survival of heart transplant recipients ranges from 77.9 to 88.6% at 1-year and 75.0 to 83.9% at 5-years, depending on recipient age category (Additional file 1: Figure S13). There has been a trend toward improving heart transplant survival, at least to 3-years post-transplant (Additional file 1: Figure S14).

Some of the trends observed could be explained by increased utilization of Ventricular Assist Devices (VAD) [4], which may increase the number of patients surviving on the waiting list. Paradoxically, it may also help explain the decrease in the number of heart transplants, as programs can now wait longer for better quality and matched organs, while patients are supported by VADs. Currently, data on VAD utilization are not captured by CORR.

#### Lung transplantation

Like heart transplantation, there has been a progressive increase in the number of prevalent wait-listed lung transplant candidates over the last decade. At total of 315 patients were waiting for a lung transplant at the end of 2012. This represents a 93% increase since 2001. Unfortunately, the wait-list mortality rate of lung transplant candidates remains high, ranging from 18 to 24% over the last decade (Additional file 1: Figure S15). With the increasing number of patients waiting for a lung transplant, there has also been an increase in the number of lung transplant procedures. In 2012, a total of 191 lung transplants were performed, which is the most in a single year over the prior decade (Additional file 1: Table S4). The vast majority of these transplants were bilateral lung procedures (157 of 191 in 2012) and this pattern persisted over the last decade (Additional file 1: Table S5). The 1- and 5-year cumulative survival probabilities after lung transplantation range from 82.8 to 88.3% and 63.0 to 66.1%, depending on recipient age category (Additional file 1: Figure S16). Marked improvements in both short and long-term lung transplant survival have been observed from 2003 to 2012 (Additional file 1: Figure S17).

#### Kidney-pancreas and islet transplantation

The prevalent population of wait-listed kidney-pancreas transplant candidates has seen some fluctuations over the last decade but, in general, there has been approximately 100 to 130 patients awaiting transplantation at the end of most years (122 patients at the end of 2012). The wait-list mortality rate saw an unexplained increase from its baseline of 2 to 5% to 15% in 2010 and 2011. The most recent year showed a return to the baseline mortality rate of 2% (Additional file 1: Figure S18). This baseline reflects what has been historically observed in kidney transplant candidates. There were 77 kidney-pancreas transplants in 2012, which represents a 20% increase from 2003. Islet transplants have also seen a record year with 62 procedures in 2012, up from 35 in 2003 (Additional file 1: Table S6). The 1- and 5-year cumulative survival probabilities after kidney-pancreas transplantation range from 90.3 to 97.7% and 86.7 to 88.8%, depending on recipient age category (Additional file 1: Figure S19). The survival of kidney-pancreas transplants has not shown consistent trends over time but this may at least partially reflect the small numbers of transplants especially in the earlier era (Additional file 1: Figure S20).

#### Intestinal and multi-visceral transplantation

Intestinal and multi-visceral transplants are generally uncommon with a total of 34 procedures performed from 2003 to 2012. Slightly over half of these transplants (i.e., 18 of 34) have been performed in children under the age of 18 years. Most intestinal transplants have been performed in conjunction with a liver (11 from 2003 to 2012). Multi-visceral transplants represent a total of 11 procedures during the same time period (Additional file 1: Table S7).

### Access to kidney transplantation feasibility project

In June 2010, the CORR Board, in partnership with CIHI, initiated data collection on all newly referred ESKD patients at 16 of 18 adult kidney transplant centres across Canada. This project is called the “Access to Kidney Transplantation Feasibility Project” and is supported by an unrestricted grant from Roche Canada. The objectives are to gain insights into the types of ESKD patients being referred to kidney transplant centres across Canada, assess the timeliness and effectiveness of wait-listing practices, and track the outcomes of patients wait-listed for deceased donor kidney transplantation. Patients have been recruited for a 3-year period (until May 2013) and 2-years of additional follow-up are ongoing. Over 7,000 patients have been recruited into this study (Table 2). Data on wait-listed kidney transplant candidates are collected in other national registries (e.g., the U.S. Scientific Registry of Transplant Recipients) but this project is the first to collect information from the time of referral. As the study comes to completion, a number of analyses are planned with the goal of publishing the findings in peer-reviewed journals.

**Table 2:** **Baseline characteristics of patient referred for kidney transplantation**

**Patient characteristics at referral**	**Summary measure**
Total sample size	7,409
Age (mean, years)	52.3
Age ≥ 65 years (%)	19.2
Sex (%)	
Female	36.9
Male	63.1
Race (%)	
Caucasian	52.2
Other	28.2
Unknown	19.5
Valid HCN (%)	99.6
Valid postal code (%)	98.6
On dialysis (%)	60.5
Not on dialysis, SCr (mean, μmol/L)	383.4
Not on dialysis, SCr missing (%)	7.9

### Transplantation research using CORR

When a PubMed search of the terms “Canadian Organ Replacement Register” or “Canadian Organ Replacement Registry” and “transplantation” or “transplant” (as a MeSH or key word) was performed on 1 Sep 2014, a total of 49 citations were retrieved. Among these citations, 34 were transplant-related studies/reports using CORR data. Eight additional papers (seven original articles and one commentary) were retrieved from the lead author’s files, resulting in a total of 42 publications (see the full list of citations in the Additional file 1). Over half of the papers (n = 25) related to kidney transplantation, while a smaller number of publications focused on extra-renal transplants (n = 9), deceased organ donation (n = 1), and general reports on ESKD and/or transplantation in Canada (n = 7).

Along with studies that highlighted trends in transplantation in Canada, the availability of registries from other countries have allowed for international comparative analyses. For example, the mortality of kidney transplant recipients in Canadian vs. U.S. patients was investigated using data from CORR and the U.S. Scientific Registry of Transplant Recipients [5]. This study revealed that U.S. patients were 35% more likely to die after kidney transplantation than their Canadian counterparts. This mortality difference was further accentuated the longer patients were on dialysis before transplant and the longer their follow-up after transplant. Similarly, an analysis comparing the graft survival of kidney transplants in Commonwealth countries (i.e., Canada, Australia, and the U.K.) vs. the U.S. showed that the former had comparable 5- and 10-year graft survival rates, whereas the U.S. rate was significantly lower at both time points [6]. It was hypothesized that this observed difference in graft survival is at least partly driven by U.S. Medicare’s limited coverage of immunosuppressive medications.

Other areas of transplant research using CORR include an evaluation of centre effects in kidney transplantation [7], the incidence of cancer and ESKD among heart and liver transplant recipients [8-10], patient survival after kidney allograft failure [11-13], pediatric kidney transplant outcomes [14-17], access to kidney transplantation [18-20], and an assessment of misattributed paternity in living kidney donors [21]. These studies highlight the potential power of a national transplant registry in describing the epidemiology of solid organ transplantation (and related conditions), testing research hypotheses, and supporting the development of sound health policy. With improvements in data capture, completeness, and quality (as well as stronger engagement of key stakeholders), CORR has the potential to become an indispensible resource for patients, clinicians, researchers, and policy-makers, similar to other renal and transplant registries throughout the world (e.g., United States Renal Data System, Australia and New Zealand Dialysis and Transplant Registry).

A description of the CORR data request process can be found here (Additional file 2).

### Future directions

In the near future, CORR will be changing from paper-based data submissions to an entirely electronic-based submission strategy. The goal is to transition to a paperless approach starting with the 2015 data submission cycle. For centres that cannot use the standardized electronic submission specifications geared towards existing databases (such as the one used by the Ontario Renal Network), a web-based data entry solution that will allow data capture similar to the current data reporting forms is being developed. Comprehensive communication and training strategies are also concurrently being developed. Electronic data submissions will improve the efficiency, accuracy, and completeness of data collection, as well as increase data privacy and security.

Another important issue for CORR will be its ongoing relationship with Canadian Blood Services and their Canadian Transplant Registry (CTR). As the CTR evolves to become the main source for national data on organ donation and transplantation, CORR will continue to assess and re-evaluate the scope of its data holdings and develop appropriate strategies to continue to serve its stakeholders. One possibility will be to exclusively focus the registry’s efforts on kidney disease/transplantation and potentially expand its data holdings to include important clinical events (e.g., acute rejection episodes), immunosuppressive drug regimens, and transplant tourism. An ongoing interaction between the CTR and CORR is essential to ensure that the full journey of a kidney patient (which commonly includes kidney transplantation) is well represented in the data holdings of both registries. At this time, CORR/CIHI is working with Canadian Blood Services to support and engage in the evolution of the CTR (including a comprehensive review and standardization of data elements to be captured in the CTR) and progress on this issue is being followed with great interest by the renal and transplant communities.

## Conclusion

CORR is an important source of information on the status of solid organ transplantation in Canada. It has and continues to provide its stakeholders with a national perspective on organ donation activity, the number/characteristics of organ donors and transplant candidates, and the long-term outcomes of solid organ transplant recipients. It is used for epidemiologic and health services research that contributes to improving clinical practice and informing the development of health policy in transplantation. CORR is an example of the power of registries for the surveillance and advancement of patient care in complex health care arenas such as transplantation. The importance of registry data in monitoring and improving care for Canadian solid organ transplant candidates/recipients cannot be over-stated.

## Additional files

Additional file 1:
**Supplemental Appendix.**
Additional file 2:
**Data Request Process.**

## Abbreviations

CIHI: Canadian Institute for Health Information
CORR: Canadian Organ Replacement Register
CTR: Canadian Transplant Registry
ESKD: End-stage kidney disease
DCD: Donation after cardio-circulatory death
NDD: Neurological determination of death
OPO: Organ procurement organization
PMP: Per million population
VAD: Ventricular assist device
