# Supplementary material for: Organ donation and transplantation in Canada: insights from the Canadian Organ Replacement Register
Source: Can J Kidney Health Dis. 2014 Dec 9;1:31. doi: 10.1186/s40697-014-0031-8 (PMC4349751; doi:10.1186/s40697-014-0031-8)
Supplement: Additional file 1: — Supplemental Appendix. [file 40697_2014_31_MOESM1_ESM.ppt]

## Slide 1
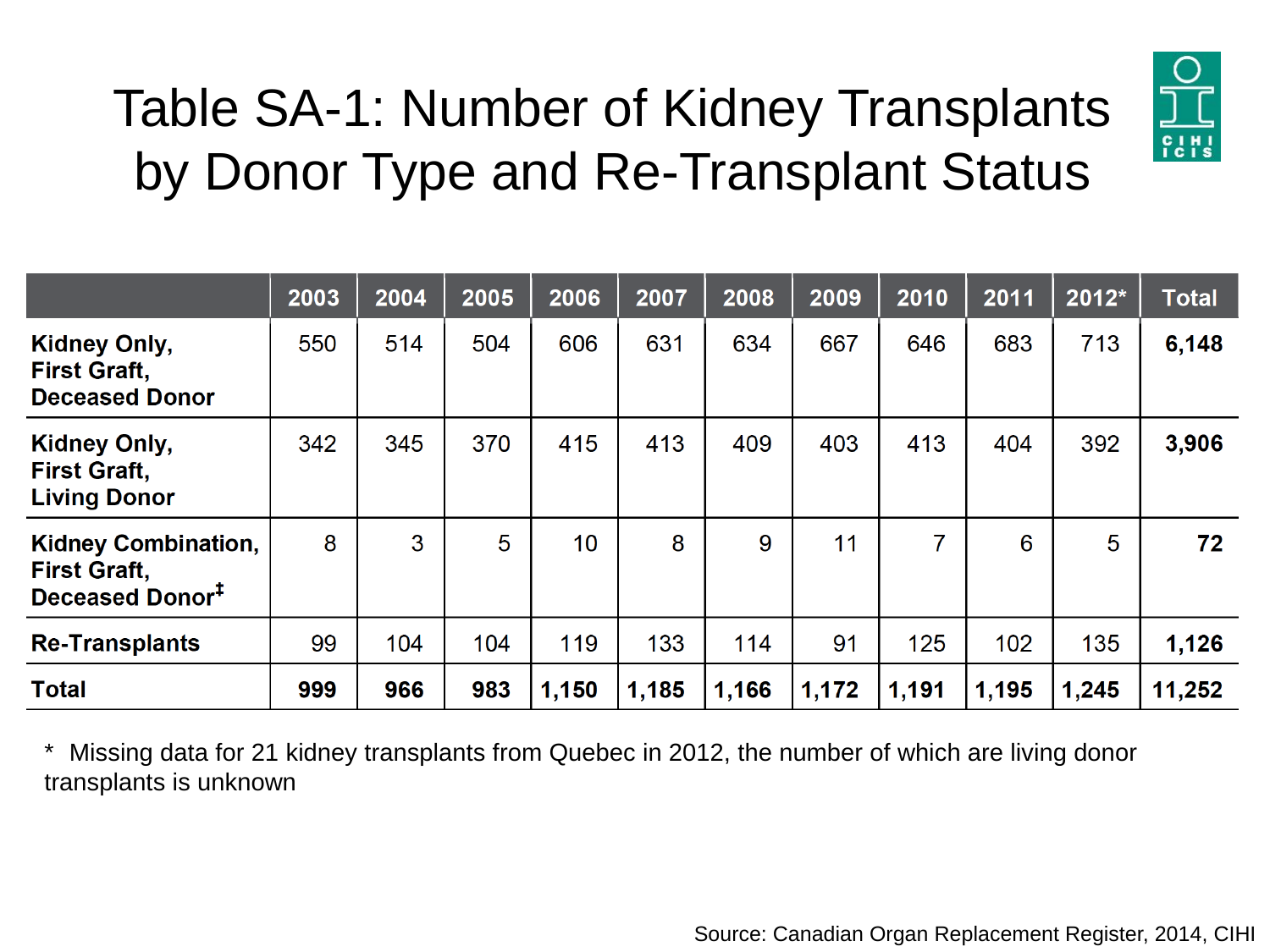

# Table SA-1: Number of Kidney Transplants by Donor Type and Re-Transplant Status
*	Missing data for 21 kidney transplants from Quebec in 2012, the number of which are living donor 	transplants is unknown
Source: Canadian Organ Replacement Register, 2014, CIHI

## Slide 2
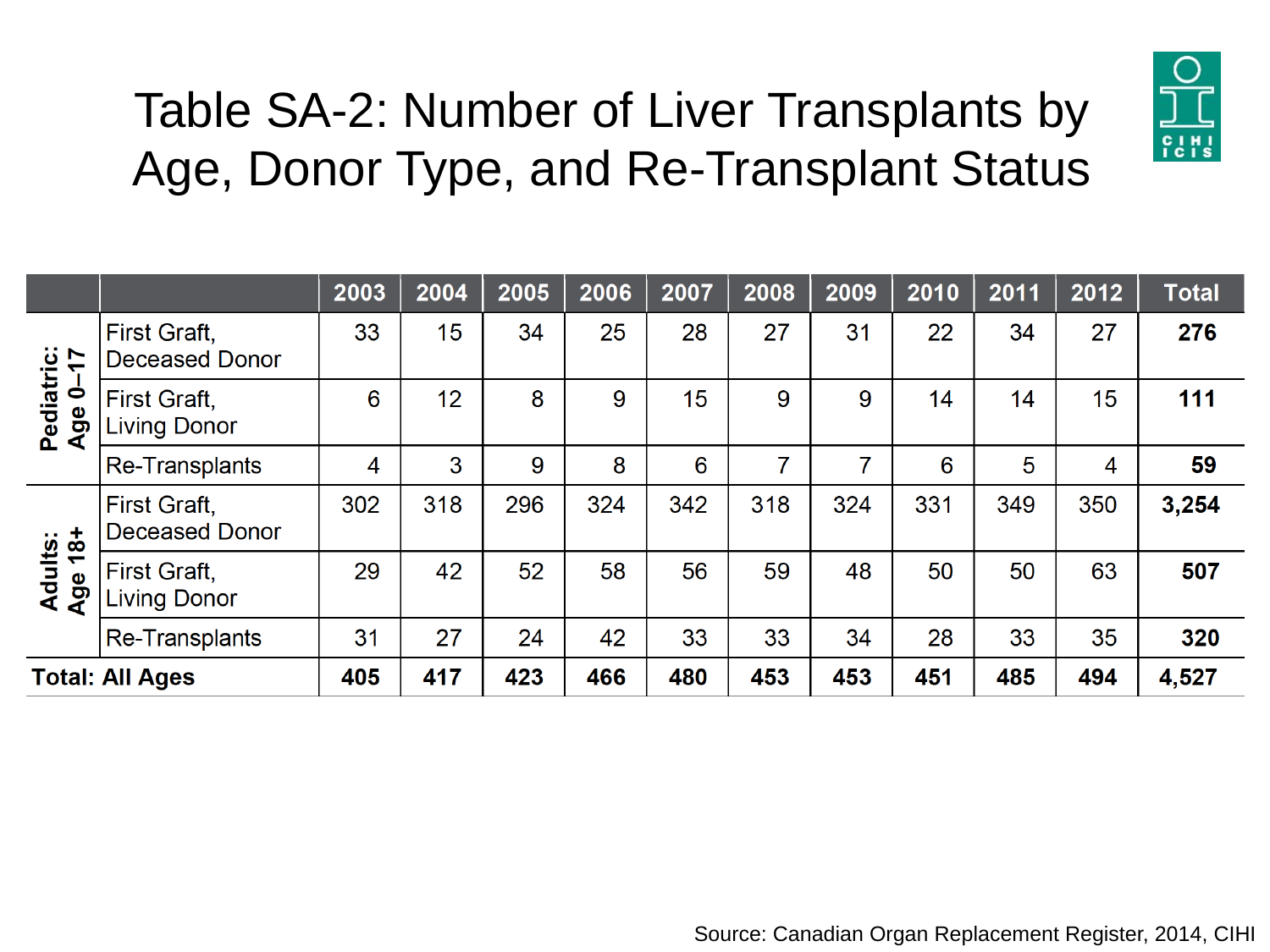

# Table SA-2: Number of Liver Transplants by Age, Donor Type, and Re-Transplant Status
Source: Canadian Organ Replacement Register, 2014, CIHI

## Slide 3
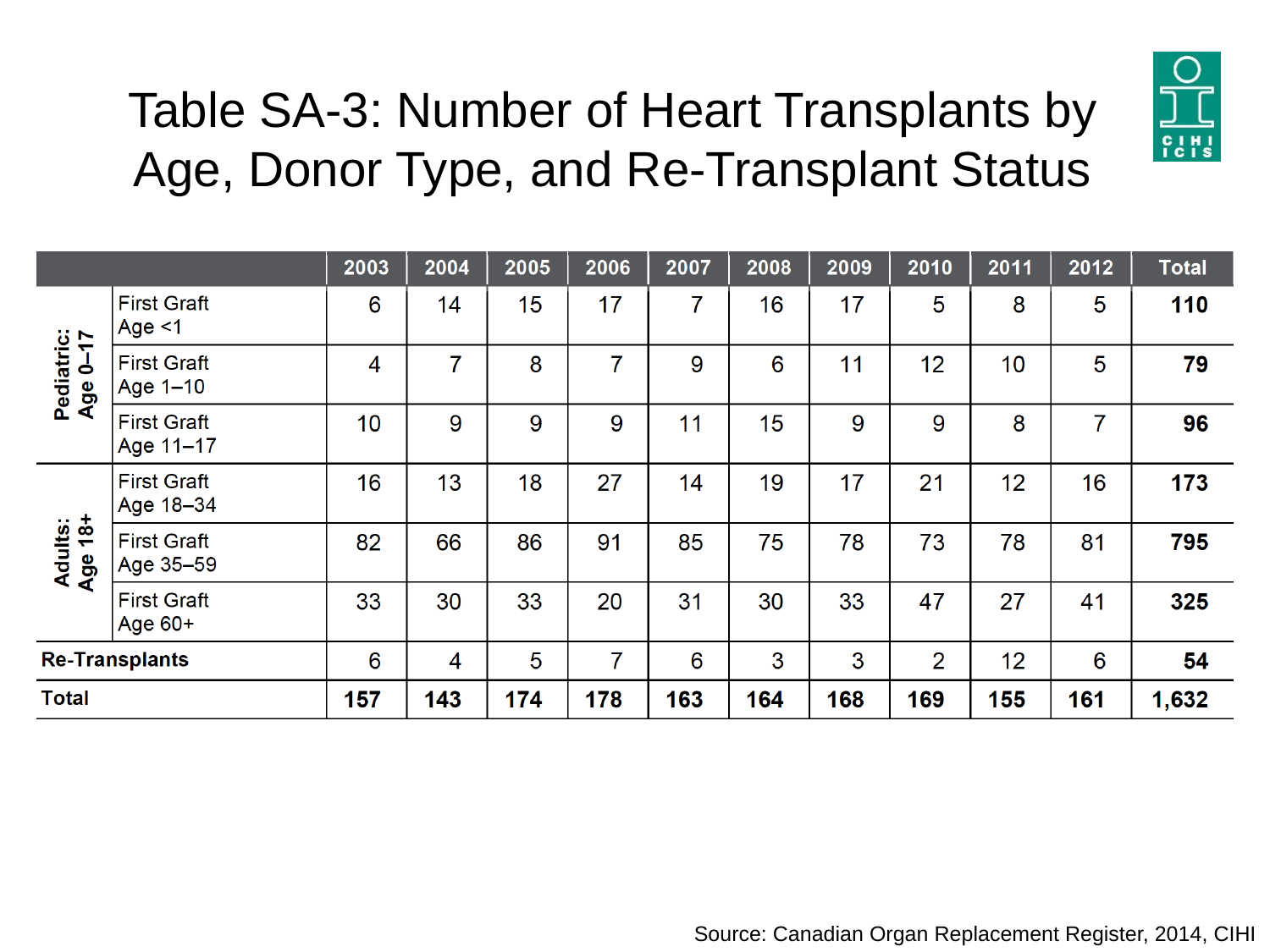

# Table SA-3: Number of Heart Transplants by Age, Donor Type, and Re-Transplant Status
Source: Canadian Organ Replacement Register, 2014, CIHI

## Slide 4
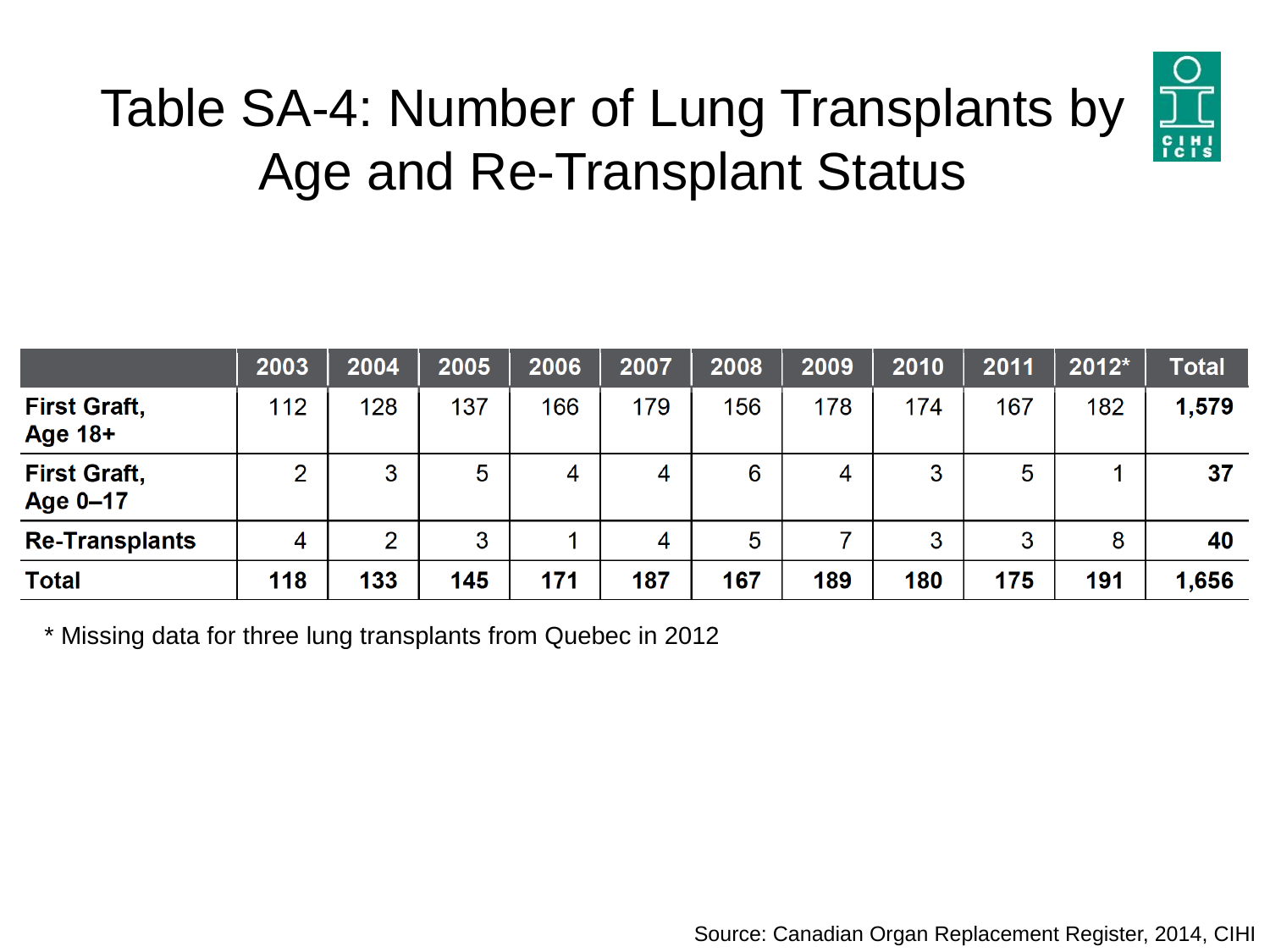

# Table SA-4: Number of Lung Transplants by Age and Re-Transplant Status
* Missing data for three lung transplants from Quebec in 2012
Source: Canadian Organ Replacement Register, 2014, CIHI

## Slide 5
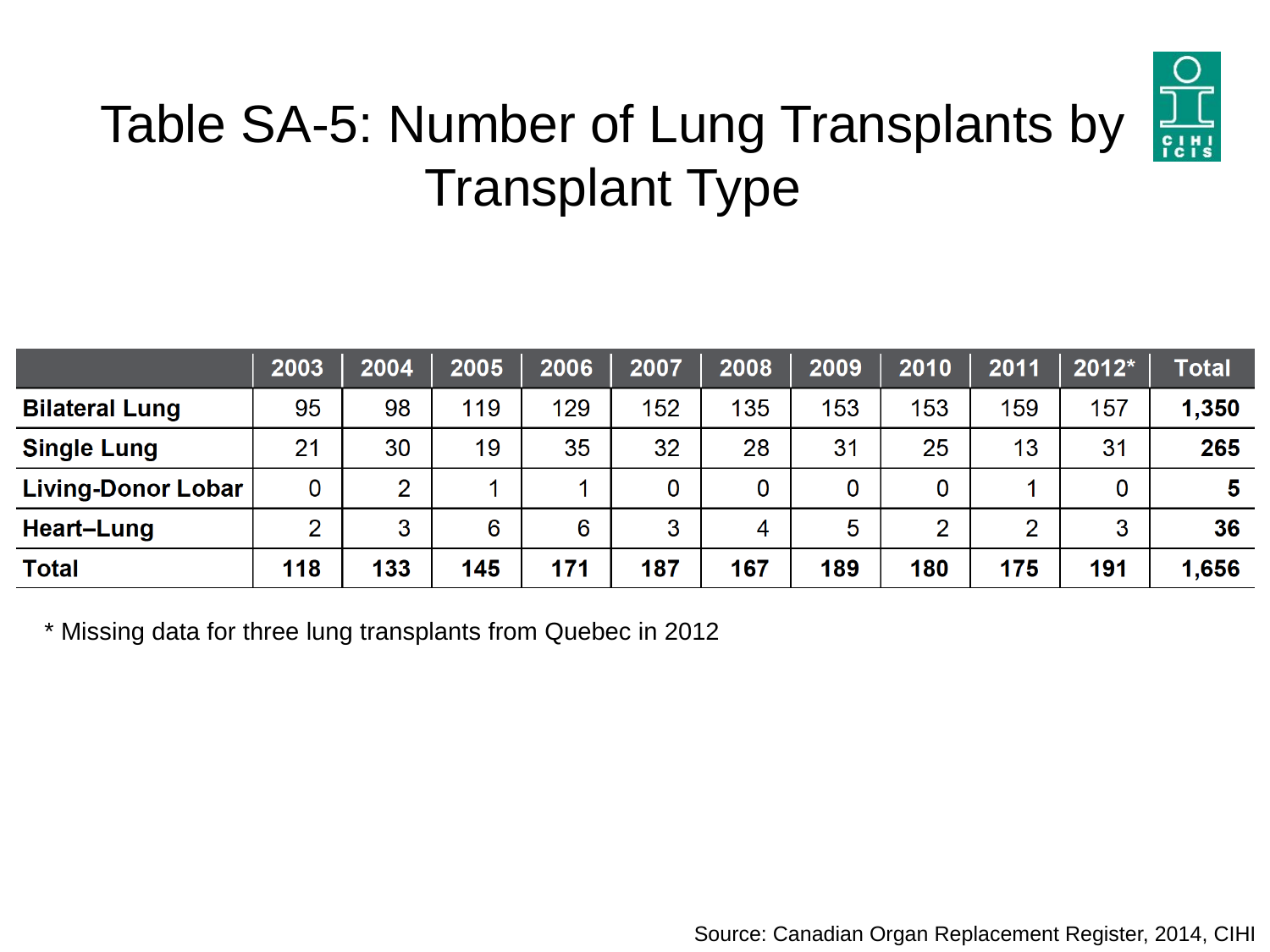

# Table SA-5: Number of Lung Transplants by Transplant Type
* Missing data for three lung transplants from Quebec in 2012
Source: Canadian Organ Replacement Register, 2014, CIHI

## Slide 6
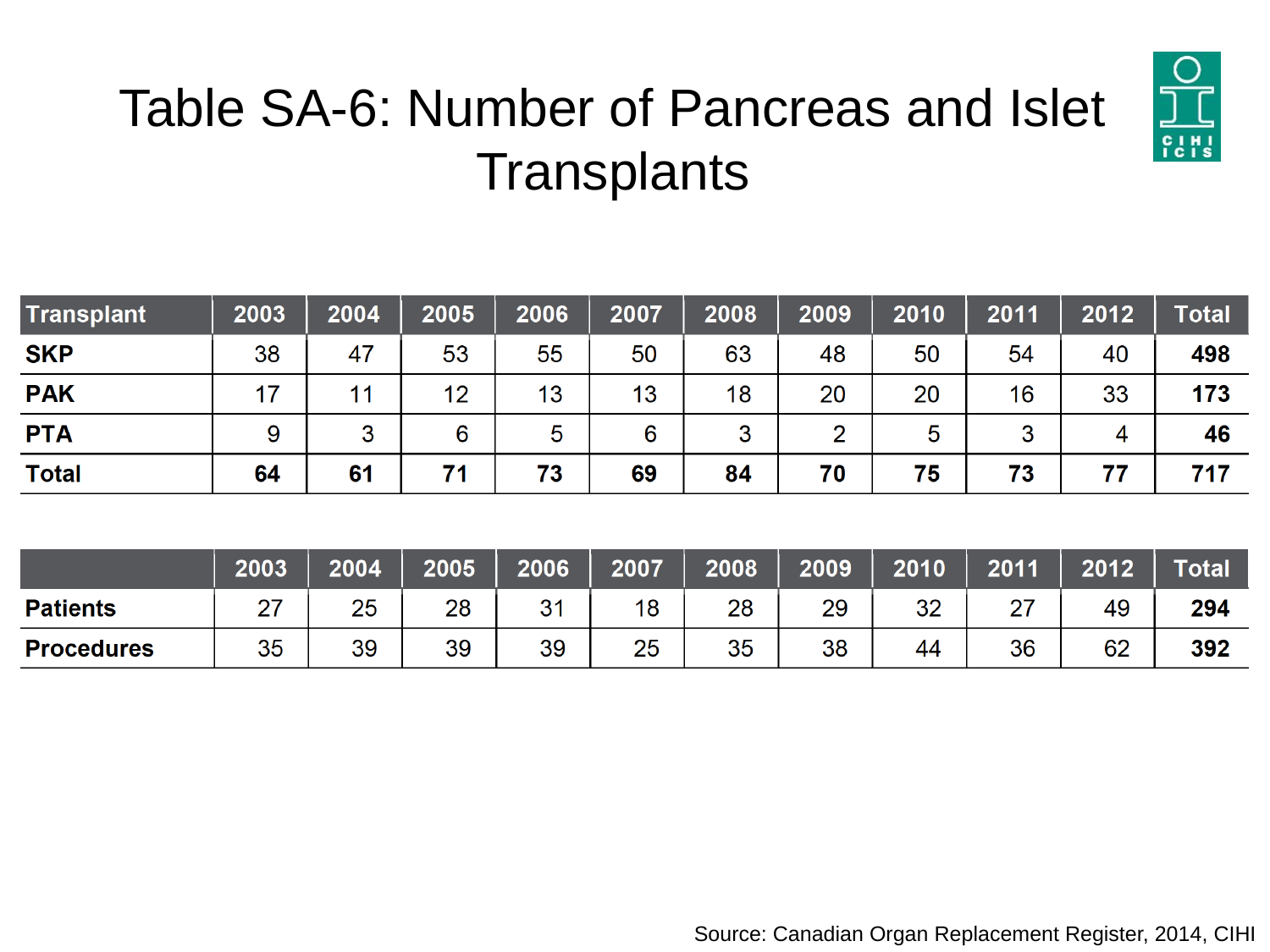

# Table SA-6: Number of Pancreas and Islet Transplants
Source: Canadian Organ Replacement Register, 2014, CIHI

## Slide 7
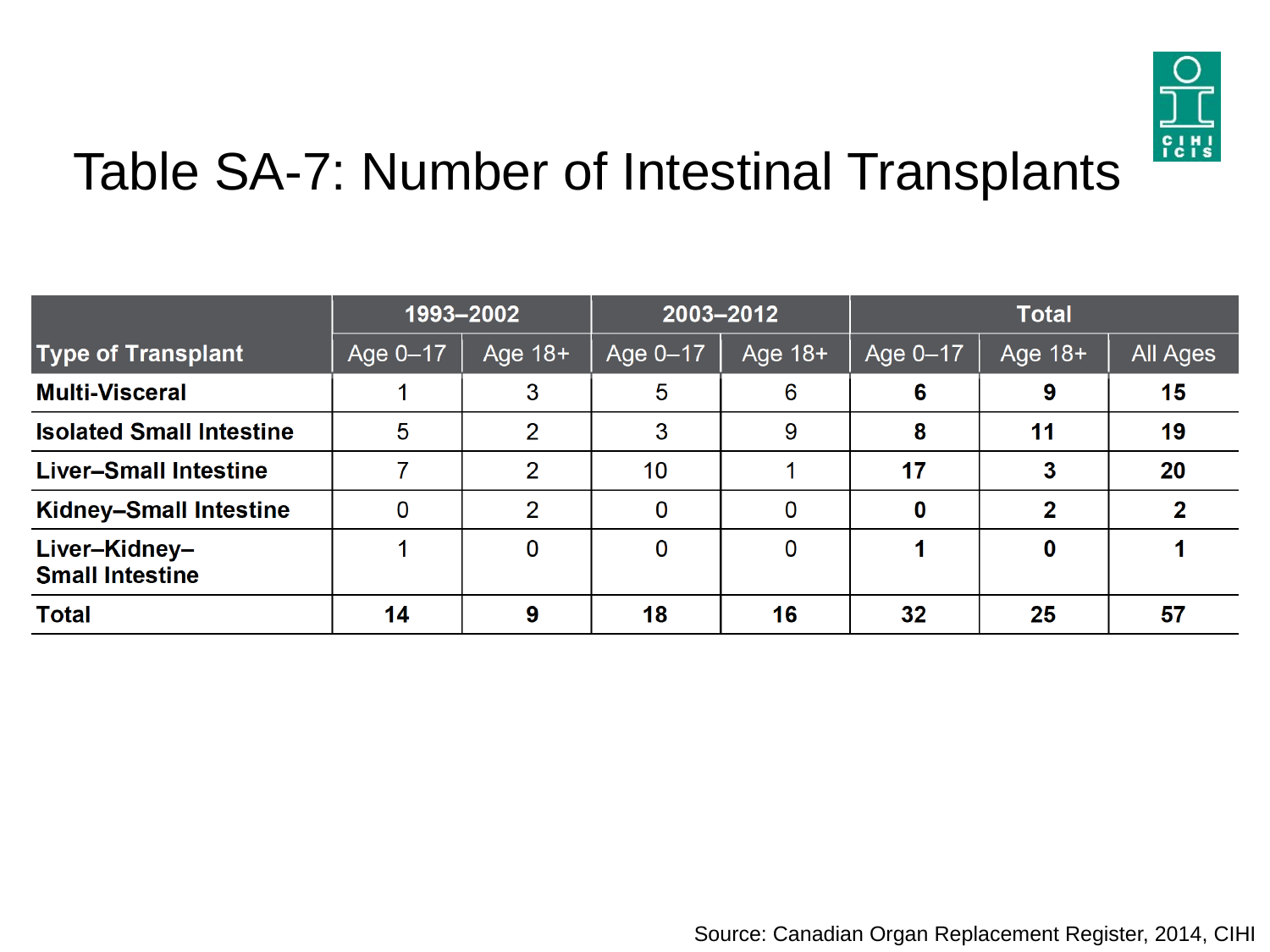

# Table SA-7: Number of Intestinal Transplants
Source: Canadian Organ Replacement Register, 2014, CIHI

## Slide 8
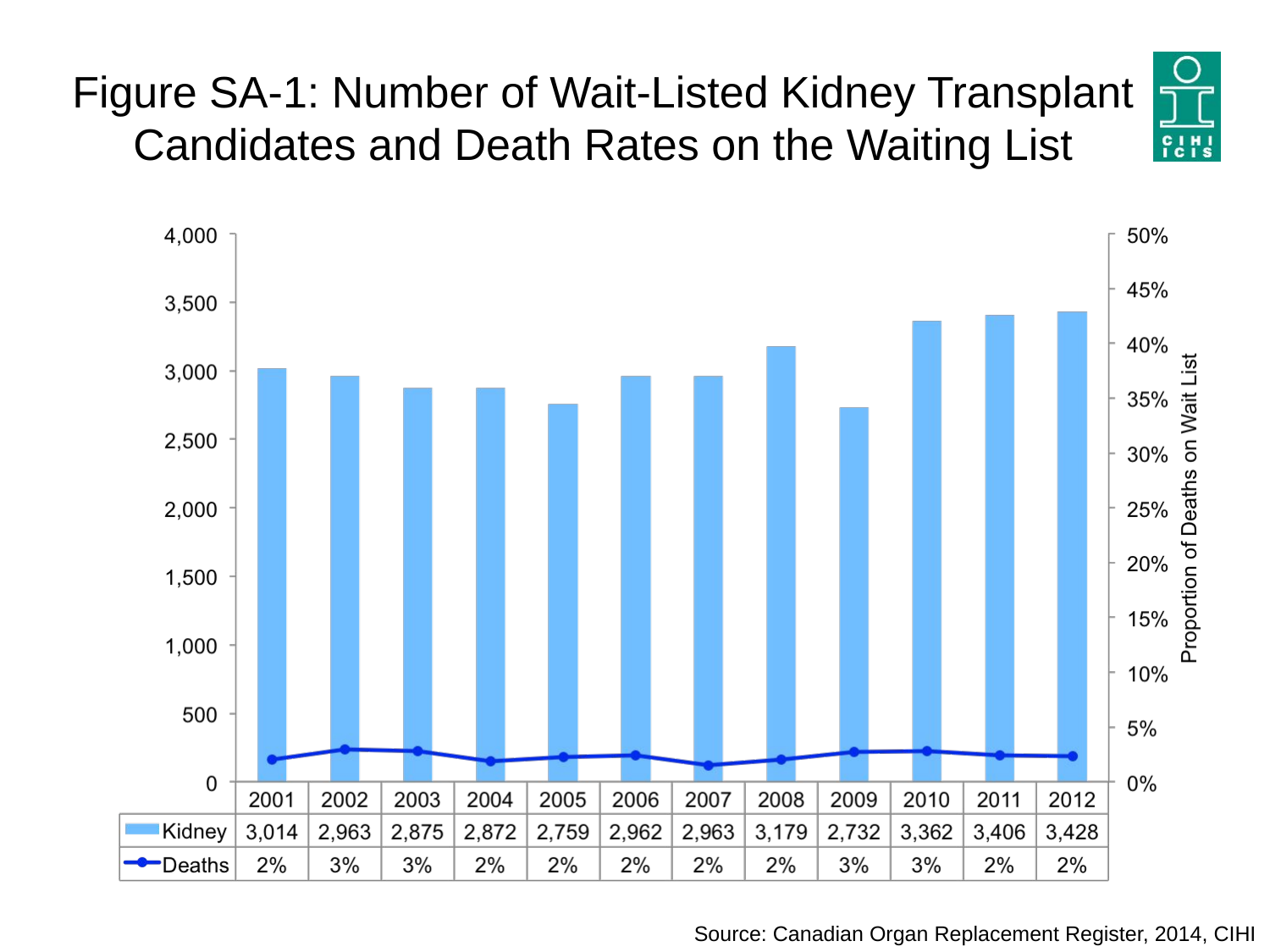

# Figure SA-1: Number of Wait-Listed Kidney Transplant Candidates and Death Rates on the Waiting List
Source: Canadian Organ Replacement Register, 2014, CIHI

## Slide 9
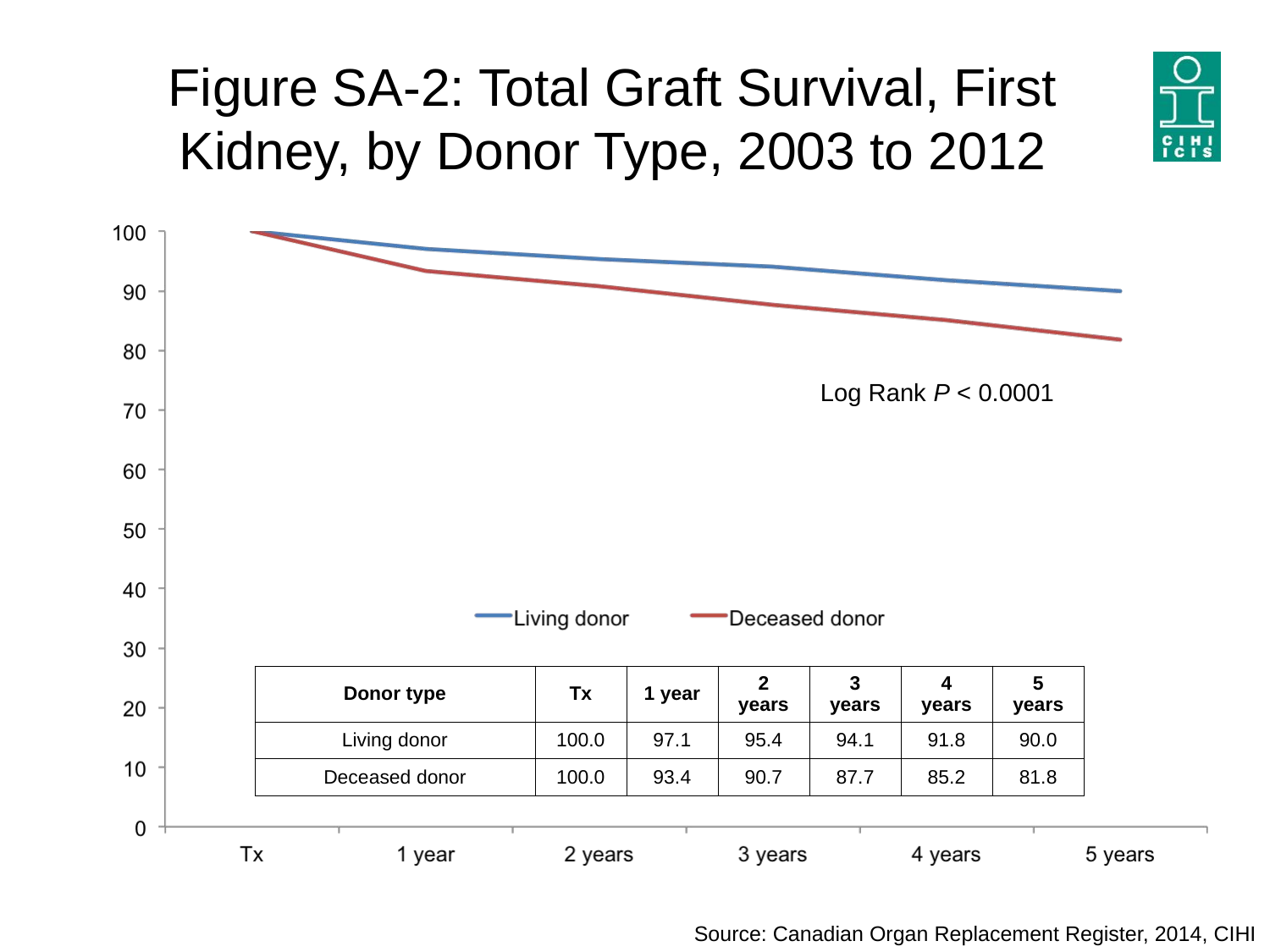

# Figure SA-2: Total Graft Survival, First Kidney, by Donor Type, 2003 to 2012
Log Rank P < 0.0001
| Donor type | Tx | 1 year | 2 years | 3 years | 4 years | 5 years |
| --- | --- | --- | --- | --- | --- | --- |
| Living donor | 100.0 | 97.1 | 95.4 | 94.1 | 91.8 | 90.0 |
| Deceased donor | 100.0 | 93.4 | 90.7 | 87.7 | 85.2 | 81.8 |
Source: Canadian Organ Replacement Register, 2014, CIHI

## Slide 10
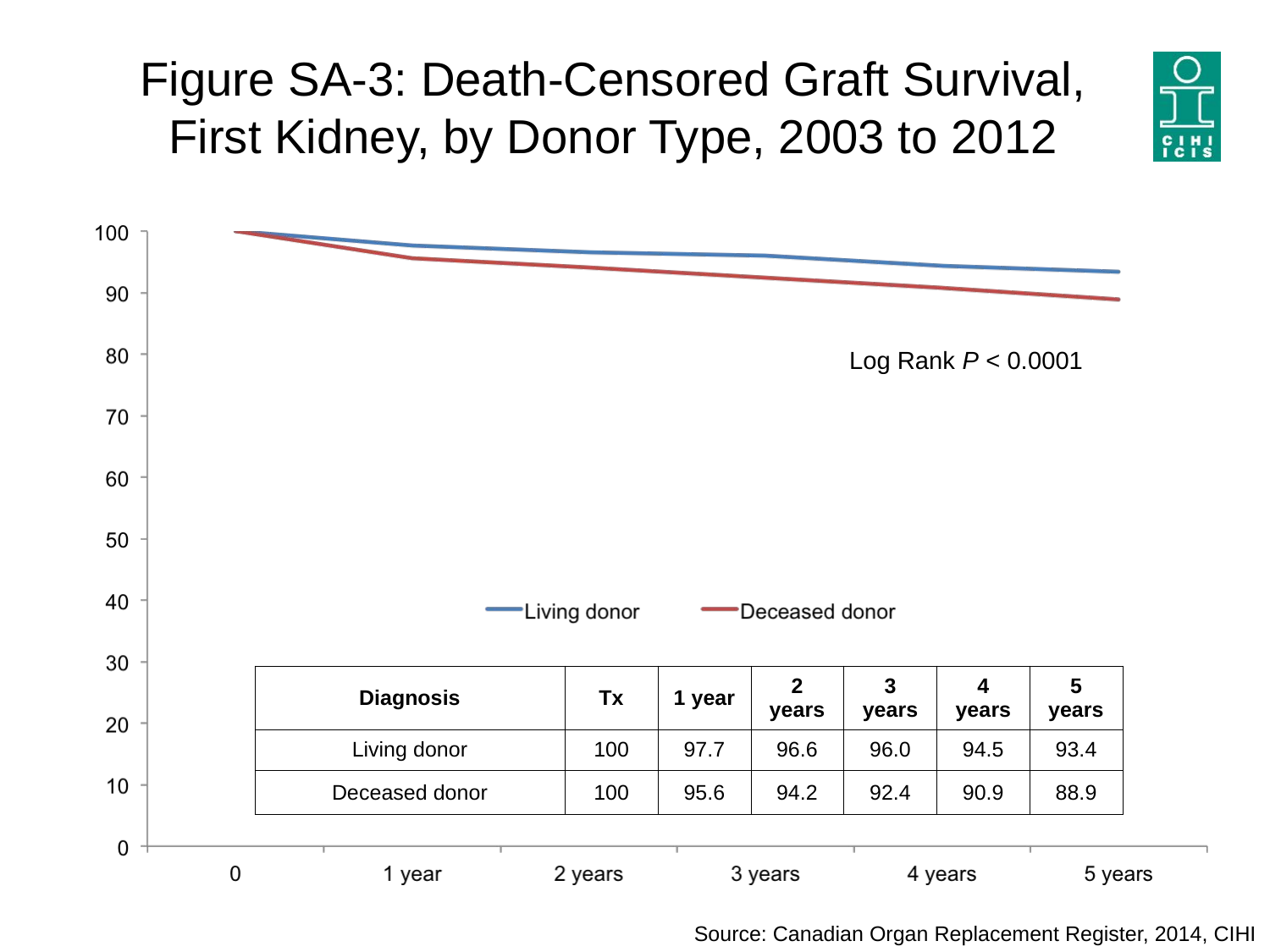

# Figure SA-3: Death-Censored Graft Survival, First Kidney, by Donor Type, 2003 to 2012
Log Rank P < 0.0001
| Diagnosis | Tx | 1 year | 2 years | 3 years | 4 years | 5 years |
| --- | --- | --- | --- | --- | --- | --- |
| Living donor | 100 | 97.7 | 96.6 | 96.0 | 94.5 | 93.4 |
| Deceased donor | 100 | 95.6 | 94.2 | 92.4 | 90.9 | 88.9 |
Source: Canadian Organ Replacement Register, 2014, CIHI

## Slide 11
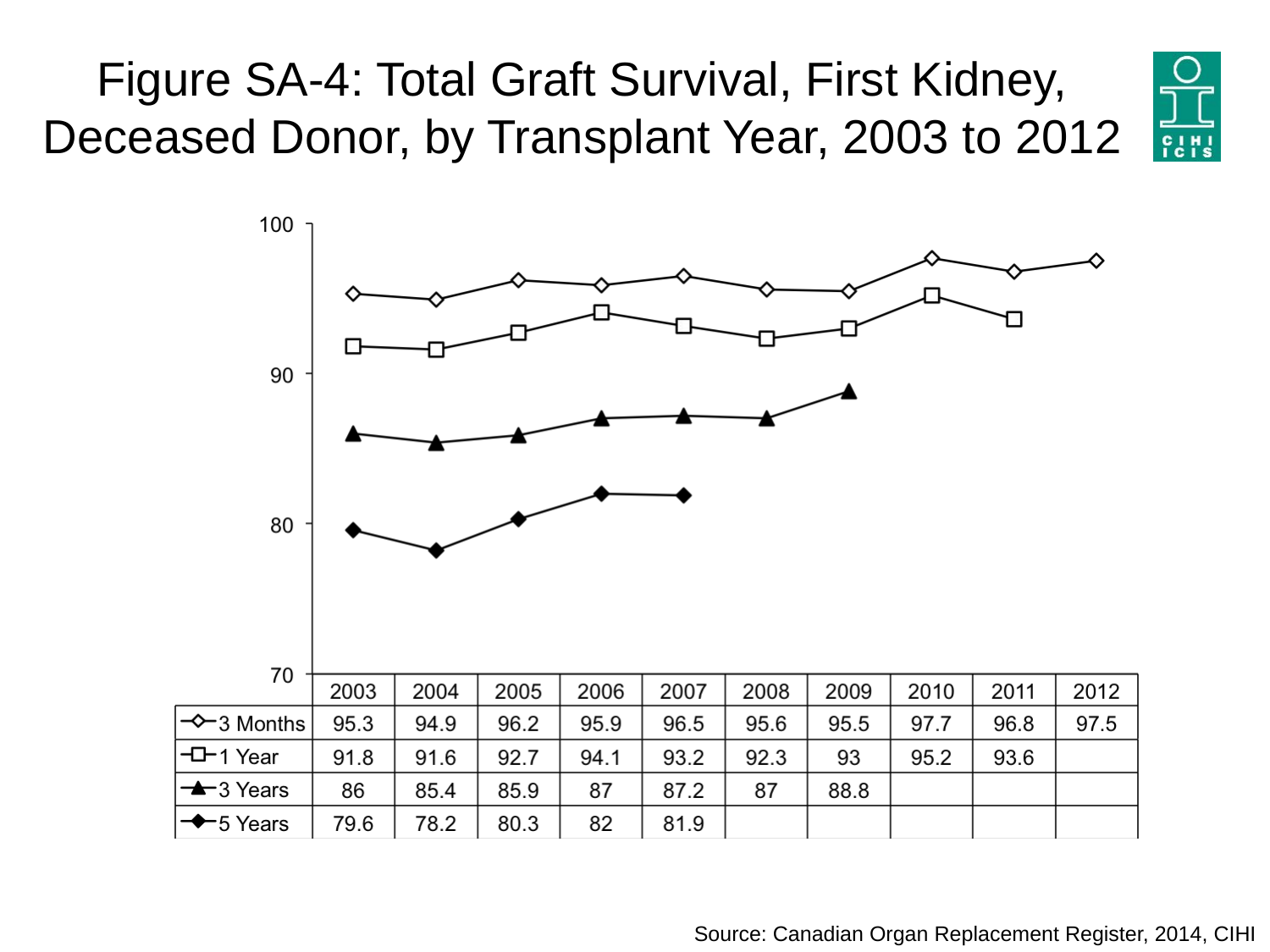

# Figure SA-4: Total Graft Survival, First Kidney, Deceased Donor, by Transplant Year, 2003 to 2012
Source: Canadian Organ Replacement Register, 2014, CIHI

## Slide 12
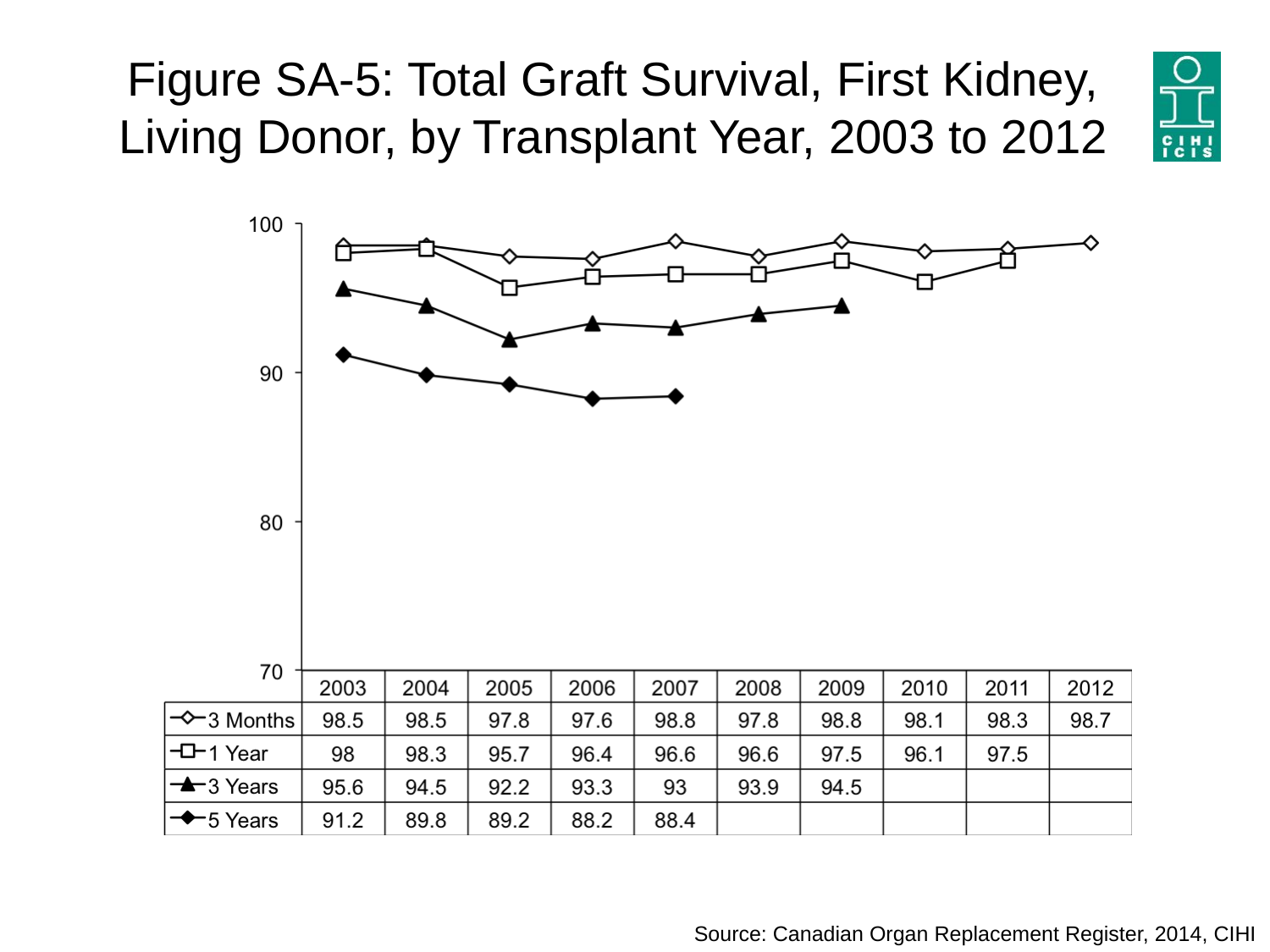

# Figure SA-5: Total Graft Survival, First Kidney, Living Donor, by Transplant Year, 2003 to 2012
Source: Canadian Organ Replacement Register, 2014, CIHI

## Slide 13
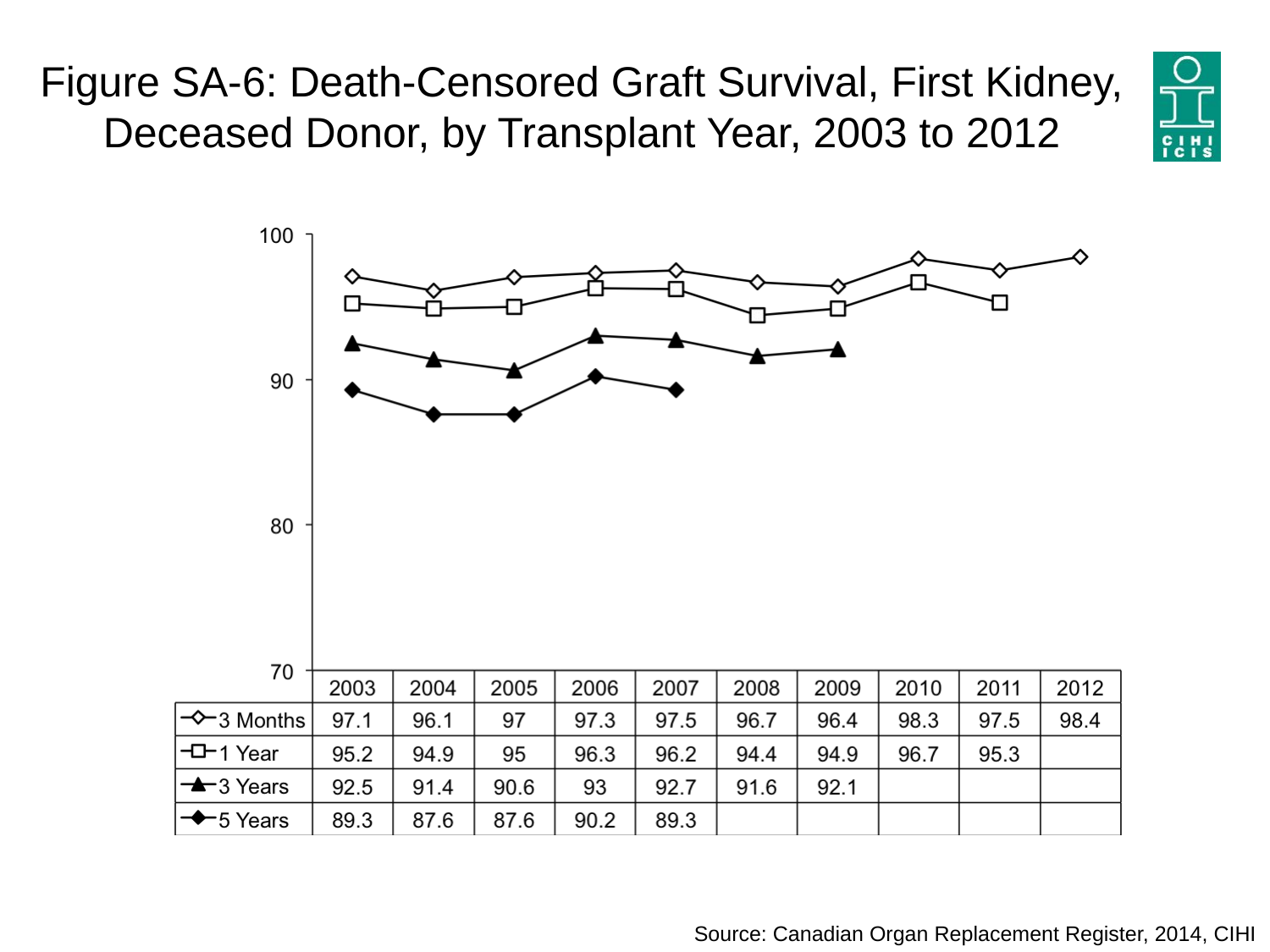

# Figure SA-6: Death-Censored Graft Survival, First Kidney, Deceased Donor, by Transplant Year, 2003 to 2012
Source: Canadian Organ Replacement Register, 2014, CIHI

## Slide 14
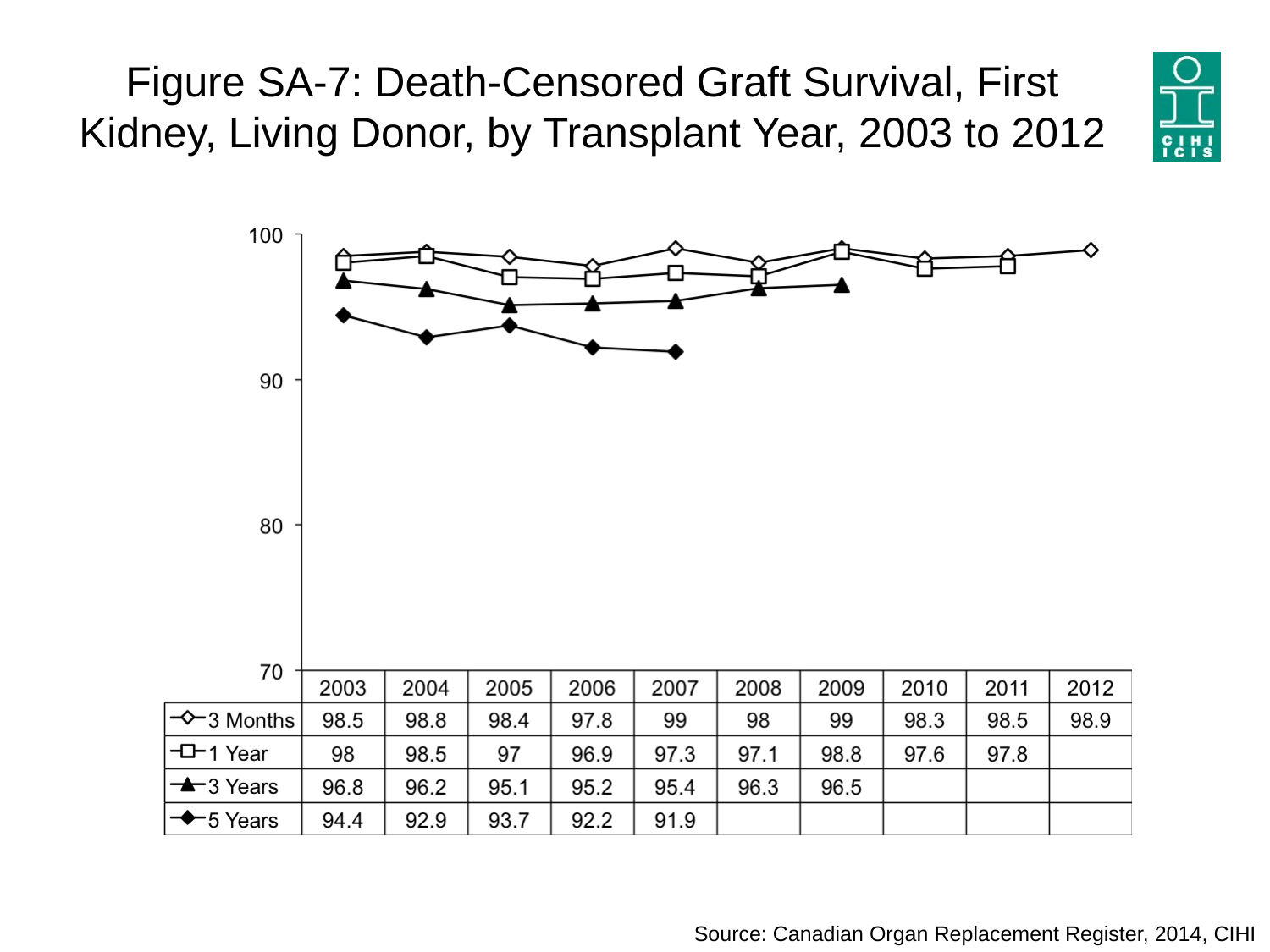

# Figure SA-7: Death-Censored Graft Survival, First Kidney, Living Donor, by Transplant Year, 2003 to 2012
Source: Canadian Organ Replacement Register, 2014, CIHI

## Slide 15
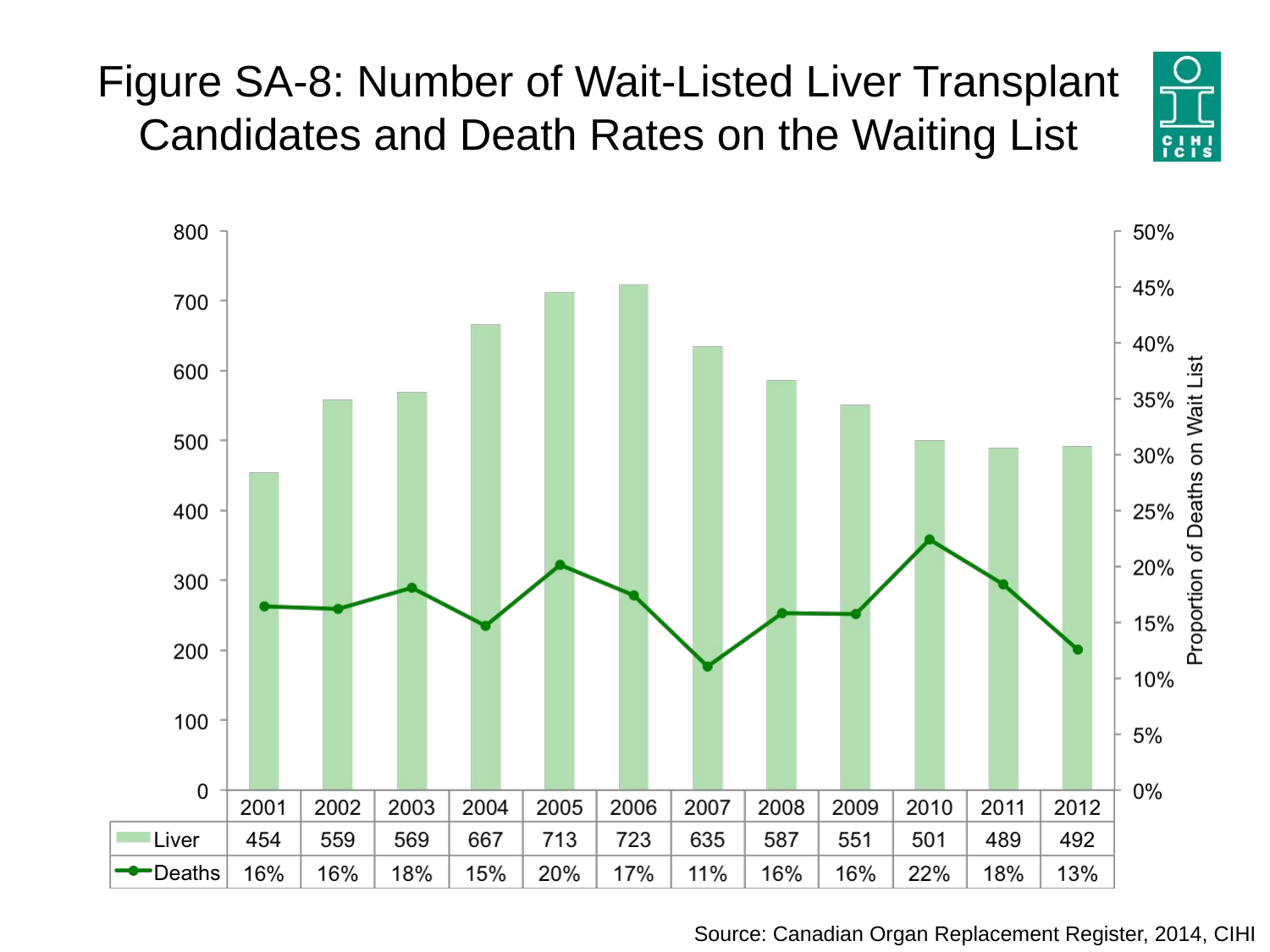

# Figure SA-8: Number of Wait-Listed Liver Transplant Candidates and Death Rates on the Waiting List
Source: Canadian Organ Replacement Register, 2014, CIHI

## Slide 16
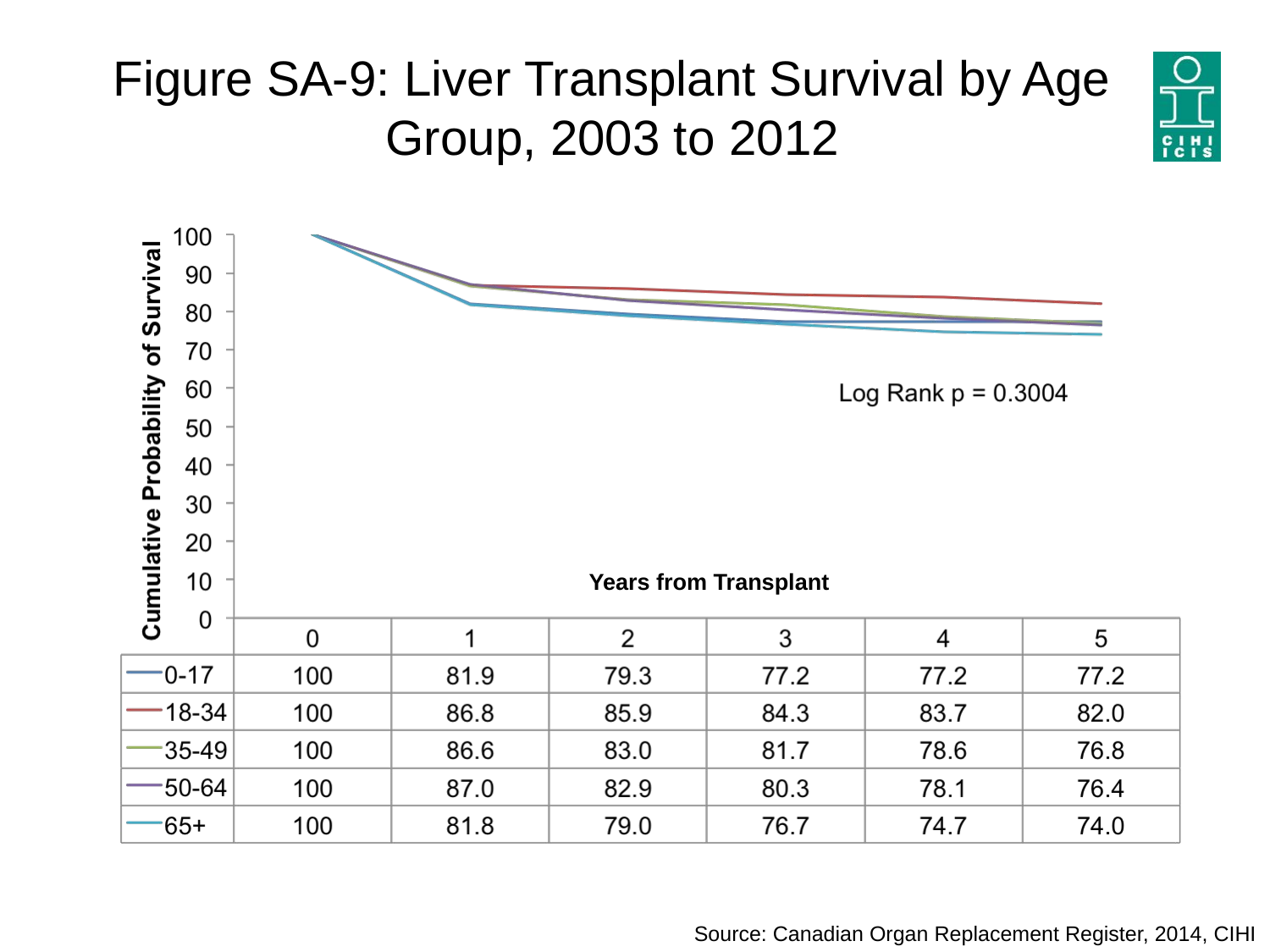

# Figure SA-9: Liver Transplant Survival by Age Group, 2003 to 2012
Years from Transplant
Source: Canadian Organ Replacement Register, 2014, CIHI

## Slide 17
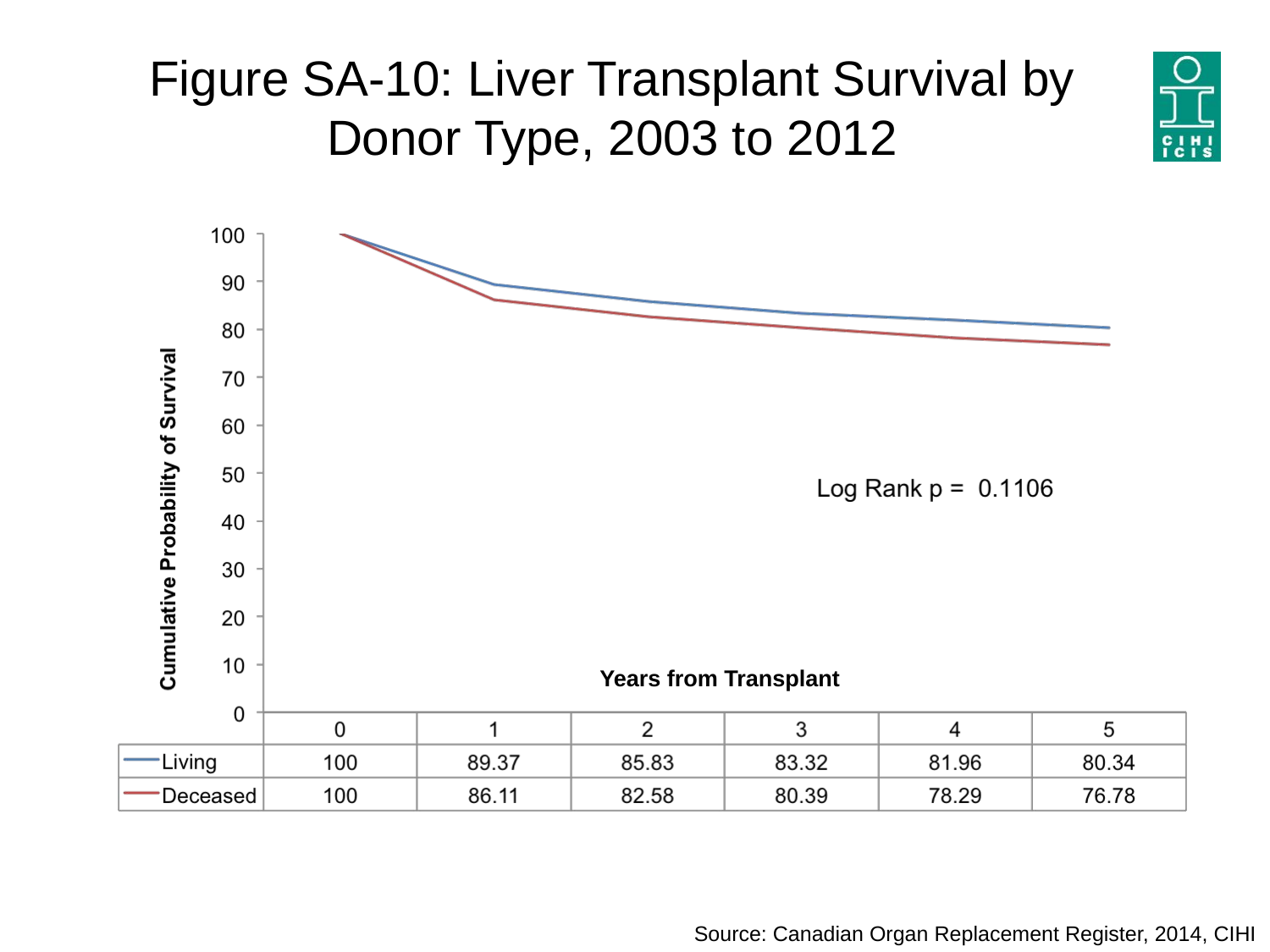

# Figure SA-10: Liver Transplant Survival by Donor Type, 2003 to 2012
Years from Transplant
Source: Canadian Organ Replacement Register, 2014, CIHI

## Slide 18
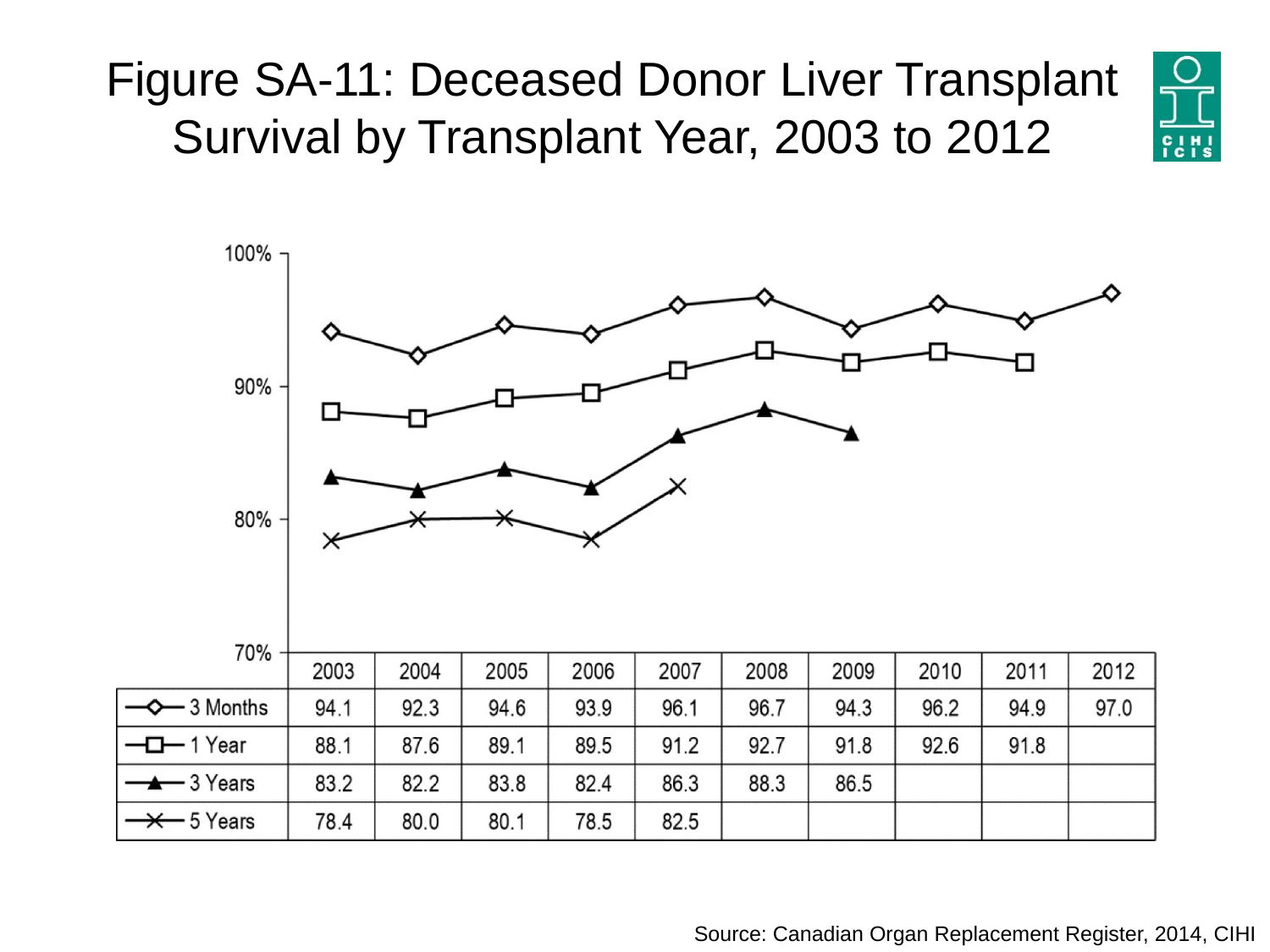

# Figure SA-11: Deceased Donor Liver Transplant Survival by Transplant Year, 2003 to 2012
Source: Canadian Organ Replacement Register, 2014, CIHI

## Slide 19
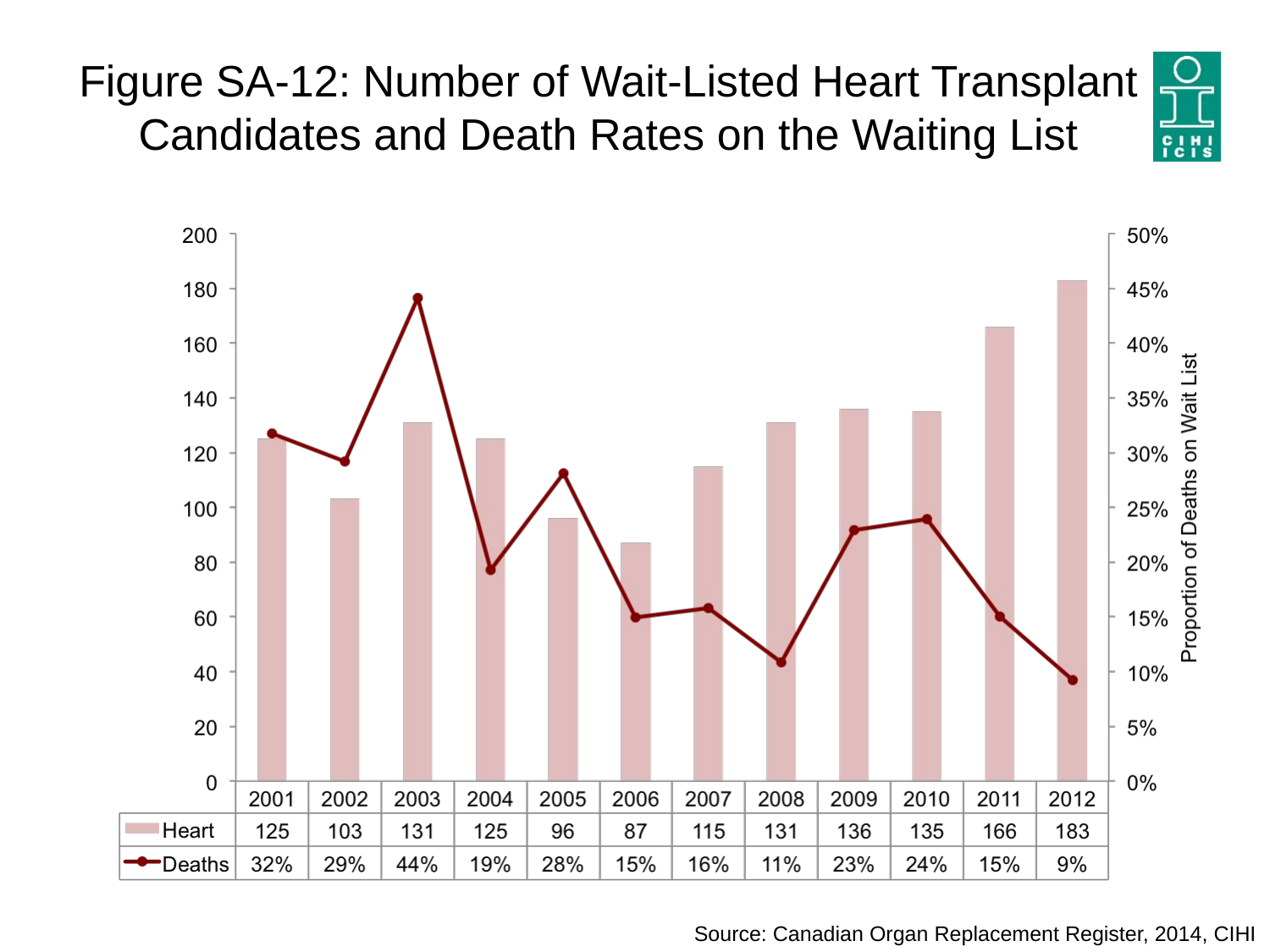

# Figure SA-12: Number of Wait-Listed Heart Transplant Candidates and Death Rates on the Waiting List
Source: Canadian Organ Replacement Register, 2014, CIHI

## Slide 20
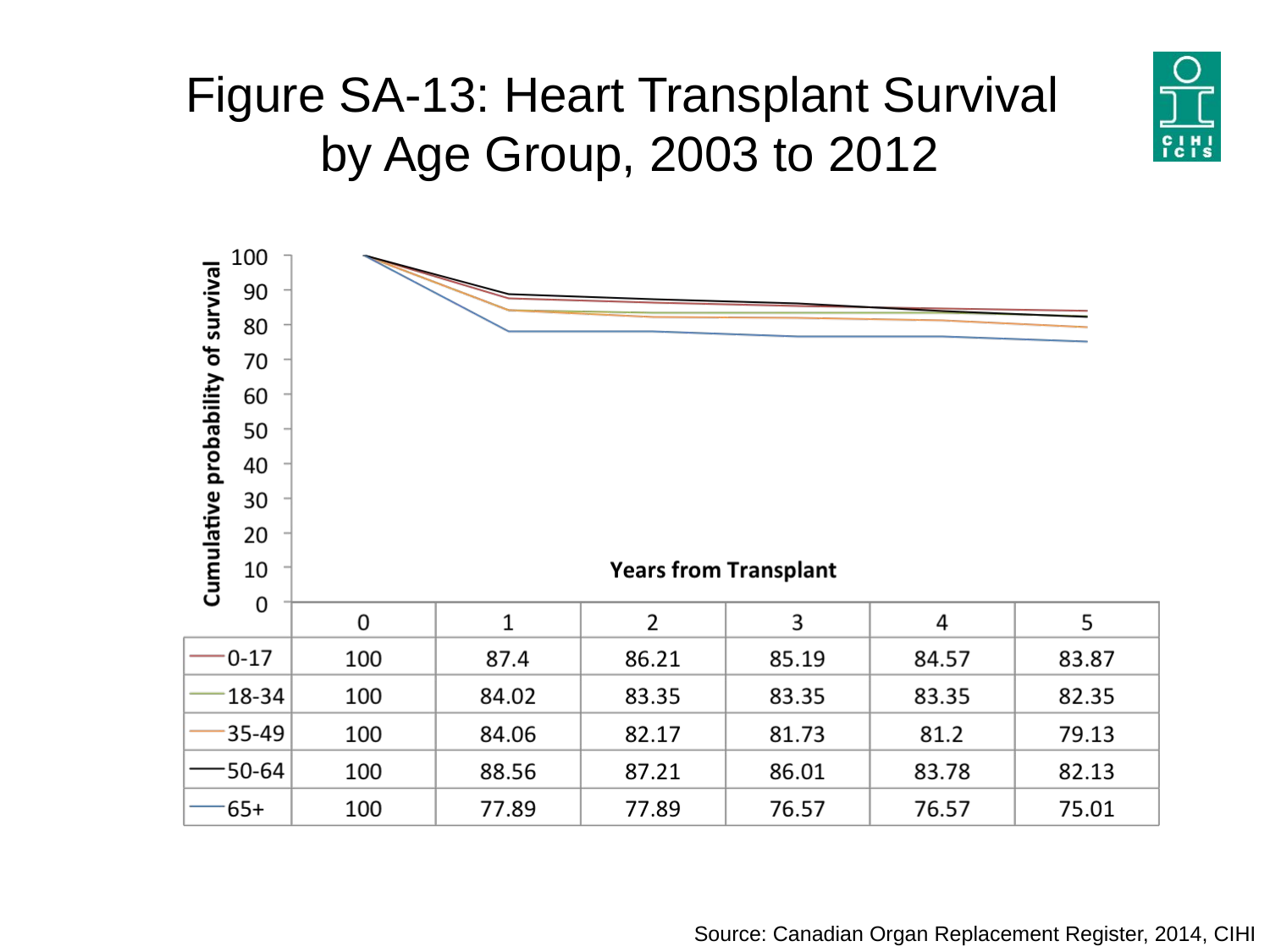

# Figure SA-13: Heart Transplant Survival by Age Group, 2003 to 2012
Source: Canadian Organ Replacement Register, 2014, CIHI

## Slide 21
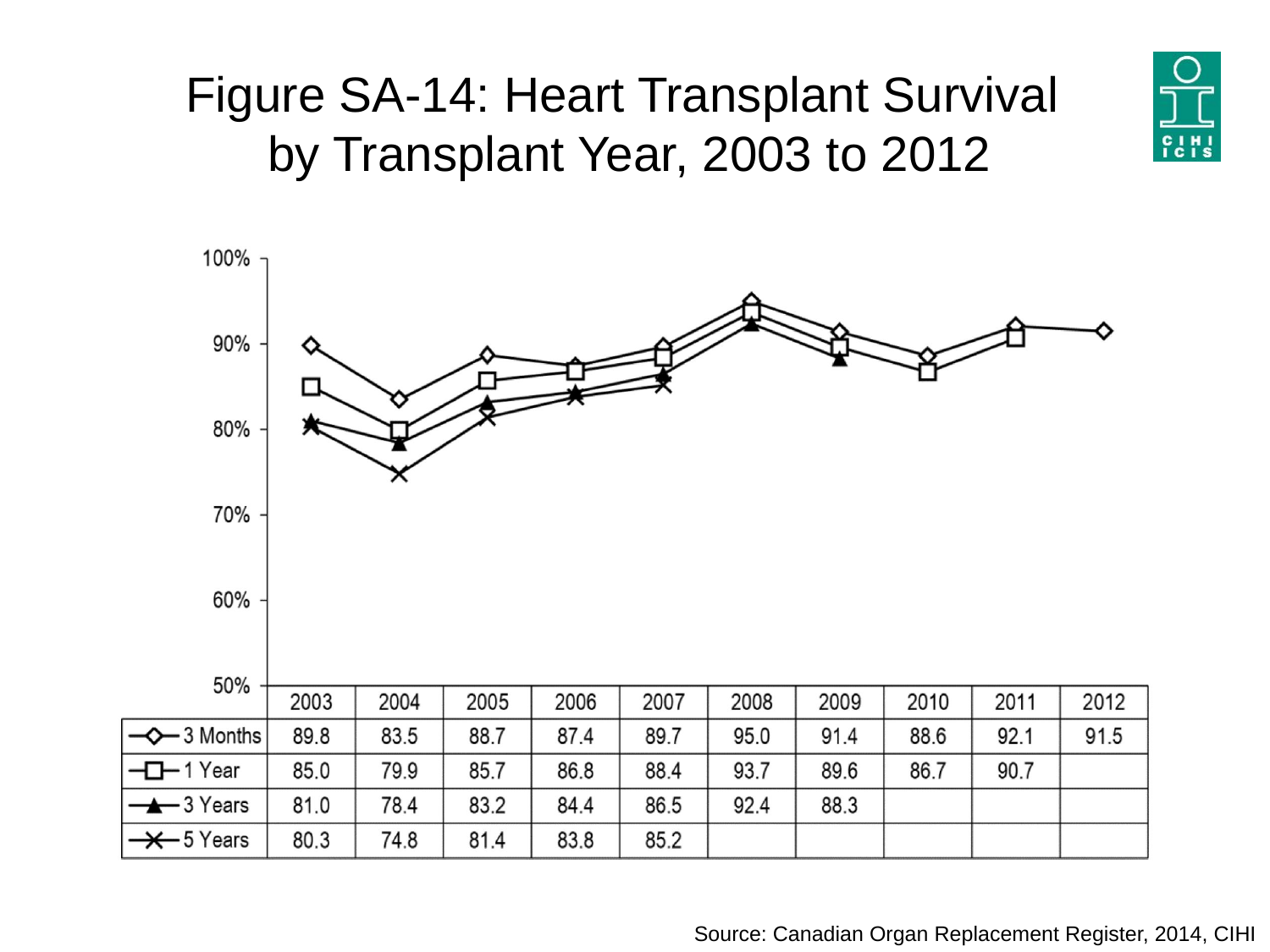

# Figure SA-14: Heart Transplant Survival by Transplant Year, 2003 to 2012
Source: Canadian Organ Replacement Register, 2014, CIHI

## Slide 22
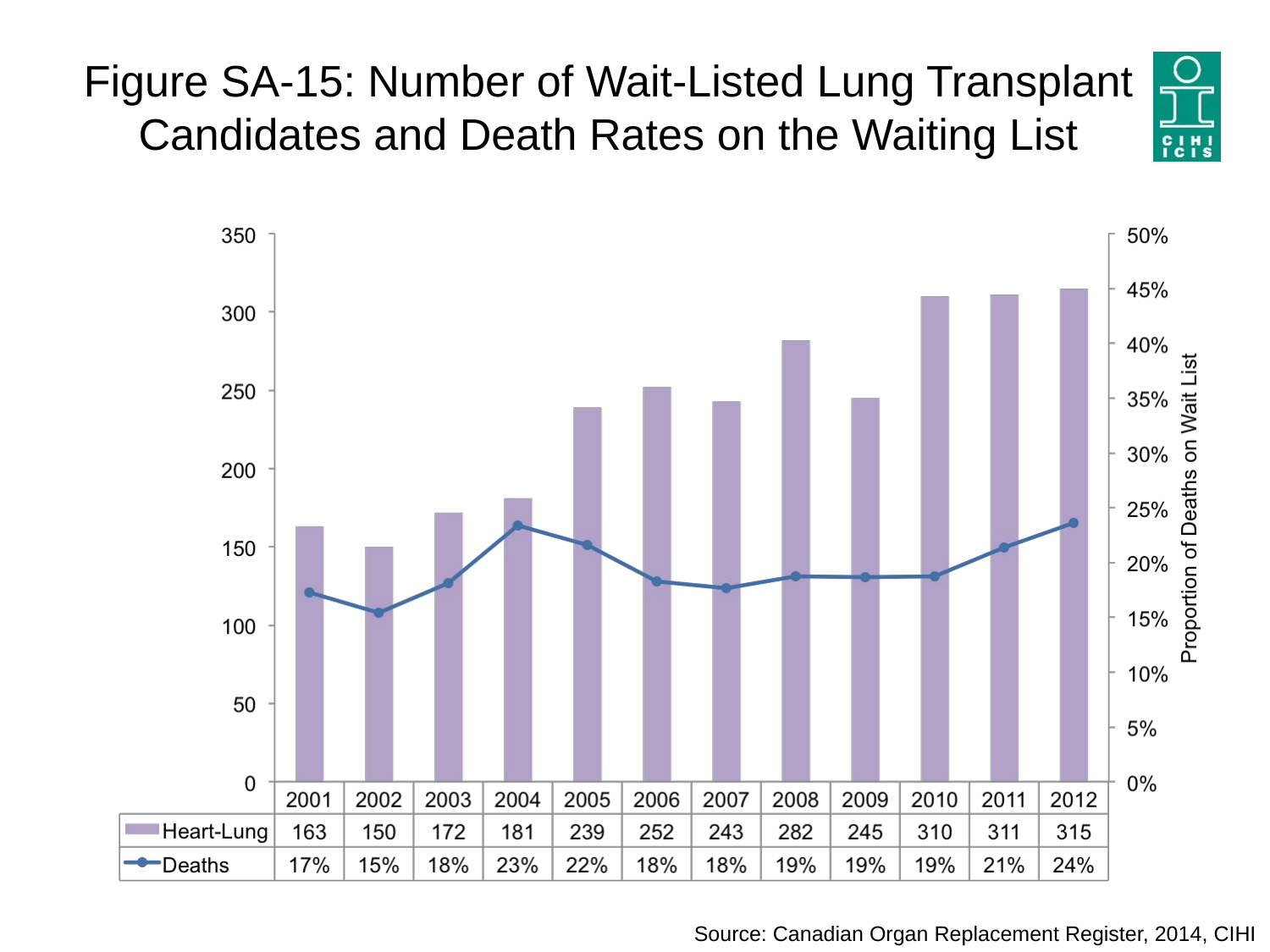

# Figure SA-15: Number of Wait-Listed Lung Transplant Candidates and Death Rates on the Waiting List
Source: Canadian Organ Replacement Register, 2014, CIHI

## Slide 23
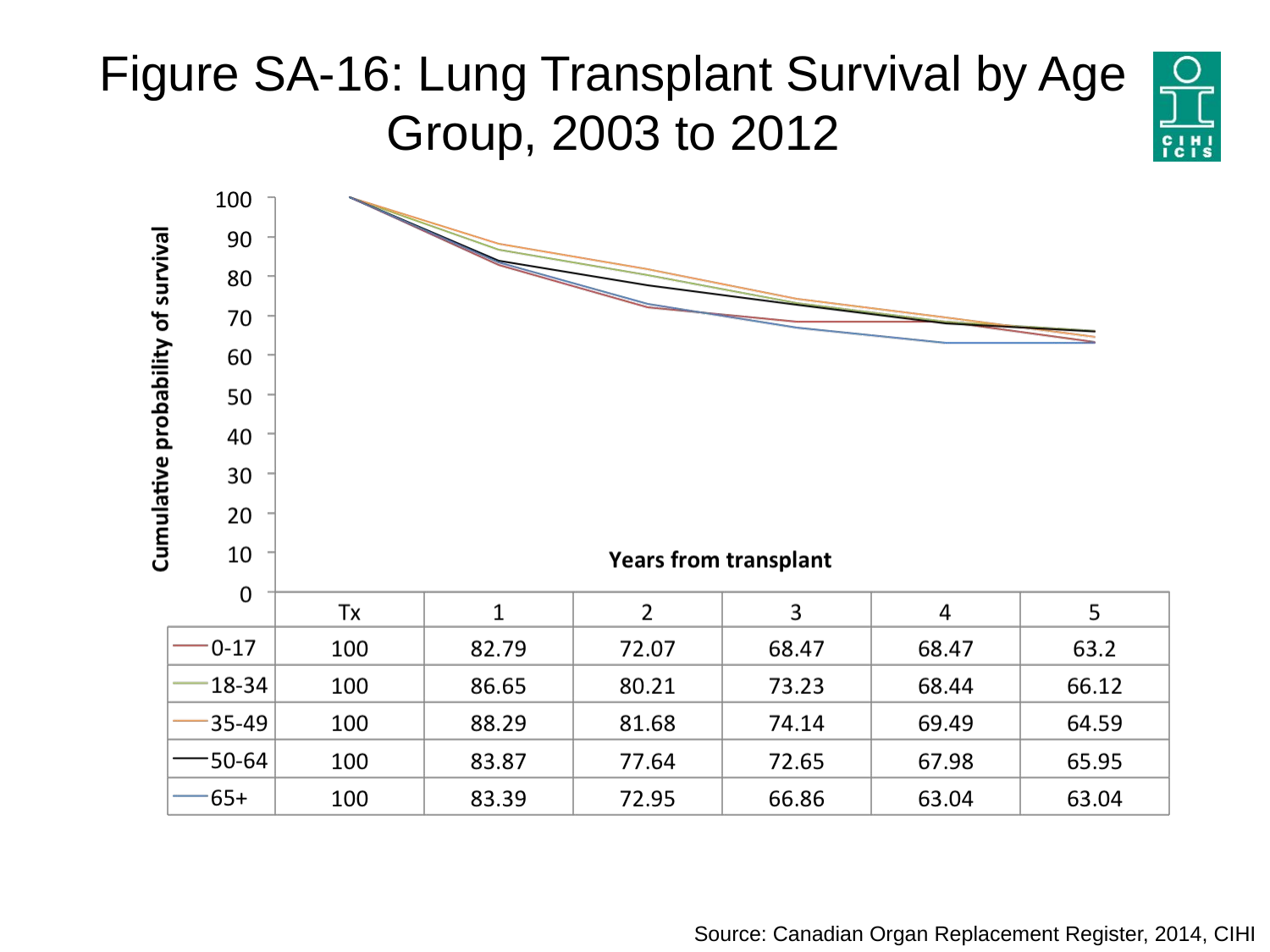

# Figure SA-16: Lung Transplant Survival by Age Group, 2003 to 2012
Source: Canadian Organ Replacement Register, 2014, CIHI

## Slide 24
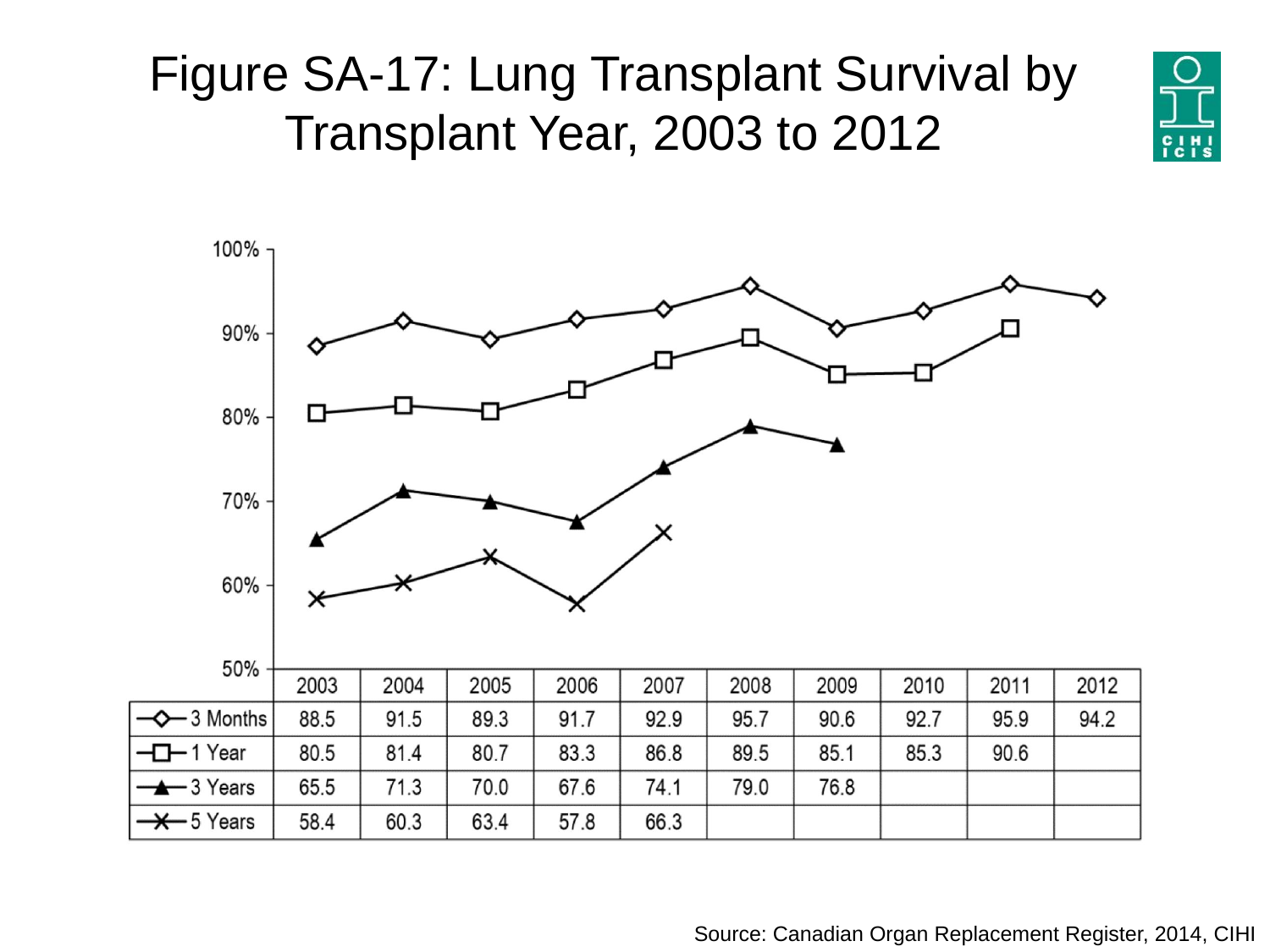

# Figure SA-17: Lung Transplant Survival by Transplant Year, 2003 to 2012
Source: Canadian Organ Replacement Register, 2014, CIHI

## Slide 25
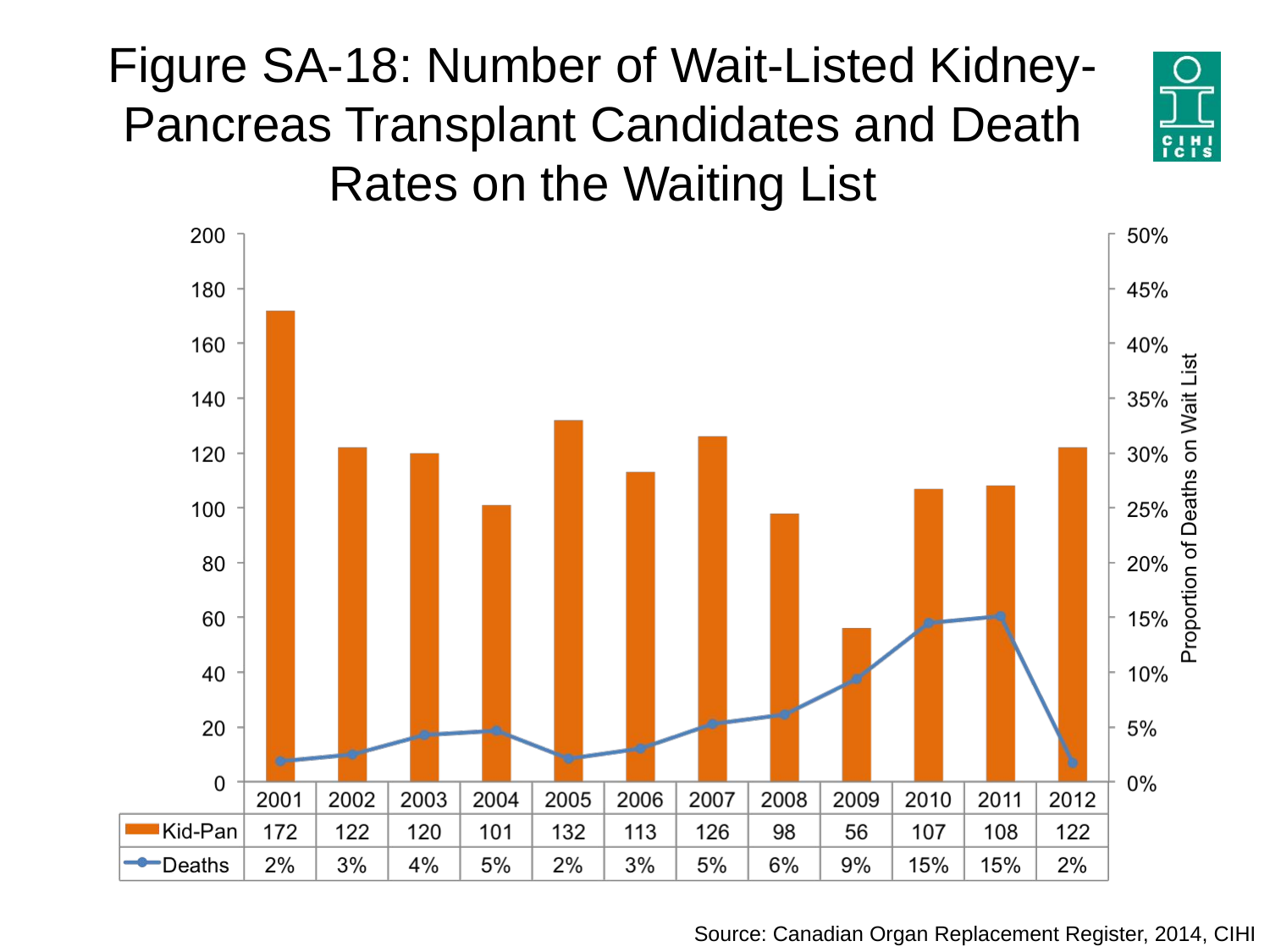

# Figure SA-18: Number of Wait-Listed Kidney-Pancreas Transplant Candidates and Death Rates on the Waiting List
Source: Canadian Organ Replacement Register, 2014, CIHI

## Slide 26
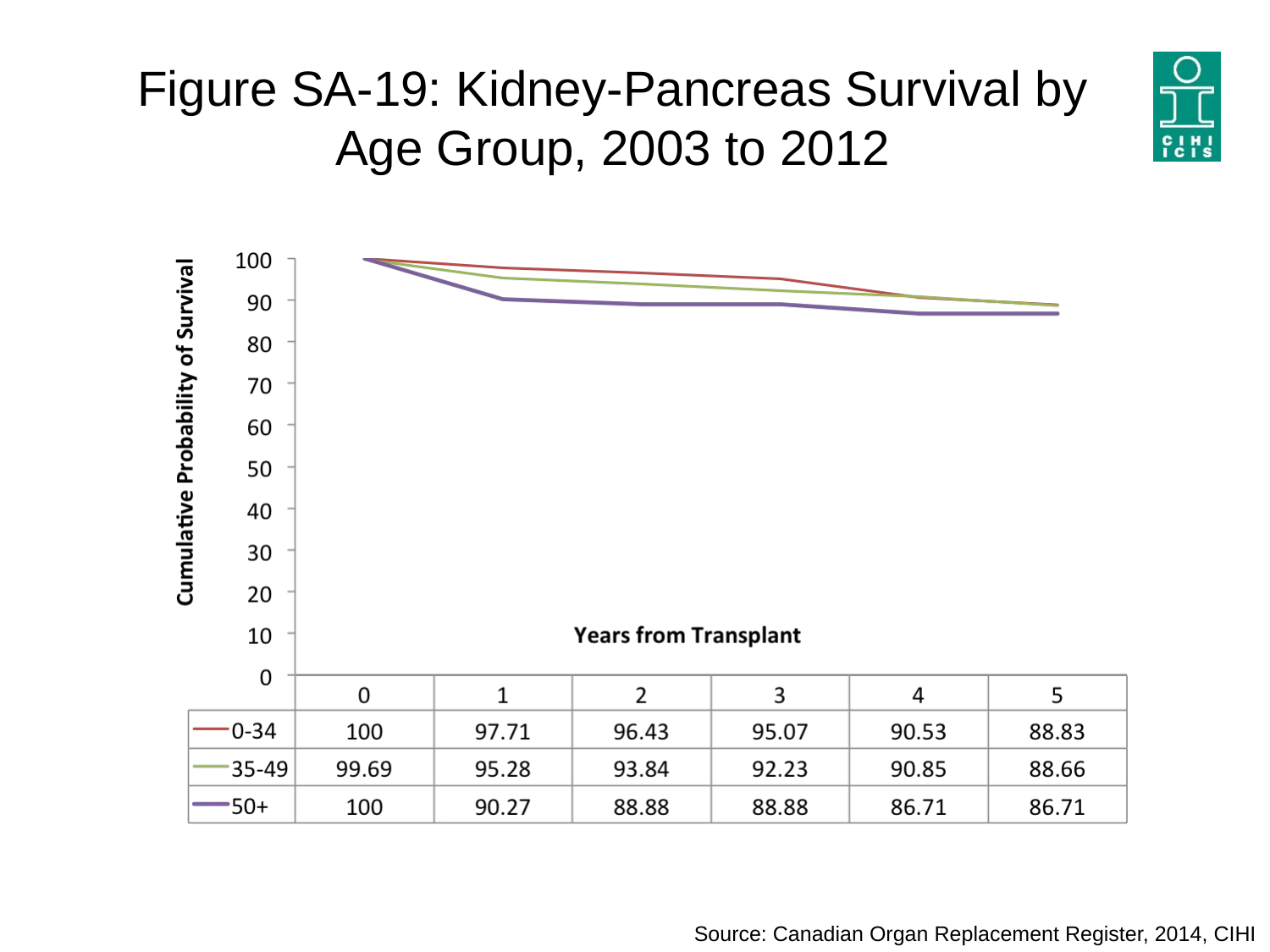

# Figure SA-19: Kidney-Pancreas Survival by Age Group, 2003 to 2012
Source: Canadian Organ Replacement Register, 2014, CIHI

## Slide 27
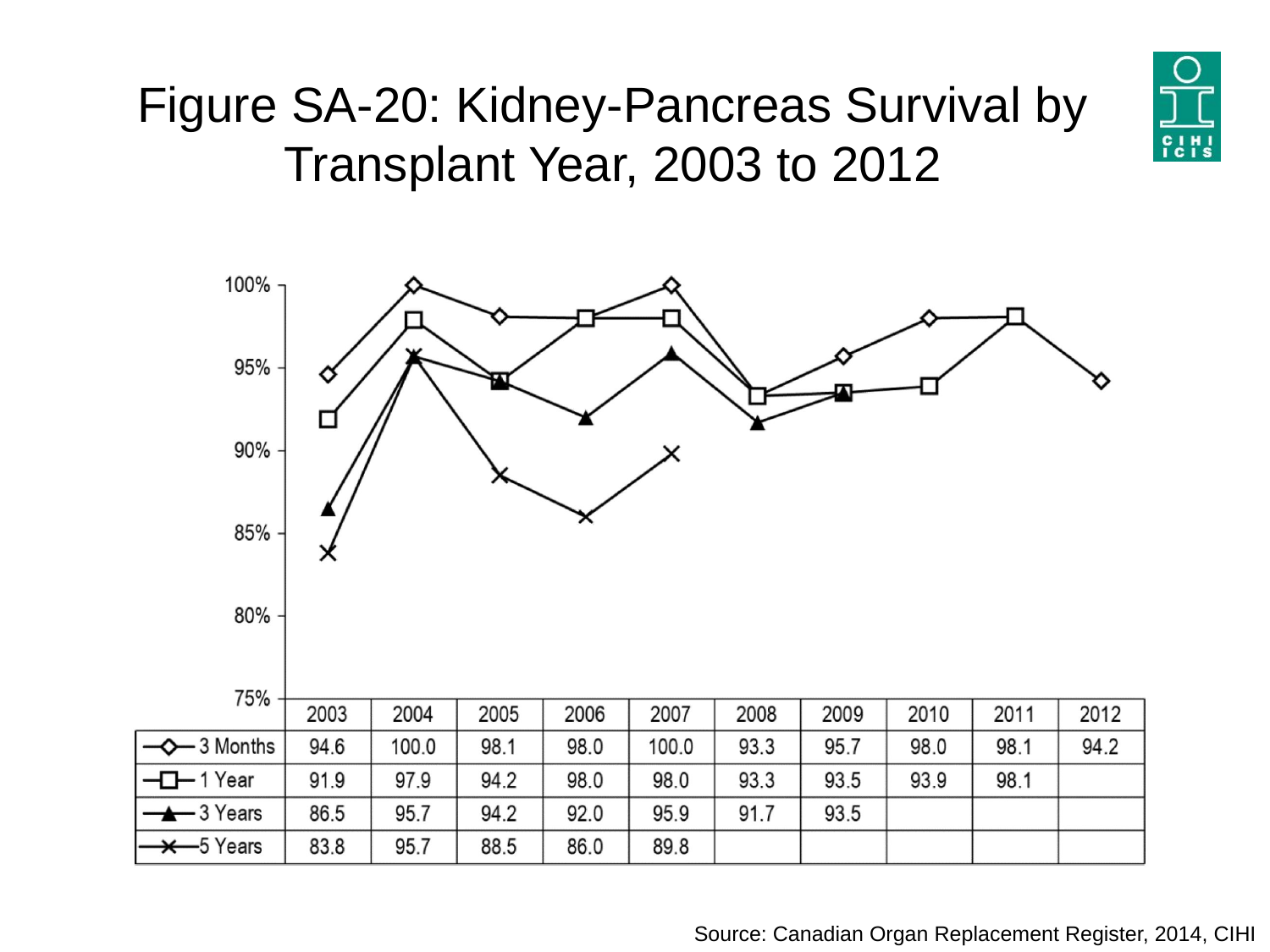

# Figure SA-20: Kidney-Pancreas Survival by Transplant Year, 2003 to 2012
Source: Canadian Organ Replacement Register, 2014, CIHI
