# Supplementary material for: Organ donation and transplantation in Canada: insights from the Canadian Organ Replacement Register
Source: Can J Kidney Health Dis. 2014 Dec 9;1:31. doi: 10.1186/s40697-014-0031-8 (PMC4349751; doi:10.1186/s40697-014-0031-8)
Supplement: Additional file 2: — Data Request Process. [file 40697_2014_31_MOESM2_ESM.pdf]

DATA REQUEST PROCESS

Subject to its privacy policy, CIHI makes its data available to enable responsive and effective health-system planning and decision-making. Researchers can request data from CORR via CIHI's custom data request process. Data can be retrieved at an aggregate or record level. A formal Data Request form, along with a Non-Disclosure/Confidentiality Agreement (NDCA) are completed by the data requestor and submitted to CORR. The CORR team then corresponds with the data requestor and prepares detailed specifications for the request. The CORR team processes the request according to the final specifications, and makes arrangement with the requestor to ensure secure delivery of the data.

For more information on CIHI's custom data request process, please see CIHI's Access Data page:

http://www.cihi.ca/CIHI-ext-portal/internet/EN/TabbedContent/standards+and+data+submission/data+requests/cihi012210

Canadian Organ Replacement Register
Deceased Donor Profile

SEND THIS CONFIDENTIAL INFORMATION TO:
Canadian Organ Replacement Register (CORR)
Canadian Institute for Health Information
4110 Yonge Street, Suite 300
Toronto, ON M2P 2B7
Tel.: 416-481-2002

Instructions
To be completed for all referrals, potential, and deceased donors.

Definitions
REFERRAL—Consultation/communication to a donor program about a deceased or dying patient who may be a potential organ donor. This patient will be assigned a unique identification number.

POTENTIAL DONOR—A referral who fulfills the general acceptance criteria for organ donation, for whom neurological death has been determined and consent for organ procurement has been obtained. Organ recovery may occur, but no recovered organs are transplanted.

Actual DECEASED DONOR—A donor from whom at least one organ or tissue has been transplanted.

SECTION A—REFERRAL/DONOR INFORMATION

Please provide all available information for referred organ donors.
program organizing organ recovery **(please check one):**

01 ☐ Halifax, N.S.	09 ☐ Saint John, N.B.	10 ☐ St. John's, N.L.
07 ☐ Montréal, Que.	13 ☐ Québec, Que.	02 ☐ Hamilton, Ont.
05 ☐ London, Ont.	11 ☐ Ottawa, Ont.	15 ☐ Kingston, Ont.
16 ☐ Toronto, Ont.	06 ☐ Winnipeg, Man.	14 ☐ Saskatoon, Sask.
17 ☐ Regina, Sask.	03 ☐ Calgary, Alta.	04 ☐ Edmonton, Alta.
12 ☐ Vancouver, B.C.	99 ☐ Other ________	

Recovery program donor number ____________________

Surname stem ____________ (Please enter the first 3 letters of the donor surname.)

Province, territory or state of residence ____________________

Country of residence ____________________

Referral accepted Yes ☐ No ☐ (If no, complete only sections A and B.)

If patient did not become a donor or no organs were recovered; indicate reason (see codes, upper right, this page) ____________________

Family consent obtained Yes ☐ No ☐

Neurological death determined Yes ☐ No ☐

Heart not beating/DCD Yes ☐ No ☐

Age Years (002–130) _______ Months (001–023) _______
Days (001–030) _______ Newborn (000) _______

Province, territory or state of death ____________________

Country of death ____________________

Sex ☐ Male ☐ Female ☐ Other (transsexual, hermaphrodite)

Blood Type ☐ A ☐ B ☐ AB ☐ O ☐ U

Race

01 ☐ Caucasian	02 ☐ Asian	03 ☐ Black
05 ☐ Indian subcontinent	08 ☐ Pacific islander	09 ☐ Aboriginal
10 ☐ Mid East/Arabian	11 ☐ Latin American	98 ☐ Unknown
99 ☐ Other/multiracial ______		

CODES—Reasons Patient Did Not Become Donor/Organs Not Recovered

03 Team/hospital logistics (team, hospital, transplantation resource issues)	10 Consent requested and denied
04 Medical reasons (instability, infection, etc.)	98 Unknown/not available
07 Consent not requested	99 Other reason: specify ______________
08 Neurological death not determined	
09 Refusal by medical examiner	

Donor Height and Weight (For deceased donors only; see definition above.)

Donor height ☐☐☐•☐☐☐ (cm)

Donor weight ☐☐☐•☐☐☐ (kg)

Conversion factors: 1 in. = 2.54 cm; 1 lb. = 0.45 kg)

Cause of Donor Death (For deceased donors only, see definition above.)

Please enter more specific information where applicable (e.g. type of drug overdose or cause of trauma).

01 ☐ Anoxia/hypoxia
02 ☐ CVA (stroke)
03 ☐ Trauma (not MVC)—describe ____________________
04 ☐ Motor vehicle collision
05 ☐ Overdose—describe ____________________
06 ☐ Primary CNS tumour
07 ☐ Ruptured cerebral aneurysm
08 ☐ Spontaneous intracranial hemorrhage
09 ☐ Gunshot
10 ☐ Intracranial event—describe ____________________
11 ☐ CNS infection
12 ☐ Carbon monoxide poisoning
13 ☐ Cerebral edema
14 ☐ Asthma, unspecified
15 ☐ SIDS (sudden infant death syndrome)
99 ☐ Other—describe ____________________

SECTION B—HOSPITAL INFORMATION (If referral was not accepted, consent was declined or no organs were recovered, please complete identifying hospital and date of admission only.)

Identifying hospital ____________ Date of admission (DD/MON/YYYY) |___|___|/|___|___|___|/|___|___|___|___|

Date death is determined (DD/MON/YYYY) |___|___|/|___|___|___|/|___|___|___|___|

Time of death is determined (HH/MM) |___|___|/|___|___|

Recovered hospital ____

Date of cross clamp (DD/MON/YYYY) |___|___|/|___|___|___|/|___|___|___|___| (Cross-clamp date is the same as the date of organ recovery.)

Cross-clamp time (HH/MM) |___|___|/|___|___|

Donor number ____

SECTION C—DONOR SEROLOGY AND RISK FACTORS (for actual donors only)

Donor Serology Status
(Check those that apply by answering: P = positive, N = negative, U = unknown.)

Hepatitis BsAg	P ☐ N ☐ U ☐	Epstein-Barr virus	P ☐ N ☐ U ☐
Hepatitis BcAb	P ☐ N ☐ U ☐	HIV	P ☐ N ☐ U ☐
Hepatitis C	P ☐ N ☐ U ☐	CMV	P ☐ N ☐ U ☐
HTLV type I and II (human T-cell lymphotropic virus)			P ☐ N ☐ U ☐

Donor HLA A____ ____ B____ ____ C____ ____ DR____ ____ DQ____ ____

Donor Risk Factors
(Check those that apply by answering: Y = yes, N = no, U = unknown.)

Smoker	Y ☐ N ☐ U ☐	Diabetes	Y ☐ N ☐ U ☐
Hypertension	Y ☐ N ☐ U ☐	Hyperlipidemia	Y ☐ N ☐ U ☐
Coronary artery disease	Y ☐ N ☐ U ☐	Creatinine at death>1.5 mg/dl	Y ☐ N ☐ U ☐

SECTION D—ADDITIONAL ORGAN INFORMATION (Please complete for all donors)

Inotropes at Time of Recovery (Check all that apply and the dosage.)

☐ Digoxin ➔	☐ High dose	☐ Dobutamine ➔	☐ High dose	☐ Dopamine ➔	☐ High dose
☐ Amrinone ➔	☐ High dose	☐ Milrinone ➔	☐ High dose	☐ Epinephrine ➔	☐ High dose
☐ Norepinephrine ➔	☐ High dose	☐ Isoproterenol ➔	☐ High dose	☐ Phenylephrine ➔	☐ High dose
☐ Vasopressin ➔	☐ High dose	☐ Other (specify): ______________________________ ➔ ☐ High dose			

Echo assessment:	☐ Not done	☐ Done (If done, please check):	☐ Normal function	☐ Abnormal function	☐ Unknown
ECG:	☐ Not done	☐ Done (If done, please check):	☐ Normal	☐ Abnormal	☐ Unknown
Coronary angiogram:	☐ Not done	☐ Done (If done, please check):	☐ Normal	☐ Abnormal	☐ Unknown

SECTION E—ORGAN-SPECIFIC INFORMATION

(Please answer for all organs.)

CODES—Reasons Donors or Organs Not Recovered/Transplanted

01 No consent for a particular organ
02 No recipient (no suitability matched recipient)
03 Team/hospital logistics (team, hospital, transplantation resources issues)
04 Medical reasons (instability, infection, etc.)
05 Retrieval injury
06 No program
07 Used for research
08 Used for heart valves
09 Stored/preserved
10 Recipient not looked for
11 Organ exported to U.S.
98 Unknown/not available
99 Other reason: specify __________

Donor Organ	Recovered		Transplanted		Reason Not Recovered/ Transplanted (See codes above.)	Organ Sent to (Indicate hospital or program.)	Recipient Name (Indicate if known.)
Double kidney/enbloc	☐ Yes	☐ No	☐ Yes	☐ No	____________	____________	____________
Right Kidney	☐ Yes	☐ No	☐ Yes	☐ No	____________	____________	____________
Left Kidney	☐ Yes	☐ No	☐ Yes	☐ No	____________	____________	____________
Heart	☐ Yes	☐ No	☐ Yes	☐ No	____________	____________	____________
Liver (whole organ)	☐ Yes	☐ No	☐ Yes	☐ No	____________	____________	____________
Liver, right lobe	☐ Yes	☐ No	☐ Yes	☐ No	____________	____________	____________
Liver, left lobe	☐ Yes	☐ No	☐ Yes	☐ No	____________	____________	____________
Liver, lateral segment	☐ Yes	☐ No	☐ Yes	☐ No	____________	____________	____________
Pancreas—whole	☐ Yes	☐ No	☐ Yes	☐ No	____________	____________	____________
Pancreas—segment	☐ Yes	☐ No	☐ Yes	☐ No	____________	____________	____________
Pancreas—islet cells	☐ Yes	☐ No	☐ Yes	☐ No	____________	____________	____________
Heart lung	☐ Yes	☐ No	☐ Yes	☐ No	____________	____________	____________
Bilateral lungs/enbloc	☐ Yes	☐ No	☐ Yes	☐ No	____________	____________	____________
Right lung	☐ Yes	☐ No	☐ Yes	☐ No	____________	____________	____________
Left lung	☐ Yes	☐ No	☐ Yes	☐ No	____________	____________	____________
Intestine	☐ Yes	☐ No	☐ Yes	☐ No	____________	____________	____________
Cluster (liver, sm. intestine, pancreas, stomach)	☐ Yes	☐ No	☐ Yes	☐ No	____________	____________	____________
Other multivisceral/bowel combination (specify organs): ________________	☐ Yes	☐ No	☐ Yes	☐ No	____________	____________	____________

Formulaire disponible en français.

Canadian Organ Replacement Register
Living Donor Profile

SEND THIS CONFIDENTIAL INFORMATION TO:
Canadian Organ Replacement Register (CORR)
Canadian Institute for Health Information
4110 Yonge Street, Suite 300
Toronto, ON M2P 2B7
Tel.: 416-481-2002

Instructions:
To be completed by the transplant program. Please attach this form to the relevant transplant recipient form.

SECTION A—DONOR INFORMATION

Donor Type

13 ☐ Autograft (islet)

Living, biologically related

03 ☐ Sibling   05 ☐ Other relative (e.g. mother's sister)

02 ☐ Parent   04 ☐ Offspring

Living, biologically unrelated

07 ☐ Spouse   06 ☐ Other living unrelated (emotional relationship, e.g. in-law) Specify: ____________________

12 ☐ Domino   10 ☐ Anonymous/altruistic   15 ☐ Paired

13 ☐ Autograft   14 ☐ Fetal Tissue (islet cells)

Transplant hospital ____

Hospital's donor code ____

Donor last name stem (first 3 letters) ______________________________

Province, territory or state of residence ____________________________

If not from Canada, country of residence ____________________________

Age ________ years

Sex ☐ Male ☐ Female ☐ Other

Blood Type ☐ A ☐ B ☐ AB ☐ O ☐ U

Race

01 ☐ Caucasian   02 ☐ Asian   03 ☐ Black   05 ☐ Indian subcontinent

08 ☐ Pacific islander   09 ☐ Aboriginal   10 ☐ Mid East /Arabian

11 ☐ Latin American   98 ☐ Unknown   99 ☐ Other/multiracial __________

Height ☐☐☐•☐☐☐ (cm)

(Conversion factor: 1 in. = 2.54 cm)

Weight ☐☐☐•☐☐☐ (kg)

(Conversion factor: 1 lb. = 0.45 kg)

SECTION B—HOSPITAL INFORMATION

Date of admission |___|___|/|___|___|___|/|___|___|___|___| (DD/MON/YYYY)

Date of cross clamp |___|___|/|___|___|___|/|___|___|___|___| (DD/MON/YYYY)

Time of cross clamp |___|___|/|___|___| (HH/MM)

SECTION C—DONOR SEROLOGY AND RISK FACTORS

Donor Serology Status

(Check those that apply: P = positive, N = negative, U = unknown.)

Hepatitis BsAg	P ☐	N ☐	U ☐	Epstein-Barr virus	P ☐	N ☐	U ☐
Hepatitis BcAb	P ☐	N ☐	U ☐	HIV	P ☐	N ☐	U ☐
Hepatitis C	P ☐	N ☐	U ☐	CMV	P ☐	N ☐	U ☐

HTLV type I and II (human T-cell lymphotropic virus) P ☐ N ☐ U ☐

*Donor HLA A____ ____ B____ ____ C____ ____ DR____ ____ DQ____ ____

*Note: CORR enters the lowest haplotype first.

Donor Risk Factors

(Check those that apply: Y = yes, N = no, U = unknown.)

Smoker	Y ☐	N ☐	U ☐	Hyperlipidemia	Y ☐	N ☐	U ☐
Diabetes	Y ☐	N ☐	U ☐	Coronary artery disease	Y ☐	N ☐	U ☐
Hypertension	Y ☐	N ☐	U ☐				

SECTION D—ORGAN SPECIFIC INFORMATION

Organ recovered:

11 ☐ Left kidney   23 ☐ Liver lateral segment

12 ☐ Right kidney   41 ☐ Lung left lobe

21 ☐ Liver left lobe   42 ☐ Lung right lobe

22 ☐ Liver right lobe

Recipient last name ____

Recipient date of birth |___|___|/|___|___|___|/|___|___|___|___| (DD/MON/YYYY)

Canadian Institute for Health Information
Institut canadien d'information sur la santé

Formulaire disponible en français.

Form LDP-2014

Canadian Organ Replacement Register
Kidney Transplant Recipient Registration Form

SEND THIS CONFIDENTIAL INFORMATION TO:
Canadian Organ Replacement Register (CORR)
Canadian Institute for Health Information
4110 Yonge Street, Suite 300
Toronto, ON M2P 2B7
Tel.: 416-481-2002

SECTION A—RECIPIENT INFORMATION

Transplant hospital ____________________ (name and city)

Patient ID ____________________

Last name ____________________ First/middle name ____________________

Former name ____________________

Sex ☐ Male ☐ Female ☐ Other

Blood type ☐ A ☐ B ☐ AB ☐ O ☐ U

Race

01 ☐ Caucasian 02 ☐ Asian 03 ☐ Black 05 ☐ Indian subcontinent

08 ☐ Pacific islander 09 ☐ Aboriginal 10 ☐ Mid East/Arabian

11 ☐ Latin American 98 ☐ Unknown 99 ☐ Other/multiracial ________

Date of birth |___|___|/|___|___|___|/|___|___|___|___| (DD/MON/YYYY)

Health card number ____________________

Prov. or terr. of health card ____________________

Address (city) ____________________

Province or territory ____________________ Postal code ____________________

SECTION B—TRANSPLANT INFORMATION

Waiting List Information

Date patient first placed on waiting list

(for this transplant) |___|___|/|___|___|___|/|___|___|___|___| (DD/MON/YYYY)

Date of Transplant |___|___|/|___|___|___|/|___|___|___|___| (DD/MON/YYYY)

Graft number ____________________

☐ Single kidney transplant ☐ Double kidney/enbloc

☐ Combination transplant

If combination, specify other organ(s) ____________________

Please complete section B of relevant transplant recipient registration form for other organ(s).

Recipient Serology Status at Time of Transplant

(Please check one of the acceptable values: P = positive, N = negative or U = unknown.)

Hepatitis BsAg	P ☐	N ☐	U ☐	Epstein-Barr virus	P ☐	N ☐	U ☐
Hepatitis BcAb	P ☐	N ☐	U ☐	HIV	P ☐	N ☐	U ☐
Hepatitis C	P ☐	N ☐	U ☐	CMV	P ☐	N ☐	U ☐

Donor specific antibodies Y ☐ N ☐

Class I PRA current_____ % Class I PRA peak _______%

Class II PRA current_____ % Class II PRA peak _______%

* Methods: CDC ☐ ELISA ☐ Flow ☐ Luminex ☐ Other _________

* The most sensitive method should be entered if more than one method is used by the laboratory.

*Recipient HLA A___ ___ B___ ___ C___ ___ DR___ ___ DQ___ ___

*Note: CORR enters the lowest haplotype first.

SECTION B—TRANSPLANT INFORMATION (continued)

Primary Renal Disease (diagnosis reported at first treatment)

Code ____________ (See codes on back of form.) ☐ Retransplant

Describe ____________________

Diagnosis at time of first transplant

Code ____________ (See codes on back of form.)

Describe ____________________

Donor organ kidney ☐ Right kidney ☐ Left kidney ☐ Both kidneys

Laparoscopic nephrectomy used? Y ☐ N ☐ U ☐

(At time of transplant) Recipient height ☐☐☐•☐☐☐ (cm)

(Conversion factor: 1 in. = 2.54 cm)

Recipient weight ☐☐☐•☐☐☐ (kg)

(Conversion factor: 1 lb. = 0.45 kg)

Was patient on dialysis for ESRD pre-transplant? ☐ Yes ☐ No ☐ Unknown

Delayed graft function? ☐ Yes ☐ No ☐ Unknown

Did patient receive dialysis treatment within the first week of transplantation? ☐ Yes ☐ No ☐ Unknown

Risk Factors Existing at Time of Transplant
(Please check one of the acceptable values: Y = yes, N = no or U = unknown.)

Angina	Y ☐	N ☐	U ☐	Peripheral vascular disease	Y ☐	N ☐	U ☐
Malignancy	Y ☐	N ☐	U ☐	Previous myocardial infarction	Y ☐	N ☐	U ☐
Pulmonary edema	Y ☐	N ☐	U ☐	Chronic obstr. lung disease	Y ☐	N ☐	U ☐
Diabetes type 1	Y ☐	N ☐	U ☐	Diabetes type 2	Y ☐	N ☐	U ☐
Hypertension	Y ☐	N ☐	U ☐	Prev. cerebrovascular accident	Y ☐	N ☐	U ☐

Cold ischemic time ____________ (min)

SECTION C—DONOR INFORMATION

☐ Living 12 ☐ Domino donor ➔ For a living or domino donor, please complete a living donor profile and attach to this form.

01 ☐ Deceased donor 98 ☐ Unknown out of country transplant

To facilitate matching, please complete the following:

Program organizing organ recovery ____________________

Originating OPO donor number ____________________

Surname stem (first 3 letters of donor surname) ___________

Age Years (002–130) ______ Months (001–023) ______

Days (001–030) ______ Newborn (000) _______

Sex ☐ Male ☐ Female ☐ Other

*Donor HLA A____ ____ B____ ____ C____ ____ DR____ ____ DQ____ ____

*Note: CORR enters the lowest haplotype first.

Date of cross clamp (DD/MON/YYYY) |___|___|/|___|___|___|/|___|___|___|___|

(Cross-clamp date is the same as the date of organ recovery.)

Cross-clamp time (HH/MM) |___|___|/|___|___|

Canadian Institute for Health Information
Institut canadien d'information sur la santé

PRIMARY RENAL DIAGNOSIS

CODE DESCRIPTION

00 Chronic renal failure, etiology uncertain

GLOMERULONEPHRITIS/AUTOIMMUNE DISEASES

05 Mesangial proliferative glomerulonephritis
06 Minimal lesion glomerulonephritis
07 Post-strep glomerulonephritis
08 Rapidly progressive glomerulonephritis
09 Focal glomerulonephritis—adults
10 Glomerulonephritis, histologically not examined
11 Severe nephrotic syndrome with focal sclerosis (pediatric patients only)
12 IgA nephropathy (proven by immunofluorescence) (not code 85)
13 Dense deposit disease (proven immunofluorescence and/ or electron microscopy) (MPGN type II)
14 Membranous nephropathy
15 Membranoproliferative glomerulonephritis (MGPN type I)
16 Idiopathic crescentic glomerulonephritis (diffuse proliferative)
17 Congenital nephrosis or congenital nephrotic syndrome (pediatric only)
19 Glomerulonephritis, histologically examined—specify
73 Polyarteritis
74 Wegener's granulomatosis
84 Lupus erythematosus
85 Henoch-Schonlein purpura
86 Goodpasture's syndrome
87 Scleroderma
88 Hemolytic uremic syndrome -)

NEPHROPATHY—DRUG INDUCED

30 Nephropathy caused by drugs or nephrotoxic agents— cause not specified
31 Nephropathy due to analgesic drugs
32 Nephropathy due to cisplatin
33 Nephropathy due to cyclosporin A
39 Nephropathy caused by other specific drug—specify

POLYCYSTIC KIDNEYS

41 Polycystic kidneys, adult type (dominant)
42 Polycystic kidneys, infantile and juvenile types (recessive)

DIABETES

80 Diabetic nephropathy associated with type 1
81 Diabetic nephropathy associated with type 2

CONGENITAL/HEREDITARY RENAL DISEASES

21 Pyelonephritis/interstitial nephritis associated with neurogenic bladder
22 Pyelonephritis/interstitial nephritis due to congenital obstructive uropathy with or without vesico-ureteric reflux
24 Pyelonephritis/interstitial nephritis due to vesico-ureteric reflux without obstruction
40 Cystic kidney disease, type unspecified
41 Polycystic kidneys, adult type (dominant)
42 Polycystic kidneys, infantile and juvenile type (recessive)
43 Medullary cystic disease, including nephronophthisis
49 Cystic kidney disease, other specified type—specify
50 Hereditary/familial nephropathy—type unspecified
51 Hereditary nephritis with nerve deafness (Alport's syndrome)
52 Cystinosis
53 Oxalosis
54 Fabry's disease
55 Drash syndrome
58 Posterior urethral valves
59 Hereditary nephropathy, other—specify
60 Congenital renal hypoplasia—specify
61 Oligomeganephronic hypoplasia
62 Segmental renal hypoplasia (Ask-Upmark kidney)
63 Congenital renal dysplasia with or without urinary tract malformation
66 Syndrome of agenesis of abdominal muscles (prune belly syndrome)

RENAL VASCULAR DISEASE

70 Renal vascular disease—type unspecified
71 Malignant hypertension (no primary renal disease)
72 Renal vascular disease due to hypertension (no primary renal disease)
73 Polyarteritis nodosa
78 Atheroembolic renal disease
79 Renal vascular disease, classified (nephrosclerosis, renal vascular thrombosis)

OTHER

20 Pyelonephritis/interstitial nephritis, cause not specified
23 Pyelonephritis/interstitial nephritis due to acquired obstructive uropathy—specify
25 Pyelonephritis/interstitial nephritis due to urolithiasis
29 Pyelonephritis, other causes
56 Sickle cell nephropathy
57 Wilms' tumour
82 Multiple myeloma
83 Amyloid
89 Multi-system disease, other—specify
90 Cortical or acute tubular necrosis
91 Tuberculosis
92 Gout
93 Nephrocalcinosis and hypercalcemic nephropathy
94 Balkan nephropathy
95 Kidney tumour
96 Traumatic or surgical loss of kidney
97 HIV nephropathy
99 Other identified renal disorders—specify ____________________

Formulaire disponible en français.

Canadian Organ Replacement Register
Recipient Outcome for Kidney Transplant

SEND THIS CONFIDENTIAL INFORMATION TO:
Canadian Organ Replacement Register (CORR)
Canadian Institute for Health Information
4110 Yonge Street, Suite 300
Toronto, ON M2P 2B7
Tel.: 416-481-2002

Complete this form to reflect the situation at your facility for patient lost to follow death, graft failure, transfer or the patient being followed at another hospital.

SECTION A—RECIPIENT INFORMATION

Transplant hospital ______________________________
(name and city)

Patient ID ______________________________

Last name ______________________________

First/middle name ______________________________

Former name ______________________________

Date of birth |___|___|/|___|___|___|/|___|___|___|___| (DD/MON/YYYY)

Health card number ______________________________

Prov. or terr. of health card ______________________________

Address (city) ______________________________

Province or Territory ____________ Postal code ____________

Hospital followed at ______________________________

(Enter only if different than transplant hospital.)

SECTION B—RECIPIENT OUTCOME

Date of Transplant |___|___|/|___|___|___|/|___|___|___|___| (DD/MON/YYYY)

Graft Number ______________________________

Patient Status (Please check one.)

Patient alive ☐ Transfer ☐ Lost to follow-up ☐ Died ☐

Transfer Hospital Name (Please check one of the following:) ☐ To **OR** From ☐

Name of Transfer Hospital: ______________________________

Date of transfer: |___|___|/|___|___|___|/|___|___|___|___| (DD/MON/YYYY)

Date of lost to follow: |___|___|/|___|___|___|/|___|___|___|___| (DD/MON/YYYY)

If deceased (Please check one of the following and enter cause of death.)

☐ Died with a functioning graft

OR

☐ Died due to graft failure (Check cause of graft failure below.)

Enter cause of death ____________ (codes on back of page)

Date of death: |___|___|/|___|___|___|/|___|___|___|___| (DD/MON/YYYY)

SECTION B—RECIPIENT OUTCOME (continued)

If alive with failed graft or died due to graft failure, please complete this section.

Date of graft failure |___|___|/|___|___|___|/|___|___|___|___| (DD/MON/YYYY)

Check cause of graft failure below:

00 ☐ Uncertain/Unknown

01 ☐ Hyperacute rejection

11 ☐ Primary non-function

18 ☐ De novo malignancy (graft)

23 ☐ Vascular thrombosis (graft)

26 ☐ Vascular operative problems

27 ☐ Ureteric operative porblems

28 ☐ Surgical complications

30 ☐ Rejection after stopping drugs

36 ☐ Cyclosporin toxicity

63 ☐ Acute rejection

64 ☐ Chronic rejection

67 ☐ Recurrent disease

68 ☐ Infection and rejection

69 ☐ Infection of the graft

99 ☐ Other cause of graft failure (describe) ______________

CAUSE OF DEATH/COMORBID COMPLICATION (RECIPIENT)

GENERIC
00 Cause of death uncertain/not determined

CARDIAC
11 Myocardial ischemia and infarction
12 Hyperkalemia
13 Hemorrhagic pericarditis
14 Other causes of cardiac failure
15 Cardiac arrest, cause unknown
16 Hypertensive cardiac failure
17 Hypokalemia
18 Fluid overload

VASCULAR
21 Pulmonary embolus
22 Cerebrovascular accident
24 Hemorrhage from graft site—specify
25 Hemorrhage from vascular access or dialysis circuit
26 Hemorrhage from ruptured vascular aneurysm (not code 22 or 23)
27 Hemorrhage from surgery (not codes 23 to 26)—specify
28 Other hemorrhage (not codes 23 to 27)
55 Vascular thrombosis
56 Pulmonary vein stenosis
57 Stent/balloon complication

INFECTION
03 Infection (bacterial)—specify site
04 Infection (viral)—specify site
05 Infection (fungal)—specify site
06 Cytomegalovirus
07 Epstein-Barr virus
08 Pneumocystic carinii pneumonia (PCP)
09 Protozoal/parasitic infection (includes toxoplasmosis)
10 Wound infection—specify site
34 Infections elsewhere (except viral hepatitis codes 41 and 42)
35 Septicemia/sepsis—specify source
36 Tuberculosis (lung)
37 Tuberculosis (elsewhere)
38 Generalized viral infection—specify viral agent
39 Peritonitis (not code 70)

LIVER DISEASE
41 Liver, due to hepatitis B virus
42 Liver, other viral hepatitis
43 Liver, drug toxicity—specify drug
44 Cirrhosis, not viral
45 Cystic liver disease
46 Liver failure, cause unknown
74 Liver, due to hepatitis C virus

GASTROINTESTINAL
02 Gastrointestinal tumour with or without perforation
20 Acute gastroenteritis with dehydration
23 Gastrointestinal hemorrhage
29 Mesenteric infarction
62 Pancreatitis
68 Perforation of peptic ulcer
70 Sclerosing (or adhesive) peritoneal disease
72 Perforation of colon/small bowel

SOCIAL
50 Drug abuse (excludes alcohol abuse)
51 Patient refused further treatment
52 Suicide
53 Therapy ceased for any other reason
54 Alcohol abuse

ACCIDENT
81 Accident related to treatment
82 Accident unrelated to treatment

MISCELLANEOUS
30 Hypertension
40 Diabetic keto acidosis (DKA)
64 Cachexia
66 Malignant disease possibly induced by immunosuppressive therapy—specify primary site
67 Malignant disease (not code 66)—specify primary site
69 Dementia
90 Multi-system failure
99 Other identified cause of death—specify

RESPIRATORY
19 Acute respiratory distress syndrome (ARDS)
31 Pulmonary infection (bacterial)
32 Pulmonary infection (viral)
33 Pulmonary infection (fungal)
49 Bronchiolitis obliterans

RENAL DISEASE
47 Acute renal failure
48 Chronic renal failure
61 Uremia caused by kidney transplant failure

METABOLIC
59 Drug-related toxicity—specify drug

HEMATOLOGIC
63 Bone marrow depression
71 Thrombocytopenia
73 Thrombosis—specify

NEUROLOGIC
75 Drug neurotoxicity—specify drug
76 Status epilepticus
77 Neurologic infection—specify infectious agent

Canadian Organ Replacement Register
Liver Transplant Recipient Registration Form

SEND THIS CONFIDENTIAL INFORMATION TO:
Canadian Organ Replacement Register (CORR)
Canadian Institute for Health Information
4110 Yonge Street, Suite 300
Toronto, ON M2P 2B7
Tel.: 416-481-2002

SECTION A—RECIPIENT INFORMATION

Transplant hospital ______________________________
(name and city)

Patient ID ______________________________

Last name ______________________________

First/middle name ______________________________

Former name ______________________________

Sex ☐ Male ☐ Female ☐ Other

Blood Type ☐ A ☐ B ☐ AB ☐ O ☐ U

Race
01 ☐ Caucasian 02 ☐ Asian 03 ☐ Black 05 ☐ Indian subcontinent
08 ☐ Pacific islander 09 ☐ Aboriginal 10 ☐ Mid East/Arabian
11 ☐ Latin American 98 ☐ Unknown 99 ☐ Other/multiracial ___________

Date of birth |___|___|/|___|___|___|/|___|___|___|___| (DD/MON/YYYY)

Health card number ______________________________

Prov. or terr. of health card ______________________________

Address (city) ______________________________

Province or territory ______________________ Postal code __________________

(At time of transplant)

Recipient height ☐☐☐•☐☐☐ (cm)

(Conversion factor: 1 in. = 2.54 cm)

Recipient =weight ☐☐☐•☐☐☐ (kg)

(Conversion factor: 1lb. = 0.45 kg)

SECTION B—TRANSPLANT INFORMATION

Date patient first placed on waiting list (for this transplant)

|___|___|/|___|___|___|/|___|___|___|___| (DD/MON/YYYY)

Medical status when first placed on waiting list (Please check one.)

19 ☐ Status 1 (at home) 16 ☐ Status 1T (tumour patient)
17 ☐ Status 2 (hospitalized) 05 ☐ Status 3 (hospitalized ICU)
11 ☐ Status 3F (fulminant) 18 ☐ Status 4 (ICU—incubated and ventilated)
12 ☐ Status 4F (fulminant)

SECTION B—TRANSPLANT INFORMATION (continued)

Date moved to the final list status (Indicate date if not same as initial listing status.)

|___|___|/|___|___|___|/|___|___|___|___| (DD/MON/YYYY)

Medical Status at Time of Transplant

19 ☐ Status 1 (at home) 16 ☐ Status 1T (tumour patient)
17 ☐ Status 2 (hospitalized) 05 ☐ Status 3 (hospitalized ICU)
11 ☐ Status 3F (fulminant) 18 ☐ Status 4 (ICU—incubated and ventilated)
12 ☐ Status 4F (fulminant)

Date of transplant |___|___|/|___|___|___|/|___|___|___|___| (DD/MON/YYYY)

☐ Liver transplant **OR** ☐ Combination transplant

Graft number __________________

Specify other organ(s) ______________________________

Please complete Section B relevant transplant registration form for other organ(s).

Liver Diagnosis (See page 3.)

1 ______ 2 ______ 3 ______ 4 ______ ☐ Retransplant

Describe ______________________________

Recipient Serology Status at Time of Transplant

Please check one of the acceptable values: P = positive, N = negative or U = unknown.

Hepatitis B

Hepatitis BsAg P ☐ N ☐ U ☐ Hepatitis BcAb P ☐ N ☐ U ☐

Hepatitis B-DNA P ☐ _______(pg/ml) N ☐ U ☐

Treatment at time of transplant N ☐ Y ☐ ➔ *Check one:*
Interferon ☐
Lamivudine ☐
Other (specify) _________

Hepatitis C

Hepatitis C P ☐ N ☐ U ☐ *(If "N," skip to Epstein-Barr virus flag, below.)*

RNA detectable? ☐ No ☐ Yes ➔ *Specify level* ___________ ☐ Not collected

Genotype: ☐ 1 ☐ 2 ☐ 3 ☐ 4 ☐ 5 ☐ 6 ☐ Unknown

Treatment at time of transplant:

☐ Interferon ☐ Ribavirin ☐ Both Interferon and Ribavirin

Epstein-Barr virus P ☐ N ☐ U ☐

CMV P ☐ N ☐ U ☐

HIV P ☐ N ☐ U ☐

Donor specific antibodies Y ☐ N ☐

Class I PRA current_____ % Class I PRA peak _______%

Class II PRA current_____ % Class II PRA peak _______%

* Methods: CDC ☐ ELISA ☐ Flow ☐ Luminex ☐ Other ☐

* The most sensitive method should be entered if more than one method is used by the laboratory.

Standard crossmatch test P ☐ N ☐ U ☐

*Recipient HLA: A____ ____ B____ ____ C____ ____ DR____ ____ DQ____ ____

*Note: CORR enters the lowest haplotype first.

Recipient name ____

SECTION B—TRANSPLANT INFORMATION (continued)

Child-Pugh score at transplant |___|___| Creatinine at transplant |___|___|___|

Total serum bilirubin at transplant (µmol/L) |___|___|___| INR at transplant |___|.|___|___|

Split or Reduction Technique

Liver reduction (one recipient) Y ☐ N ☐ U ☐

Split liver (two recipients) Y ☐ N ☐ U ☐ Left ☐ Right ☐

Technique: ☐ In-situ ☐ Ex-situ ☐ Combination

Primary and Metastatic Tumours in the Liver? ☐ Yes (Complete shaded section.) ☐ No (Skip shaded section.)

Complete this section or attach copy of form submitted to the International Registry of Hepatic Tumors in Liver Transplantation (Baylor University Medical Centre).

Tumour markers (ng/ml); alpha-fetoprotein ____________________

Chorioembryonic antigen (CEA) ____________________

Number of nodules ____________Diameter of largest (cm) ____________

Bilobar ☐ Yes ☐ No Characteristics ☐ Multifocal ☐ Single

Histologic grade ____________ System used ____________

Vascular involvement ☐ Yes ☐ No

Spread at surgery ☐ None ☐ Periaortic ☐ Lungs, mediastinum ☐ Diaphragm ☐ Abdomen, other ☐ Hilar nodes

Adjunct Tumour Therapy

Therapy	Pre-op		Intra-op		Post-op		Specify agent (where applicable)
Embolization	Y	N					
Irradiation	Y	N	Y	N	Y	N	
Other treatment	Y	N	Y	N	Y	N	____________
Chemotherapy							
• Adriamycin	Y	N	Y	N	Y	N	
• 5-Fluorouracil	Y	N	Y	N	Y	N	
• 5-FU DR	Y	N	Y	N	Y	N	
• Cisplatin	Y	N	Y	N	Y	N	
• Other	Y	N	Y	N	Y	N	____________

Warm ischemic time (min) |___|___|___| Cold ischemic time (min) |___|___|___|

Rewarm time (min) |___|___|___|

SECTION C—DONOR INFORMATION

☐ Living 12 ☐ Domino donor ➔For a living or domino donor, please complete a living donor profile and attach to this form.

01 ☐ Deceased donor

To facilitate matching, please complete the following.

Program organizing organ recovery ____________________

Originating OPO donor number ____________________

Surname stem (first 3 letters of donor surname) ____________

Age Years (002–130) ________ Months (001–023) ________

Days (001–030) ________ Newborn (000) ________

Sex ☐ Male ☐ Female ☐ Other

*Donor HLA A____ ____ B____ ____ C____ ____ DR____ ____ DQ____ ____

*Note: CORR enters the lowest haplotype first.

Date of cross clamp (DD/MON/YYYY) |___|___|/|___|___|___|/|___|___|___|___|

(Cross-clamp date is the same as the date of organ recovery.)

Cross-clamp time (HH/MM) |___|___|/|___|___|

Codes—Primary Liver Diagnosis

ACUTE HEPATIC FAILURE (Fulminant)

01 Hepatitis—type A
02 Hepatitis—type B
61 Hepatitis—type C
58 Hepatitis—type non A,B,C
35 Hepatitis with delta
05 Toxic
04 Drug induced—other
56 Drug induced—acetaminophen
47 Other/fulminant hepatitic failure (Including Budd-Chiari and Wilson's disease)

CHRONIC HEPATIC FAILURE

12 Budd-Chiari
36 Byler's disease (intra-hepatic colestasis)
09 Cirrhosis—alcoholic
10 Cirrhosis—other
08 Cryptogenic cirrhosis
49 Post-necrotic cirrhosis
07 Primary biliary cirrhosis
14 Secondary biliary cirrhosis
45 Drug induced—other
42 Hepatitis—type A
43 Hepatitis—type B
60 Hepatitis—type C
59 Hepatitis—type non A, B, or C
51 Neonatal hepatitis
06 Autoimmune chronic active hepatitis
13 Primary bliary atresia
11 Sclerosing cholangitis
46 Toxic
15 Watson-Alagille disease (arterio-hepatic dysplasia)
62 Polycystic liver disease
64 Non-alcoholic steatohepatitis (NASH)

HEPATIC TUMOURS

50 Angiosarcoma
17 Cholangiocarcinoma
18 Fibrolamellar hepatoma
16 Hepatocellular carcinoma
19 Metastatic tumour
53 Hepatic tumour—other

METABOLIC DISORDERS

20 Alpha I anti-trypsin deficiency
28 Crigler-Najjar syndrome
21 Glycogen storage disease
23 Hemochromatosis
27 Hyperlipoproteinemia type 2
24 Niemann-Pick
26 Phenylketonuria
25 Protoporphyria
29 Tyrosinemia
22 Wilson's disease
34 Metabolic disorder—other

OTHER PRIMARY DIAGNOSIS

30 Congenital hepatic fibrosis
31 Caroli's disease
32 Cystic disorders
52 Thrombosed hepatic artery
98 Unknown/missing
99 Other (specify) ________________________________

Formulaire disponible en français.

Canadian Organ Replacement Register
Liver Transplant Follow-up Form

SEND THIS CONFIDENTIAL INFORMATION TO:
Canadian Organ Replacement Register (CORR)
Canadian Institute for Health Information
4110 Yonge Street, Suite 300
Toronto, ON M2P 2B7
Tel.: 416-481-2002

SECTION A—RECIPIENT INFORMATION

Transplant hospital ______________________________
(name and city)

Patient ID ______________________________

Health card number ______________________________

Prov. or terr. of health card ______________________________

Last name ____________ First/middle name ____________

Former name ______________________________

Address (city) ______________________________

Province ____________ Postal code ____________

Date of birth |___|___|/|___|___|___|/|___|___|___|___| (DD/MON/YYYY)

Affix patient label, if available.

SECTION B—HEPATITIS B POST-TRANSPLANT INFORMATION

For transplant patients who have been diagnosed with hepatitis B (as per primary diagnosis), please complete on December 31 of each year, or at time of death.

Transplant date: |___|___|/|___|___|___|/|___|___|___|___|(DD/MON/YYYY)

Recurrent disease: ☐ No ☐ Yes ➔ Please check disease severity:*

☐ Mild
☐ Moderate
☐ Severe

* *Mild: asymptomatic*
Moderate: with symptoms or signs of liver disease (e.g. jaundice, fatigue)
Severe: graft failure, cirrhosis, fibrosing cholestatic disease, signs of portal hypertension

Date of recurrence |___|___|/|___|___|___|/|___|___|___|___| (DD/MON/YYYY)

Detectable HBV DNA ☐ Yes ☐ No ☐ Not done in calendar year

Current therapy
H-BIg ☐ Yes ☐ No
Lamivudine ☐ Yes ☐ No
Other (specify) ______________________

SECTION C—HEPATITIS C POST-TRANSPLANT INFORMATION

For transplant patients who have been diagnosed with hepatitis C (as per primary diagnosis), please complete on December 31 of each year, or at timeof death.

Transplant date: |___|___|/|___|___|___|/|___|___|___|___|(DD/MON/YYYY)

Recurrent disease: ☐ No ☐ Yes ➔ Please check disease severity:*

☐ Mild
☐ Moderate
☐ Severe

* *Recurrent disease and disease severity will be based on the results of a biopsy.*

Date of recurrence/biopsy

|___|___|/|___|___|___|/|___|___|___|___| (DD/MON/YYYY)

Receiving treatment during this calendar year ☐ No ☐ Yes ➔ Please check one:

☐ Prophylaxis
☐ Recurrence

SECTION D—LIVER TUMOURS POST-TRANSPLANT INFORMATION

For transplant patients who have been diagnosed with liver tumours (as per primary diagnosis), please complete on December 31 of each year, or at time of death.

Transplant date: |___|___|/|___|___|___|/|___|___|___|___| (DD/MON/YYYY)

Current status of patient—recurrence of tumour

☐ No ☐ Yes ➔ *If yes, please complete the following or send CORR a copy of form from The International Registry of Hepatic Tumors in Liver Transplantation (Baylor University Medical Center).*

Date of recurrence |___|___|/|___|___|___|/|___|___|___|___| (DD/MON/YYYY)

Tumour markers (at the time of recurrence)

☐ Alpha-fetoprotein ____________ ng/ml

☐ Chorioembryonic antigen ____________ ng/ml

First site of recurrence

☐ Liver ☐ Mediastinum ☐ Abdomen ☐ Lungs

☐ Adrenal ☐ Biopsy tract ☐ Bone ☐ Other

Treatment ______________________________

Retransplantation

☐ No ☐ Yes ➔ Date |___|___|/|___|___|___|/|___|___|___|___| (DD/MON/YYYY)

Outcome

☐ Alive free of tumour ☐ Alive with tumour

☐ Died free of tumour ☐ Died with tumour ➔ Was death tumour-related?
☐ No
☐ Yes

Canadian Institute for Health Information
Institut canadien d'information sur la santé

Formulaire disponible en français.

Form LTFU-2014

Canadian Organ Replacement Register
Recipient Outcome for Liver Transplant

SEND THIS CONFIDENTIAL INFORMATION TO:
Canadian Organ Replacement Register (CORR)
Canadian Institute for Health Information
4110 Yonge Street, Suite 300
Toronto, ON M2P 2B7
Tel.: 416-481-2002

Complete this form to reflect the situation at your facility for patient lost to follow death, graft failure, transfer or the patient being followed at another hospital.

SECTION A—RECIPIENT INFORMATION

Transplant hospital ____
(name and city)

Patient ID ____

Last name ____

First/middle name ____

Former name ____

Date of birth |___|___|/|___|___|___|/|___|___|___|___| (DD/MON/YYYY)

Health card number ____

Prov. or terr. of health card ____

Address (city) ____

Province or Territory ________________ Postal code ________________

Hospital followed at ____
(Enter only if different than transplant hospital.)

SECTION B—RECIPIENT OUTCOME

Date of Transplant|___|___|/|___|___|___|/|___|___|___|___| (DD/MON/YYYY)

Graft Number ______________________________

Patient Status (Please check one.)
Patient alive ☐ Transfer ☐ Lost to follow-up ☐ Died ☐

Transfer Hospital Name (Please check one of the following:) ☐ To **OR** From ☐

Name of Transfer Hospital:____

Date of transfer: |___|___|/|___|___|___|/|___|___|___|___| (DD/MON/YYYY)

Date of lost to follow: |___|___|/|___|___|___|/|___|___|___|___| (DD/MON/YYYY)

If deceased (Please check one of the following and enter cause of death.)
☐ Died with a functioning graft

OR

☐ Died due to graft failure (Check cause of graft failure below.)

Enter cause of death ____________ (codes on back of page)

Date of death: |___|___|/|___|___|___|/|___|___|___|___| (DD/MON/YYYY)

SECTION B—RECIPIENT OUTCOME (continued)

If alive with failed graft or died due to graft failure, please complete this section.

Date of graft failure |___|___|/|___|___|___|/|___|___|___|___| (DD/MON/YYYY)

Check cause of graft failure below:

00 ☐ Uncertain/Unknown
01 ☐ Hyperacute rejection
11 ☐ Primary non-function
14 ☐ Graft/portal vein thrombosis
15 ☐ Graft/hepatic vein thrombosis
16 ☐ Biliary tract complication
18 ☐ De novo malignancy (graft)
22 ☐ Arterial thrombosis
28 ☐ Surgical complications
30 ☐ Rejection after stopping immunosuppressive drugs
33 ☐ De nova hepatitis
63 ☐ Acute rejection
64 ☐ Chronic rejection
67 ☐ Recurrent disease
68 ☐ Infection and rejection
69 ☐ Infection of the graft
99 ☐ Other cause of graft failure (describe) ______________________

Canadian Institute for Health Information
Institut canadien d'information sur la santé

Formulaire disponible en français.

Form LTO-2014

CAUSE OF DEATH/COMORBID COMPLICATION (RECIPIENT)

GENERIC

00 Chronic renal failure—etiology uncertain

CARDIAC

11 Myocardial ischemia and infarction
12 Hyperkalemia
13 Hemorrhagic pericarditis
15 Cardiac arrest, cause unknown
16 Hypertensive cardiac failure
17 Hypokalemia
18 Fluid overload

VASCULAR

21 Pulmonary embolus
22 Cerebrovascular accident
24 Hemorrhage from graft site—specify
25 Hemorrhage from vascular access or dialysis circuit
26 Hemorrhage from ruptured vascular aneurysm (not code 22 or 23)
27 Hemorrhage from surgery (not codes 23 to 26)—specify
28 Other hemorrhage (not codes 23 to 27)
55 Vascular thrombosis
56 Pulmonary vein stenosis
57 Stent/balloon complication

INFECTION

03 Infection (bacterial)—specify site
04 Infection (viral)—specify site
05 Infection (fungal)—specify site
06 Cytomegalovirus
07 Epstein-Barr virus
08 Pneumocystic carinii pneumonia (PCP)
09 Protozoal/parasitic infection (includes toxoplasmosis)
10 Wound infection—specify site
34 Infections elsewhere (except viral hepatitis codes 41 and 42)
35 Septicemia/sepsis—specify source
36 Tuberculosis (lung)
37 Tuberculosis (elsewhere)
38 Generalized viral infection—specify viral agent
39 Peritonitis (not code 70)

GASTROINTESTINAL

02 Gastrointestinal tumour with or without perforation
20 Acute gastroenteritis with dehydration
23 Gastrointestinal hemorrhage
29 Mesenteric infarction
62 Pancreatitis
68 Perforation of peptic ulcer
70 Sclerosing (or adhesive) peritoneal disease
72 Perforation of colon/small bowel

ACCIDENT

81 Accident related to treatment
82 Accident unrelated to treatment

MISCELLANEOUS

30 Hypertension
40 Diabetic keto acidosis (DKA)
64 Cachexia
66 Malignant disease possibly induced by immunosuppressive therapy—specify primary site
67 Malignant disease (not code 66)—specify primary site
69 Dementia
90 Multi-system failure
99 Other identified cause of death—specify

RESPIRATORY

19 Acute respiratory distress syndrome (ARDS)
31 Pulmonary infection (bacterial)
32 Pulmonary infection (viral)
33 Pulmonary infection (fungal)
49 Bronchiolitis obliterans

RENAL DISEASE

47 Acute renal failure
48 Chronic renal failure
61 Uremia caused by kidney transplant failure

METABOLIC

59 Drug-related toxicity—specify drug

HEMATOLOGIC

63 Bone marrow depression
71 Thrombocytopenia
73 Thrombosis—specify

NEUROLOGIC

75 Drug neurotoxicity—specify drug
76 Status epilepticus
77 Neurologic infection—specify infectious agent

SOCIAL

50 Drug abuse (excludes alcohol abuse)
51 Patient refused further treatment
52 Suicide
53 Therapy ceased for any other reason
54 Alcohol abuse

Canadian Organ Replacement Register
Heart Transplant Recipient Registration Form

SEND THIS CONFIDENTIAL INFORMATION TO:
Canadian Organ Replacement Register (CORR)
Canadian Institute for Health Information
4110 Yonge Street, Suite 300
Toronto, ON M2P 2B7
Tel.: 416-481-2002

SECTION A—RECIPIENT INFORMATION

Transplant hospital ____________________
(name and city)

Patient ID ____________________

Last name ____________________

First/middle name ____________________

Former name ____________________

Sex ☐ Male ☐ Female ☐ Other

Blood Type ☐ A ☐ B ☐ AB ☐ O ☐ U

Race

01 ☐ Caucasian 02 ☐ Asian 03 ☐ Black 05 ☐ Indian subcontinent
08 ☐ Pacific islander 09 ☐ Aboriginal 10 ☐ Mid East/Arabian
11 ☐ Latin American 98 ☐ Unknown 99 ☐ Other/multiracial (specify) ____

Date of birth |___|___|/|___|___|___|/|___|___|___|___| (DD/MON/YYYY)

Health card number ____________________

Prov. or terr. of health card ____________________

Address (city) ____________________

Province or Territory ____________ Postal code ____________

(At time of transplant)

Recipient height ☐☐☐•☐☐☐ (cm)

(Conversion factor: 1 in. = 2.54 cm)

Recipient weight ☐☐☐•☐☐☐ (kg)

(Conversion factor: 1 lb. = 0.45 kg)

SECTION B—TRANSPLANT INFORMATION

Waiting List Information

Date patient first placed on waiting list (for this transplant)

|___|___|/|___|___|___|/|___|___|___|___| (DD/MON/YYYY)

Medical Status When First Placed on Waiting List (Please check one.)

08 ☐ Status 1—at home
04 ☐ Status 2—hospitalized
13 ☐ Status 3A—hospitalized ICU or inotropes or less than 6 months of age
14 ☐ Status 3B—hospitalized ICU or inotropes or less than 6 months of age, with rapid deterioration
06 ☐ Status 4—ICU, mechanical/ventilatory support
15 ☐ In utero

SECTION B—TRANSPLANT INFORMATION (continued)

Date moved to final list status

|___|___|/|___|___|___|/|___|___|___|___| (DD/MON/YYYY)

(Indicate date if not same as initial listing status.)

Medical Status at Time of Transplant (Please check one.)

08 ☐ Status 1—at home
04 ☐ Status 2—hospitalized
13 ☐ Status 3A—hospitalized ICU or inotropes or less than 6 months of age
14 ☐ Status 3B—hospitalized ICU or inotropes or less than 6 months of age, with rapid deterioration
06 ☐ Status 4—ICU, mechanical/ventilatory support

Date of transplant |___|___|/|___|___|___|/|___|___|___|___| (DD/MON/YYYY)

Graft number ____________________

☐ Heart transplant only OR ☐ Combination transplant

Specify other organ(s) ____________________

Please complete section B of relevant transplant recipient registration form for other organ(s).

Primary Diagnosis (Please check one.)

32 ☐ Cardiomyopathy
29 ☐ Dilated cardiomyopathy
  01 ☐ Idiopathic cardiomyopathy
  30 ☐ Other dilated (please specify)
  33 ☐ Metabolic/genetic cardiomyopathy
  34 ☐ Cardiomyopathy related to muscular dystrophy
  35 ☐ Drug-induced cardiomyopathy (chemotherapy)
12 ☐ Restrictive cardiomyopathy
31 ☐ Hypertrophic cardiomyopathy
24 ☐ Myocarditis
07 ☐ Coronary artery disease (ischemic cardiomyopathy)
04 ☐ Valvular heart disease
23 ☐ Acute myocardial infarct
15 ☐ Congenital heart disease (please specify)
  16 ☐ Congenital heart disease—acyanotic lesions
  17 ☐ Congenital heart disease—cyanotic lesions
36 ☐ Metabolic disorders
37 ☐ Cardiac tumour
38 ☐ Refractive arrhythmia
39 ☐ Muscular dystrophy
99 ☐ Other (please specify) ____________________
☐ Retransplant

Recipient Name ____

SECTION B—TRANSPLANT INFORMATION (continued)

Recipient Serology Status at Time of Transplant
(Please check one of the acceptable values: P = positive, N = negative or U = unknown.)

Hepatitis BsAg	P ☐	N ☐	U ☐	Epstein-Barr virus	P ☐	N ☐	U ☐
Hepatitis BcAb	P ☐	N ☐	U ☐	HIV	P ☐	N ☐	U ☐
Hepatitis C	P ☐	N ☐	U ☐	CMV	P ☐	N ☐	U ☐

Donor specific antibodies Y ☐ N ☐

Class I PRA current _____ % Class I PRA peak _______ %

Class II PRA current _____ % Class II PRA peak _______ %

* Methods: CDC ☐ ELISA ☐ Flow ☐ Luminex ☐ Other ☐

* The most sensitive method should be entered if more than one method is used by the laboratory

PVR: Reactive ☐ Non reactive ☐

PVR (Woods units) <4 ☐ 4–6 ☐ >6 ☐ Not done ☐

Standard crossmatch test P ☐ N ☐ U ☐

*Recipient HL A ____ ____ B ____ ____ C ____ ____ DR ____ ____ DQ ____ ____

*Note: CORR enters the lowest haplotype first.

Heterotopic transplant Y ☐ N ☐ U ☐

Risk Factors Existing at Time of Transplant
(Please check one of the acceptable values: Y = yes, N = no or U = unknown.)

Renal dysfunction	Y ☐	N ☐	U ☐	Liver dysfunction	Y ☐	N ☐	U ☐
Diabetes type 1	Y ☐	N ☐	U ☐	Diabetes type 2	Y ☐	N ☐	U ☐
Hypertension	Y ☐	N ☐	U ☐	Smoker	Y ☐	N ☐	U ☐
Hypercholesterolemia	Y ☐	N ☐	U ☐	Inotropic support	Y ☐	N ☐	U ☐
Previous cardiac surgery	Y ☐	N ☐	U ☐	Prior defibrillator	Y ☐	N ☐	U ☐
On anticoagulants	Y ☐	N ☐	U ☐	Mechanical ventilation	Y ☐	N ☐	U ☐

Mechanical Circulatory Support Device
Please indicate the devices(s) being used.

Intra-aortic balloon	Y ☐	N ☐	U ☐
ECMO	Y ☐	N ☐	U ☐
Ventricular assist device (VAD)	Y ☐	N ☐	U ☐
Total artificial heart	Y ☐	N ☐	U ☐

Total ischemic time (min) ____________________

(time between clamp on in donor and clamp off in recipient)

SECTION C—DONOR INFORMATION

☐ Living 12 ☐ Domino donor ➔ For a living or domino donor, please complete living donor profile.

01 ☐ Deceased donor

To facilitate matching, please complete the following:

Program organizing organ recovery ____

Originating OPO donor number ____

Surname stem (first 3 letters of donor surname) ______________________________

Age Years (002––130) ______ Months (001–023) ______

Days (001–030) _______ Newborn (000) _______

Sex ☐ Male ☐ Female ☐ Other

*Donor HLA A____ ____ B____ ____ C____ ____ DR____ ____ DQ____ ____

*Note: CORR enters the lowest haplotype first.

Date of cross clamp (DD/MON/YYYY) |___|___|/|___|___|___|/|___|___|___|___|

(Cross-clamp date is the same as the date of organ recovery.)

Cross-clamp time (HH/MM) |___|___|/|___|___|

Canadian Organ Replacement Register
Recipient Outcome for Heart Transplant

SEND THIS CONFIDENTIAL INFORMATION TO:
Canadian Organ Replacement Register (CORR)
Canadian Institute for Health Information
4110 Yonge Street, Suite 300
Toronto, ON M2P 2B7
Tel.: 416-481-2002

Complete this form to reflect the situation at your facility for patient lost to follow death, graft failure, transfer or the patient being followed at another hospital.

SECTION A—RECIPIENT INFORMATION

Transplant hospital ______________________________
(name and city)

Patient ID ______________________________

Last name ______________________________

First/middle name ______________________________

Former name ______________________________

Date of birth |___|___|/|___|___|___|/|___|___|___|___| (DD/MON/YYYY)

Health card number ______________________________

Prov. or terr. of health card ______________________________

Address (city) ______________________________

Province or Territory __________________ Postal code __________________

Hospital followed at ______________________________

(Enter only if different than transplant hospital.)

SECTION B—RECIPIENT OUTCOME

Date of Transplant |___|___|/|___|___|___|/|___|___|___|___| (DD/MON/YYYY)

Graft number ______________________________

Patient Status (Please check one.)

Patient alive ☐ Transfer ☐ Lost to follow ☐ Died ☐

Transfer Hospital (Please check one of the following:) ☐ To OR From ☐

Name of Transfer Hospital: ______________________________

Date of Transfer |___|___|/|___|___|___|/|___|___|___|___| (DD/MON/YYYY)

Date of lost to follow |___|___|/|___|___|___|/|___|___|___|___| (DD/MON/YYYY)

If deceased (Please check one of the following and enter cause of death.)

☐ Died with a functioning graft

OR

☐ Died due to graft failure (Check cause of graft failure below.)

Enter cause of death _____________ (codes on back of page)

Date of Death |___|___|/|___|___|___|/|___|___|___|___| (DD/MON/YYYY)

SECTION B—RECIPIENT OUTCOME (continued)

If alive with failed graft or died due to graft failure, please complete this section.

Date of graft failure |___|___|/|___|___|___|/|___|___|___|___| (DD/MON/YYYY)

Check cause of graft failure below:

00 ☐ Uncertain/Unknown

01 ☐ Hyperacute rejection

11 ☐ Primary non-function

19 ☐ Graft coronary artery disease

23 ☐ Vascular event (graft)

25 ☐ Pulmonary hypertensive/cor pulmonale

28 ☐ Surgical complications

30 ☐ Rejection after stopping immunosuppressive drugs

63 ☐ Acute rejection

64 ☐ Chronic rejection

66 ☐ Rejection secondary to non-compliance

67 ☐ Recurrent primary disease

68 ☐ Infection and rejection

69 ☐ Infection of the graft

70 ☐ Systemic hypertension

71 ☐ Electrolyte disturbance (Please specify) ________________________

72 ☐ Pericarditis

73 ☐ Pericardial effusion

99 ☐ Other cause of graft failure (describe) ________________________

CAUSE OF DEATH/COMORBID COMPLICATION (RECIPIENT)

GENERIC
00 Cause of death uncertain/not determined

CARDIAC
11 Myocardial ischemia and infarction
12 Hyperkalemia
13 Hemorrhagic pericarditis
14 Other causes of cardiac failure
15 Cardiac arrest, cause unknown
16 Hypertensive cardiac failure
17 Hypokalemia
18 Fluid overload

VASCULAR
21 Pulmonary embolus
22 Cerebrovascular accident
24 Hemorrhage from graft site—specify
25 Hemorrhage from vascular access or dialysis circuit
26 Hemorrhage from ruptured vascular aneurysm (not code 22 or 23)
27 Hemorrhage from surgery (not codes 23 to 26)—specify
28 Other hemorrhage (not codes 23 to 27)
55 Vascular thrombosis
56 Pulmonary vein stenosis
57 Stent/balloon complication

INFECTIONS
03 Infection (bacterial)—specify site
04 Infection (viral)—specify site
05 Infection (fungal)—specify site
06 Cytomegalovirus
07 Epstein-Barr virus
08 Pneumocystic carinii pneumonia (PCP)
09 Protozoal/parasitic infection (includes toxoplasmosis)
10 Wound infection—specify site
34 Infections elsewhere (except viral hepatitis codes 41 and 42)
35 Septicemia/sepsis—specify source
36 Tuberculosis (lung)
37 Tuberculosis (elsewhere)
38 Generalized viral infection—specify viral agent
39 Peritonitis (not code 70)

LIVER DISEASE
41 Liver, due to hepatitis B virus
42 Liver, other viral hepatitis
43 Liver, drug toxicity—specify drug
44 Cirrhosis, not viral
45 Cystic liver disease
46 Liver failure, cause unknown
74 Liver, due to hepatitis C virus

GASTROINTESTINAL
02 Gastrointestinal tumour with or without perforation
20 Acute gastroenteritis with dehydration
23 Gastrointestinal hemorrhage
29 Mesenteric infarction
62 Pancreatitis
68 Perforation of peptic ulcer
70 Sclerosing (or adhesive) peritoneal disease
72 Perforation of colon/small bowel

SOCIAL
50 Drug abuse (excludes alcohol abuse)
51 Patient refused further treatment
52 Suicide
53 Therapy ceased for any other reason
54 Alcohol abuse

ACCIDENT
81 Accident related to treatment
82 Accident unrelated to treatment

MISCELLANEOUS
30 Hypertension
40 Diabetic keto acidosis (DKA)
64 Cachexia
66 Malignant disease possibly induced by immunosuppressive therapy—specify primary site
67 Malignant disease (not code 66)—specify primary site
69 Dementia
90 Multi-system failure
99 Other identified cause of death—specify

RESPIRATORY
19 Acute respiratory distress syndrome (ARDS)
31 Pulmonary infection (bacterial)
32 Pulmonary infection (viral)
33 Pulmonary infection (fungal)
49 Bronchiolitis obliterans

RENAL DISEASE
47 Acute renal failure
48 Chronic renal failure
61 Uremia caused by kidney transplant failure

METABOLIC
59 Drug-related toxicity—specify drug

HEMATOLOGIC
63 Bone marrow depression
71 Thrombocytopenia
73 Thrombosis—specify

NEUROLOGIC
75 Drug neurotoxicity—specify drug
76 Status epilepticus
77 Neurologic infection—specify infectious agent

Canadian Organ Replacement Register Lung/Heart-Lung Transplant Recipient Registration Form

SEND THIS CONFIDENTIAL INFORMATION TO:
Canadian Organ Replacement Register (CORR)
Canadian Institute for Health Information
4110 Yonge Street, Suite 300
Toronto, ON M2P 2B7
Tel.: 416-481-2002

SECTION A—RECIPIENT INFORMATION

Transplant hospital ______________________________
(name and city)

Patient ID ______________________________

Last name ________________ First/middle name ______________________

Former name ______________________________

Sex ☐ Male ☐ Female ☐ Other

Blood type ☐ A ☐ B ☐ AB ☐ O ☐ U

Race

01 ☐ Caucasian 02 ☐ Asian 03 ☐ Black 05 ☐ Indian subcontinent

08 ☐ Pacific islander 09 ☐ Aboriginal 10 ☐ Mid East/Arabian

11 ☐ Latin American 98 ☐ Unknown 99 ☐ Other/multiracial __________

Date of birth |___|___|/|___|___|___|/|___|___|___|___| (DD/MON/YYYY)

Health card number ______________________________

Prov. or terr. of health card ______________________________

Address (city) ______________________________

Province or territory ____________________ Postal code ______________
(At time of transplant)

Recipient height ☐☐☐•☐☐☐ (cm)

(Conversion factor: 1 in. = 2.54 cm)

Recipient weight ☐☐☐•☐☐☐ (kg)

(Conversion factor: 1 lb. = 0.45 kg)

SECTION B—TRANSPLANT INFORMATION

Waiting List Information

Date patient first placed on waiting list
(for this transplant)

|___|___|/|___|___|___|/|___|___|___|___| (DD/MON/YYYY)

Medical Status When First Placed on Waiting List (Please check one.)

00 ☐ Status 0—on hold

09 ☐ Status 1—stable and waiting

10 ☐ Status 2—rapid decompensation

Date moved to final list status

|___|___|/|___|___|___|/|___|___|___|___| (DD/MON/YYYY)
(Indicate date if not same as initial listing status.)

Medical Status at Time of Transplant (Please check one.)

09 ☐ Status 1—stable and waiting 10 ☐ Status 2—rapid decompensation

SECTION B—TRANSPLANT INFORMATION (continued)

Date of transplant |___|___|/|___|___|___|/|___|___|___|___| (DD/MON/YYYY)

Graft number ______________________

☐ Single lung OR ☐ Bilateral lung OR ☐ Heart lung

☐ Other combination transplant

Specify other organs(s) ______________________

(Please complete section B of relevant transplant recipient registration form for other organs.)

Primary Diagnosis (Please check one.)

08 ☐ Eisenmenger's syndrome
11 ☐ Idiopathic pulmonary fibrosis
19 ☐ Alpha I antitrypsin deficiency
26 ☐ Sarcoidosis
13 ☐ Emphysema
20 ☐ Cystic fibrosis
27 ☐ Asbestosis
17 ☐ Primary pulmonary hypertension
22 ☐ Bronchiectasis
18 ☐ Chronic obstructive lung disease
28 ☐ Bronchiolitis obliterans
15 ☐ Lung failure due to congenital disease
99 ☐ Other (please specify) ______________________________
☐ Retransplant
32 ☐ Cardiomyopathy - not specified
98 ☐ Unknown

Recipient Serology Status at Time of Transplant

(Please check one of the acceptable values: P = positive, N = negative or U = unknown.)

Hepatitis BsAg	P ☐	N ☐	U ☐	Epstein-Barr virus	P ☐	N ☐	U ☐
Hepatitis BcAb	P ☐	N ☐	U ☐	HIV	P ☐	N ☐	U ☐
Hepatitis C	P ☐	N ☐	U ☐	CMV	P ☐	N ☐	U ☐

Donor specific antibodies Y ☐ N ☐

Class I PRA current _____ % Class I PRA peak _______%

Class II PRA current _____ % Class II PRA peak _______%

* Methods: CDC ☐ ELISA ☐ Flow ☐ Luminex ☐ Other ☐

* The most sensitive method should be entered if more than one method is used by the laboratory

PVR:

Reactive ☐ Non reactive ☐ PVR (Woods units): <4 ☐ 4–6 ☐ >6 ☐ Not done ☐

Standard crossmatch test P ☐ N ☐ U ☐

*Recipient HLA: A____ ____ B____ ____ C____ ____ DR____ ____ DQ____ ____

*Note: CORR enters the lowest haplotype first.

Recipient name ____

SECTION B—TRANSPLANT INFORMATION (continued)

Risk Factors Existing at Time of Transplant

(Please check one of the acceptable values: Y = yes, N = no or U = unknown.)

Renal dysfunction	Y ☐	N ☐	U ☐	Liver dysfunction	Y ☐	N ☐	U ☐
Diabetes type 1	Y ☐	N ☐	U ☐	Diabetes type 2	Y ☐	N ☐	U ☐
Hypertension	Y ☐	N ☐	U ☐	Mechanical ventilation	Y ☐	N ☐	U ☐
Non-ambulatory status	Y ☐	N ☐	U ☐	On anticoagulants	Y ☐	N ☐	U ☐
Other organ dysfunction	Y ☐	N ☐	U ☐	Previous thoracic surgery	Y ☐	N ☐	U ☐
Multi-resistant pathogen	Y ☐	N ☐	U ☐				

SECTION C—DONOR INFORMATION

☐ Living 12 ☐ Domino donor → For a living or domino donor, please complete a living donor profile and attach to this form.

01 ☐ Deceased donor

To facilitate matching, please complete the following:

Program organizing organ recovery ___________________________________

Originating OPO donor number _____________________________________

Donor ☐ Rt. lung ☐ Lt. lung ☐ Heart lung ☐ Both lungs

Surname stem (first 3 letters of donor surname) _________________________

Age Years (002–130) ______ Months (001–023) ______

Days (001–030) ______ Newborn (000) _________

Sex ☐ Male ☐ Female ☐ Other

*Donor HLA A____ ____ B____ ____ C____ ____ DR____ ____ DQ____ ____

*Note: CORR enters the lowest haplotype first.

Date of cross clamp (DD/MON/YYYY)

|___|___|/|___|___|___|/|___|___|___|___|

(Cross clamp date is the same as the date of organ recovery.)

Cross clamp time (HH/MM) |___|___|/|___|___|

Formulaire disponible en français.

Canadian Organ Replacement Register Recipient Outcome for Lung/ Heart-Lung Transplant

SEND THIS CONFIDENTIAL INFORMATION TO:
Canadian Organ Replacement Register (CORR)
Canadian Institute for Health Information
4110 Yonge Street, Suite 300
Toronto, ON M2P 2B7
Tel.: 416-481-2002

CORR RCITO

Complete this form to reflect the situation at your facility for patient lost to follow death, graft failure, transfer or the patient being followed at another hospital.

SECTION A—RECIPIENT INFORMATION

Transplant hospital ______________________________
(name and city)

Patient ID ______________________________

Last name ______________________________

First/middle name ______________________________

Former name ______________________________

Date of birth |___|___|/|___|___|___|/|___|___|___|___| (DD/MON/YYYY)

Health card number ______________________________

Prov. or terr. of health card ______________________________

Address (city) ______________________________

Province or Territory ________________ Postal code ________________

Hospital followed at ______________________________

(Enter only if different than transplant hospital.)

SECTION B—RECIPIENT OUTCOME

Date of Transplant |___|___|/|___|___|___|/|___|___|___|___| (DD/MON/YYYY)

Graft number ______________________________

Patient Status (Please check one.)

Patient alive ☐ Transfer ☐ Lost to follow ☐ Died ☐

Transfer Hospital (Please check one of the following:) ☐ To OR From ☐

Name of Transfer Hospital: ______________________________

Date of Transfer |___|___|/|___|___|___|/|___|___|___|___| (DD/MON/YYYY)

Date of lost to follow |___|___|/|___|___|___|/|___|___|___|___| (DD/MON/YYYY)

If deceased (Please check one of the following and enter cause of death.)

☐ Died with a functioning graft

OR

☐ Died due to graft failure (Check cause of graft failure below.)

Enter cause of death ________________ (codes on back of page)

Date of Death |___|___|/|___|___|___|/|___|___|___|___| (DD/MON/YYYY)

SECTION B—RECIPIENT OUTCOME (continued)

If alive with failed graft or died due to graft failure, please complete this section.

Date of graft failure |___|___|/|___|___|___|/|___|___|___|___| (DD/MON/YYYY)

Check cause of graft failure below:

00 ☐ Uncertain/Unknown

01 ☐ Hyperacute rejection

11 ☐ Primary non-function / reperfusion injury

18 ☐ De novo malignancy

19 ☐ Graft coronary artery disease

23 ☐ Vascular thrombosis (graft)

24 ☐ Bronchiolitis obliterans

25 ☐ Pulmonary hypertension / Cor Pulmonale

28 ☐ Surgical complication

29 ☐ Large airway complications

37 ☐ Acute respiratory distress syndrome (ARDS)

63 ☐ Acute rejection

64 ☐ Chronic rejection

67 ☐ Recurrent disease

68 ☐ Infection and rejection

69 ☐ Infection of the graft

99 ☐ Other cause of graft failure (describe) ________________

CAUSE OF DEATH/COMORBID COMPLICATION (RECIPIENT)

GENERIC
- 00 Cause of death uncertain/not determined

CARDIAC
- 11 Myocardial ischemia and infarction
- 12 Hyperkalemia
- 13 Hemorrhagic pericarditis
- 14 Other causes of cardiac failure
- 15 Cardiac arrest, cause unknown
- 16 Hypertensive cardiac failure
- 17 Hypokalemia
- 18 Fluid overload

VASCULAR
- 21 Pulmonary embolus
- 22 Cerebrovascular accident
- 24 Hemorrhage from graft site—specify
- 25 Hemorrhage from vascular access or dialysis circuit
- 26 Hemorrhage from ruptured vascular aneurysm (not code 22 or 23)
- 27 Hemorrhage from surgery (not codes 23 to 26)—specify
- 28 Other hemorrhage (not codes 23 to 27)
- 55 Vascular thrombosis
- 56 Pulmonary vein stenosis
- 57 Stent/balloon complication

INFECTIONS
- 03 Infection (bacterial)—specify site
- 04 Infection (viral)—specify site
- 05 Infection (fungal)—specify site
- 06 Cytomegalovirus
- 07 Epstein-Barr virus
- 08 Pneumocystic carinii pneumonia (PCP)
- 09 Protozoal/parasitic infection (includes toxoplasmosis)
- 10 Wound infection—specify site
- 34 Infections elsewhere (except viral hepatitis codes 41 and 42)
- 35 Septicemia/sepsis—specify source
- 36 Tuberculosis (lung)
- 37 Tuberculosis (elsewhere)
- 38 Generalized viral infection—specify viral agent
- 39 Peritonitis (not code 70)

LIVER DISEASE
- 41 Liver, due to hepatitis B virus
- 42 Liver, due to other viral hepatitis
- 43 Liver, drug toxicity—specify drug
- 44 Cirrhosis, not viral
- 45 Cystic liver disease
- 46 Liver failure, cause unknown
- 74 Liver, due to hepatitis C virus

GASTROINTESTINAL
- 02 Gastrointestinal tumour with or without perforation
- 20 Acute gastroenteritis with dehydration
- 23 Gastrointestinal hemorrhage
- 29 Mesenteric infarction
- 62 Pancreatitis
- 68 Perforation of peptic ulcer
- 70 Sclerosing (or adhesive) peritoneal disease
- 72 Perforation of colon/small bowel

SOCIAL
- 50 Drug abuse (excludes alcohol abuse)
- 51 Patient refused further treatment
- 52 Suicide
- 53 Therapy ceased for any other reason
- 54 Alcohol abuse

ACCIDENT
- 81 Accident related to treatment
- 82 Accident unrelated to treatment

MISCELLANEOUS
- 30 Hypertension
- 40 Diabetic keto acidosis (DKA)
- 64 Cachexia
- 66 Malignant disease possibly induced by immunosuppressive therapy—specify primary site
- 67 Malignant disease (not code 66)—specify primary site
- 69 Dementia
- 90 Multi-system failure
- 99 Other identified cause of death—specify

RESPIRATORY
- 19 Acute respiratory distress syndrome (ARDS)
- 31 Pulmonary infection (bacterial)
- 32 Pulmonary infection (viral)
- 33 Pulmonary infection (fungal)
- 49 Bronchiolitis obliterans

RENAL DISEASE
- 47 Acute renal failure
- 48 Chronic renal failure
- 61 Uremia caused by kidney transplant failure

METABOLIC
- 59 Drug-related toxicity—specify drug

HEMATOLOGIC
- 63 Bone marrow depression
- 71 Thrombocytopenia
- 73 Thrombosis—specify

NEUROLOGIC
- 75 Drug neurotoxicity—specify drug
- 76 Status epilepticus
- 77 Neurologic infection—specify infectious agent

Formulaire disponible en français.

Canadian Organ Replacement Register Pancreas Transplant Recipient Registration Form

SEND THIS CONFIDENTIAL INFORMATION TO:
Canadian Organ Replacement Register (CORR)
Canadian Institute for Health Information
4110 Yonge Street, Suite 300
Toronto, ON M2P 2B7
Tel.: 416-481-2002

CORR RCITO

SECTION A—RECIPIENT INFORMATION

Transplant hospital ____
(name and city)

Patient ID ____

Last name __________________________ First/middle name ______________

Former name ____

Sex ☐ Male ☐ Female ☐ Other

Blood Type ☐ A ☐ B ☐ AB ☐ O ☐ U

Race

01 ☐ Caucasian 02 ☐ Asian 03 ☐ Black 05 ☐ Indian subcontinent

08 ☐ Pacific islander 09 ☐ Aboriginal 10 ☐ Mid East/Arabian

11 ☐ Latin American 98 ☐ Unknown 99 ☐ Other/multiracial______

Date of Birth |___|___|/|___|___|/|___|___|___|___| (DD/MM/YYYYY)

Health card number ____

Prov. or terr. of health card ______________________________________

Address (city) ____

Province or territory ____________________ Postal code ________________

(At time of transplant)

Recipient height ☐☐☐•☐☐☐ (cm)

(Conversion factors: 1 in. = 2.54 cm)

Recipient weight ☐☐☐•☐☐☐ (cm)

(Conversion factors: 1 lb. = 0.45 kg)

SECTION B—TRANSPLANT INFORMATION

Waiting List Information

Date patient first placed on waiting list

(for this transplant) |___|___|/|___|___|/|___|___|___|___| (DD/MM/YYYY)

Date of Transplant |___|___|/|___|___|/|___|___|___|___| (DD/MM/YYYY)

Graft number ____

☐ Pancreas transplant only **OR** ☐ Combination transplant

Specify other organ(s) ____

Please complete Section B of relevant transplant recipient registration form for other organ(s).

SECTION B—TRANSPLANT INFORMATION (continued)

Type of Pancreas

50 ☐ Whole pancreas
53 ☐ Exocrine drainage (enteric)
51 ☐ Segmental—no polymer occlusion
54 ☐ Exocrine drainage (urinary)
52 ☐ Islet cells
55 ☐ Wirsung obstruction with polymer

Primary Diagnosis for Pancreas Failure (check one)

04 ☐ Cystic fibrosis 01 ☐ Chronic pancreatitis 05 ☐ Trauma

02 ☐ Diabetes type I 06 ☐ Diabetes type 2

07 ☐ Pancreatic cancer 08 ☐ Bile duct cancer

03 ☐ Pancreatectomy 99 ☐ Other (describe) ___________________________

☐ Retransplant

Recipient Serology Status at Time of Transplant
(Please check one of the acceptable values: P = positive, N = negative or U = unknown.)

Hepatitis BsAg P ☐ N ☐ U ☐ Epstein-Barr virus P ☐ N ☐ U ☐

Hepatitis BcAb P ☐ N ☐ U ☐ HIV P ☐ N ☐ U ☐

Hepatitis C P ☐ N ☐ U ☐ CMV P ☐ N ☐ U ☐

Donor specific antibodies Y ☐ N ☐

Class I PRA current_____ % Class I PRA peak _______%

Class II PRA current_____ % Class II PRA peak _______%

* Methods: CDC ☐ ELISA ☐ Flow ☐ Luminex ☐ Other ☐

* The most sensitive method should be entered if more than one method is used by the laboratory.

Standard crossmatch test P ☐ N ☐ U ☐

*Recipient HLA A____ ____ B____ ____ C____ ____ DR____ ____ DQ____ ____
*Note: CORR enters the lowest haplotype first.

Risk Factors Existing at Time of Transplant

(Please check one of the acceptable values: Y = yes, N = no or U = unknown.)

Cardiovascular disease Y ☐ N ☐ U ☐ Has kidney failed? Y ☐ N ☐ U ☐

Cerebrovascular disease Y ☐ N ☐ U ☐ Was dialysis required? Y ☐ N ☐ U ☐

Peripheral vascular disease Y ☐ N ☐ U ☐ Diabetic nephropathy Y ☐ N ☐ U ☐

Diabetic retinopathy Y ☐ N ☐ U ☐ Diabetic neuropathy Y ☐ N ☐ U ☐

Family history of diabetes Y ☐ N ☐ U ☐

No. of years on insulin ________________

PANCREAS ONLY

Warm ischemic time (min): |___|___|___|

Rewarm time (min): |___|___|___|

Cold ischemic time (min): |___|___|___|

ISLET CELL ONLY

Cold ischemic time (min): |___|___|___|

Digestion time (min): |___|___|___|

Culture time(hours): |___|___|

Total ischemic time (min): |___|___|___|

Canadian Institute for Health Information
Institut canadien d'information sur la santé
CIHI ICIS

Formulaire disponible en français.

Form PT-2014

Recipient name ____

SECTION C—DONOR INFORMATION

☐ Living   12 ☐ Domino donor ➔ For a living or domino donor, please complete a living donor profile and attach to this form.

13 ☐ Autograft (islet)   14 ☐ Fetal tissue (islet cells)

01 ☐ Deceased donor

To facilitate matching, please complete the following:

Program organizing organ recovery ____

Originating OPO donor number ____

Surname stem ______________________ (Please enter the first 3 letters of the donor surname.)

Age   Years (002–130) __________________   Months (001–023) _________________

Days (001–030) __________________   Newborn (000) ___________________

Sex   ☐ Male   ☐ Female   ☐ Other

***Donor HLA**   A____ ____   B____ ____   C____ ____   DR____ ____   DQ____ ____

*Note: CORR enters the lowest haplotype first.

Date of cross clamp (DD/MON/YYYY) |___|___|/|___|___|___|/|___|___|___|___|

(Cross-clamp date is the same as the date of organ recovery.)

Cross-clamp time (HH/MM) |___|___|/|___|___|

Canadian Organ Replacement Register
Recipient Outcome for Pancreas Transplant

SEND THIS CONFIDENTIAL INFORMATION TO:
Canadian Organ Replacement Register (CORR)
Canadian Institute for Health Information
4110 Yonge Street, Suite 300
Toronto, ON M2P 2B7
Tel.: 416-481-2002

Complete this form to reflect the situation at your facility for patient lost to follow death, graft failure, transfer or the patient being followed at another hospital.

SECTION A—RECIPIENT INFORMATION

Transplant hospital ____________________
(name and city)

Patient ID ____________________

Last name ____________________

First/middle name ____________________

Former name ____________________

Date of birth |___|___|/|___|___|___|/|___|___|___|___| (DD/MON/YYYY)

Health card number ____________________

Prov. or terr. of health card ____________________

Address (city) ____________________

Province or Territory ____________ Postal code ____________

Hospital followed at ____________________
(Enter only if different than transplant hospital.)

SECTION B—RECIPIENT OUTCOME

Date of Transplant |___|___|/|___|___|___|/|___|___|___|___| (DD/MON/YYYY)

Graft Number ____________________

Patient Status (Please check one.)
Requires insulin ☐ Yes ☐ No

Patient alive ☐ Transfer ☐ Lost to follow-up ☐ Died ☐

Transfer Hospital Name (Please check one of the following:) ☐ To OR From ☐

Name of Transfer Hospital:____________________

Date of transfer: |___|___|/|___|___|___|/|___|___|___|___| (DD/MON/YYYY)

Date of lost to follow: |___|___|/|___|___|___|/|___|___|___|___| (DD/MON/YYYY)

If deceased (Please check one of the following and enter cause of death.)
☐ Died with a functioning graft

OR
☐ Died due to graft failure (Check cause of graft failure below.)

Enter cause of death ____________ (codes on back of page)

Date of death:

|___|___|/|___|___|___|/|___|___|___|___| (DD/MON/YYYY)

SECTION B—RECIPIENT OUTCOME (continued)

If alive with failed graft or died due to graft failure, please complete this section.

Date of graft failure

|___|___|/|___|___|___|/|___|___|___|___| (DD/MON/YYYY)

Check cause of graft failure below:

- 00 ☐ Uncertain/Unknown
- 01 ☐ Hyperacute rejection
- 11 ☐ Primary non-function
- 18 ☐ De novo malignancy (graft)
- 20 ☐ Pancreatitis
- 23 ☐ Vascular thrombosis (graft)
- 28 ☐ Surgical complications
- 63 ☐ Acute rejection
- 64 ☐ Chronic rejection
- 67 ☐ Recurrent disease
- 68 ☐ Infection and rejection
- 69 ☐ Infection of graft
- 99 ☐ Other cause of graft failure (describe) ____________________

Canadian Institute for Health Information
Institut canadien d'information sur la santé

Formulaire disponible en français.

Form PTO-2014

CAUSE OF DEATH/COMORBID COMPLICATION (RECIPIENT)

GENERIC
- 00 Cause of death uncertain/not determined

CARDIAC
- 11 Myocardial ischemia and infarction
- 12 Hyperkalemia
- 13 Hemorrhagic pericarditis
- 14 Other causes of cardiac failure
- 15 Cardiac arrest, cause unknown
- 16 Hypertensive cardiac failure
- 17 Hypokalemia
- 18 Fluid overload

VASCULAR
- 21 Pulmonary embolus
- 22 Cerebrovascular accident
- 24 Hemorrhage from graft site—specify
- 25 Hemorrhage from vascular access or dialysis circuit
- 26 Hemorrhage from ruptured vascular aneurysm (not code 22 or 23)
- 27 Hemorrhage from surgery (not codes 23 to 26)—specify
- 28 Other hemorrhage (not codes 23 to 27)
- 55 Vascular thrombosis
- 56 Pulmonary vein stenosis
- 57 Stent/balloon complication

INFECTION
- 03 Infection (bacterial)—specify site
- 04 Infection (viral)—specify site
- 05 Infection (fungal)—specify site
- 06 Cytomegalovirus
- 07 Epstein-Barr virus
- 08 Pneumocystic carinii pneumonia (PCP)
- 09 Protozoal/parasitic infection (includes toxoplasmosis)
- 10 Wound infection—specify site
- 34 Infections elsewhere (except viral hepatitis codes 41and 42)
- 35 Septicemia/sepsis—specify source
- 36 Tuberculosis (lung)
- 37 Tuberculosis (elsewhere)
- 38 Generalized viral infection—specify viral agent
- 39 Peritonitis (not code 70)

LIVER DISEASE
- 41 Liver, due to hepatitis B virus
- 42 Liver, due to other viral hepatitis
- 43 Liver, drug toxicity—specify drug
- 44 Cirrhosis, not viral
- 45 Cystic liver disease
- 46 Liver failure, cause unknown
- 74 Liver, due to hepatitis C virus

GASTROINTESTINAL
- 02 Gastrointestinal tumour with or without perforation
- 20 Acute gastroenteritis with dehydration
- 23 Gastrointestinal hemorrhage
- 29 Mesenteric infarction
- 62 Pancreatitis
- 68 Perforation of peptic ulcer
- 70 Sclerosing (or adhesive) peritoneal disease
- 72 Perforation of colon/small bowel

SOCIAL
- 50 Drug abuse (excludes alcohol abuse)
- 51 Patient refused further treatment
- 52 Suicide
- 53 Therapy ceased for any other reason
- 54 Alcohol abuse

ACCIDENT
- 81 Accident related to treatment
- 82 Accident unrelated to treatment

MISCELLANEOUS
- 30 Hypertension
- 40 Diabetic keto acidosis (DKA)
- 64 Cachexia
- 66 Malignant disease possibly induced by immunosuppressive therapy—specify primary site
- 67 Malignant disease (not code 66)—specify primary site
- 69 Dementia
- 90 Multi-system failure
- 99 Other identified cause of death—specify

RESPIRATORY
- 19 Acute respiratory distress syndrome (ARDS)
- 31 Pulmonary infection (bacterial)
- 32 Pulmonary infection (viral)
- 33 Pulmonary infection (fungal)
- 49 Bronchiolitis obliterans

RENAL DISEASE
- 47 Acute renal failure
- 48 Chronic renal failure
- 61 Uremia caused by kidney transplant failure

METABOLIC
- 59 Drug-related toxicity—specify drug

HEMATOLOGIC
- 63 Bone marrow depression
- 71 Thrombocytopenia
- 73 Thrombosis—specify

NEUROLOGIC
- 75 Drug neurotoxicity—specify drug
- 76 Status epilepticus
- 77 Neurologic infection—specify infectious agent

Formulaire disponible en français.
